# Additive Manufacturing of AlSi10Mg and Ti6Al4V Lightweight Alloys via Laser Powder Bed Fusion: A Review of Heat Treatments Effects

**DOI:** 10.3390/ma15062047

**Published:** 2022-03-10

**Authors:** Emanuele Ghio, Emanuela Cerri

**Affiliations:** Department of Engineering and Architecture, University of Parma, 43124 Parma, Italy; emanuela.cerri@unipr.it

**Keywords:** additive manufacturing, laser powder bed fusion, AlSi10Mg, Ti6Al4V, heat treatments, mechanical properties, microstructural characterization, fracture mechanism, corrosion resistance

## Abstract

Laser powder bed fusion (L-PBF) is an additive manufacturing technology that is gaining increasing interest in aerospace, automotive and biomedical applications due to the possibility of processing lightweight alloys such as AlSi10Mg and Ti6Al4V. Both these alloys have microstructures and mechanical properties that are strictly related to the type of heat treatment applied after the L-PBF process. The present review aimed to summarize the state of the art in terms of the microstructural morphology and consequent mechanical performance of these materials after different heat treatments. While optimization of the post-process heat treatment is key to obtaining excellent mechanical properties, the first requirement is to manufacture high quality and fully dense samples. Therefore, effects induced by the L-PBF process parameters and build platform temperatures were also summarized. In addition, effects induced by stress relief, annealing, solution, artificial and direct aging, hot isostatic pressing, and mixed heat treatments were reviewed for AlSi10Mg and Ti6AlV samples, highlighting variations in microstructure and corrosion resistance and consequent fracture mechanisms.


**Table of Contents**


Abstract11.Introduction22.Laser-Pwoder Bed Fusion (L-PBF) Process3
Process Parameters63.L-PBFed AlSi10Mg: Microstructure123.1.As-Built Microstructure123.2.Heat-Treated Microstructure184.L-PBFed AlSi10Mg: Mechanical Properties225.L-PBFed AlSi10Mg: The corrosion Resistance306.L-PBFed Ti6Al4V: Microstructure336.1.As-Built Microstructure336.2.Heat-Treated Microstructure42
Heat Treatments Effects on α′, α and β Phases517.L-PBFed Ti6Al4V: Mechanical Properties568.L-PBFed Ti6Al4V: The Corrosion Resistance679.Conclusions679.1Microstructure and Corrosion Resistance of AlSi10Mg689.2Mechanical Properties of AlSi10Mg689.3Microstructure and Corrosion Resistance of Ti6Al4V689.4Mechanical Properties of Ti6Al4V6910.Future Trends and Prospective6911.Acronyms70
References71

## 1. Introduction

Aluminium and titanium lightweight alloys are of great interest in automotive, aerospace and biomedical fields where high performance is required [1,2,3]. The rapid development of marine, aerospace and automotive transportation has induced constant evolution in terms of safety and fuel efficiency, qualities that are met by the lightweight alloys [4,5]. AlSi10Mg and Ti6Al4V, the most studied alloys in the lightweight alloy family, found a wide range of applications thanks to the advantages of additive manufacturing (AM) and their mechanical performance and corrosion resistance. For these reasons, the present review aimed to summarize and discuss the effects induced by different heat treatments (HTs) on these alloys.

**Figure 1:**
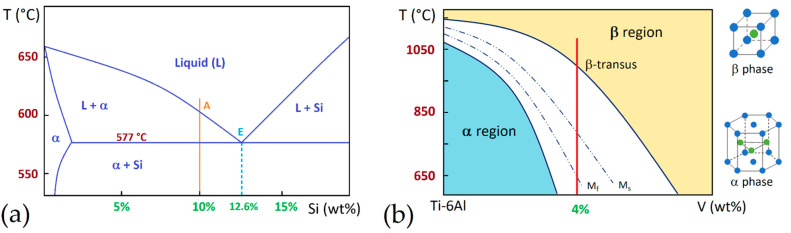
Phase diagrams of the: (**a**) AlSi10Mg where the α is related to the Al matrix; (**b**) Ti6Al4V.

AlSi10Mg is an age-hardening alloy based on the Al-Si-Mg ternary system and is characterized by low density (2.67 g/cm^3^, [6]), good mechanical properties, excellent castability and capability of being heat-treated [7,8,9]. The Al-Si phase diagram, shown in Figure 1a, highlights the fact that AlSi10Mg presents a short solidification range (ΔT ~ 40 K), making it the most common aluminium alloy used in additive manufacturing (AM) [10,11]. This phase diagram is typical of partially miscible liquids characterizing age-hardening alloy. In this scenario, the 0.2 ÷ 0.4 wt.% Mg content increases strengthening via precipitation hardening due to the precipitation of fine ε-Mg_2_Si phase during the aging heat treatment (HT, direct or T6 heat treatment) and/or during the AM process [12,13,14]. At the same time, Si is added to increase the castability and the amount of shrinkage during melt freezing, as well as to change the microstructure from rosette (low Si) to dendritic [13,15]. Wang et al. [16] emphasized significant effects on the mechanical properties of the Al-Si-Mg alloys with variation in Si (wt%).

Ti6Al4V in an α + β alloy exhibited prolonged biocompatibility, high fatigue resistance and toughness, as well as higher tensile strength and density (4.41 g/cm^3^, [6]) than AlSi10Mg alloy [17,18]. At the same time, its corrosion resistance makes it possible to use in marine and chemical industries and in the biomedical field for orthopedic, cranial and orthodontic implants [19,20,21]. A tempering treatment performed at 400–600 °C makes the Ti6Al4V alloy suitable for the manufacturing of cold engine components [22]. Figure 1b shows a portion of the Ti-6Al phase diagram, where the red iso-concentration line highlights the cooling/heating path of Ti6Al4V alloy. In the same figure, the hexagonal closed packed and the body cubic centered lattice structures of the α and β phases are illustrated, respectively. Starting from the β-region (T > $T_{\beta_{\mathrm{Tr}}}$ = ~995 °C, [17]), where the Ti alloy shows a fully β-phase microstructure, to the room temperature, the β-phase is almost completely transformed into α-phase (~90 ÷ 95%) + β-phase (~5 ÷ 10%) due to the presence of Al and V alloying elements that stabilize the hexagonal closed packed α-phase and the body centered cubic β-phase, respectively [17,23]. Considering, instead, a Ti6Al4V extra low interstitial (ELI) alloy, the previously described microstructural transformation is the same, except that the β-transus temperature is at 975 °C due to the different wt% content of alloying elements [24,25,26]. At the same time, Al and V increase elongation, tensile strength, toughness and fatigue resistance, which also depend on the α + β phases morphology at room temperature, as discussed in Section 6 [27,28].

Ti6Al4V exhibits a Widmanstätten microstructure (plate-like α + β phase) after slow cooling (furnace, cooling rate of 2 °C/s) and a martensitic microstructure after rapid cooling (water, cooling rate of 20 °C/s) due to the intersection between the cooling path and the martensitic start (Ms) line. The air cooling (cooling rate of 3.5 °C/s) induces, firstly, a martensitic transformation of the β-phase and then a diffusional transformation that reduces the volume fraction of the α′ martensite phase [29,30,31,32].

Generally, AlSi10Mg and Ti6Al4V alloys are forged or cast, followed by machining to obtain the final dimensions and shape. Owing to the large amount of material waste and the high manufacturing costs and time that characterize these conventional manufacturing processes, they have been replaced by AM technology [33,34]. In this scenario, the ability to manufacture samples with complex geometry, efficient material usage, material flexibility and dimension control are advantages that can be added at the AM field as discussed in Section 2.

The aim of the present review is to summarize and discuss the effects induced by different HTs on the most studied alloys in the AM field: AlSi10Mg and Ti6Al4V. Firstly, the as-built microstructure and consequent mechanical properties are studied, highlighting the effects of build platform (BP) pre-heating. Subsequently, by manufacturing high-quality samples characterized by excellent mechanical properties, these can effectively be optimized with post-process HTs. Secondly, the microstructure and mechanical properties obtained after different HTs are illustrated to summarize and discuss the state of the art of AlSi10Mg and Ti6Al4V alloys in the field of AM. Finally, the facture mechanisms and corrosion resistance characterizing as-built and heat-treated alloys are also reviewed.

## 2. Laser-Powder Bed Fusion (L-PBF) Process

AM technology is a process opposed to the subtractive manufacturing methodologies because it joins materials to make components from a 3D model, as defined through the ASTM 52900:2015 standard specification [35]. If low-volume and high-value objects (e.g., in aerospace and biomedical fields) are considered, the manufacturing processes belonging to the AM scenario present different advantages as opposed to the conventional subtractive manufacturing (as mentioned in Section 1) [1,2,3,33,34,36]. In this scenario, another advantage is the ability to print lightweight objects thanks to a complex software system that minimizes the part geometry after a careful engineering analysis. In parallel with the metallurgical sector, other research fields focus their attention on the sample design and the surface geometry to improve the mechanical properties of the manufactured object and the consequent advantages induced by AM [37,38].

**Figure 2:**
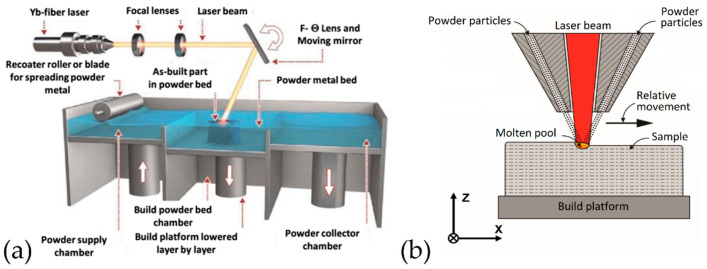
(**a**) laser powder bed fusion (L-PBF) process; (**b**) direct energy deposition (DED) process (Adapted from references [40,42]).

Focusing on these processes, the building of 3D metal physical objects takes place through the fusion or bond (e.g., melting, sintering) of the material feedstock, which is in the form of powder and/or filament/wire, joined layer by layer [35]. The different form of the material feedstock subdivides the processes in powder bed fusion (PBF) and direct energy deposition (DED). In the former printing method, a laser or electron beam energy source melts and fuses the powder following the 3D project of the component (Figure 2a), while, in the latter method, a nozzle deposits the material feedstock, as shown in Figure 2b [35,39,40,41].

The present review is based on the L-PBF AM process (Figure 2a), a technology where the powder bed, deposited on the build platform (BP) by a recoater roller or blade, is scanned and melted through a laser beam characterized by a laser power P [W], and generated by one of the following energy sources: Yb:YAG fiber, Nd:YAG, CO_2_ laser, infrared, etc. [43]. The moving mirror, controlled by a computer system, deflects the laser source according to the 3D project. Only when a layer is totally scanned and melted, the BP lowers by a quantity equal to the layer thickness (t, [mm]) and the recoater roller or blade spreads a new powder layer. This procedure is repeated until the complete 3D physical object is manufactured [44,45,46].

**Figure 3:**
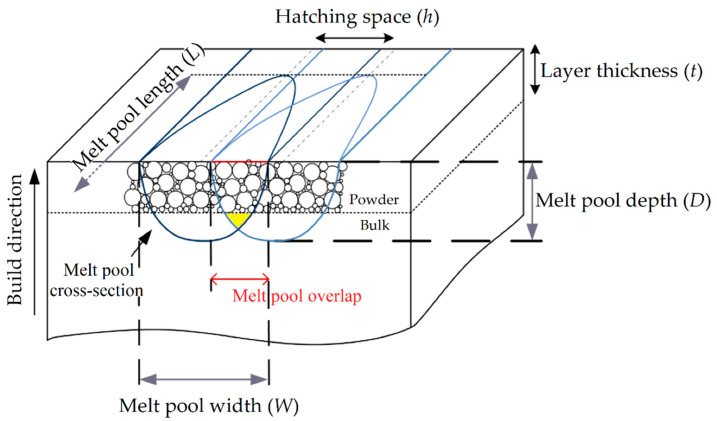
Schematic representation of the interaction between the laser beam source and the powder bed during the L-PBF process highlighting the overlap area between two adjacent laser scan tracks where the principal dimensions are labelled (Reprinted from reference [49]).

During the L-PBF process, the laser beam transmits enough energy to melt the entire layer depth and a portion of the previously solidified layer, guaranteeing the adhesion between them [47]. The molten pool (MP) depth is key to obtaining this adhesion and the absence of defects. Tang et al. [48] illustrated the following criterion to have a full melting:

(1) $${\left(\frac{h}{W}\right)}^{2}+{\left(\frac{t}{D}\right)}^{2}\leq 1$$

where *h* is the hatch spacing (mm), which is the distance between the center of one laser scan track and the consecutive one (Figure 3) [47], *W* is the width (mm), *D* is the total depth of the MP (mm), and t is the layer thickness (mm). The full melting is obtained only if Equation (1) is satisfied. As a matter of fact, the MP overlap and the consequent yellow zone shown in Figure 3 are guaranteed. Another important process parameter, in addition to *P* (W), *h* (mm) and *t* (mm) is the scan speed (*v*, (mm/s)) at which the laser source moves. Combining these parameters, the energy density function (*ED*, (J/mm^3^)) can be defined as follows:

(2) $$ED=\frac{P}{vht}$$

According to the manufacturing requirements, the ED function determines the sample’s density and, therefore, characterizes the as-built mechanical properties. Table 1 shows a wide range of process parameters used to manufacture both AlSi10Mg and Ti6Al4V alloys, highlighting whether the obtained samples are fully dense (δ ≥ 99.0%), or dense (99.0 < δ ≤ 85%) or porous (δ < 85.0%).

In this scenario, the porous Ti6Al4V samples were often manufactured for biocompatibility requirements into biomedical fields because the cell adhesion and proliferation were increased [65,66,67].

### 2.1. Process Parameters

**Figure 4:**
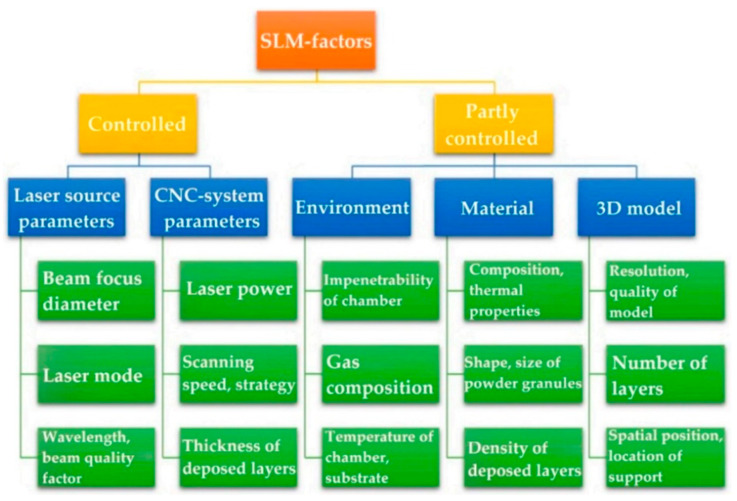
Factors correlated to the selective laser melting (or L-PBF) and subdivided into controlled and partially controlled (Reprinted from reference [69]).

The principal process parameters (*P*, *v*, *h*, *t*), which define the ED function (Equation (1)), influence microstructure and mechanical properties of AlSi10Mg and Ti6Al4V as-built samples manufactured via L-PBF process [9,14,68]. As shown in Figure 4, they are the only parameters that can be fully controlled, together with the laser source parameters, to obtain a high-quality sample. On the other hand, the other parameters such as environment, material characteristics and parameters of the 3D model can compromise its quality due to their partial controllability. Table 2 illustrates the type of defects that affect the sample’s quality.

**Figure 5:**
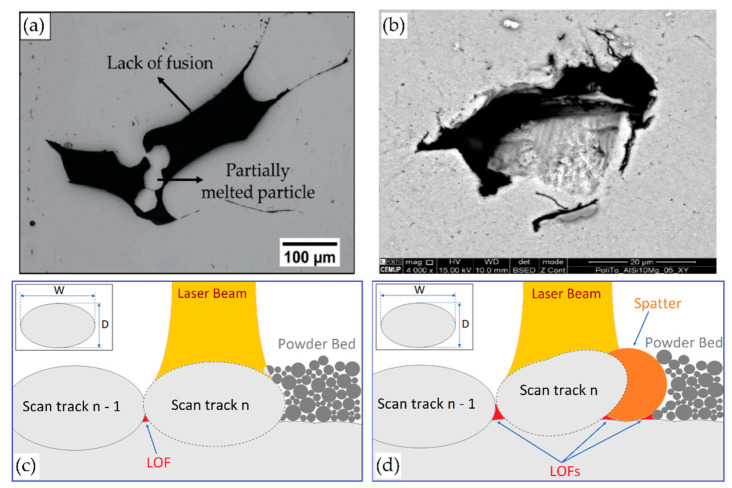
Lack of Fusion (LOF) pores classifying as pores: (**a**) with un-melted particles, (**b**) poor bonding defects (Adapted from references [95,96]). Schematic representation of the LOF formation during the L-PBF process: (**c**) lack of adequate laser scan tracks overlap; (**d**) presence of defects such as spatter.

The main types of defects present within a sample manufactured via L-PBF process are lack-of-fusion (LOF), keyhole, and gas pores; each one of these is characterized by a distinct formation mechanism and growth [51,75,84]. Focusing on LOF pores, it can be classified as a defect with partially melted powders (Figure 5a) or poor binding defect due to the insufficient melt material during solidification (Figure 5b) [94]. De facto, LOF is derived from an improper melting of the powder particles or from the MP instability (as discussed later) due to the inadequate value of the *ED* function.

**Figure 6:**
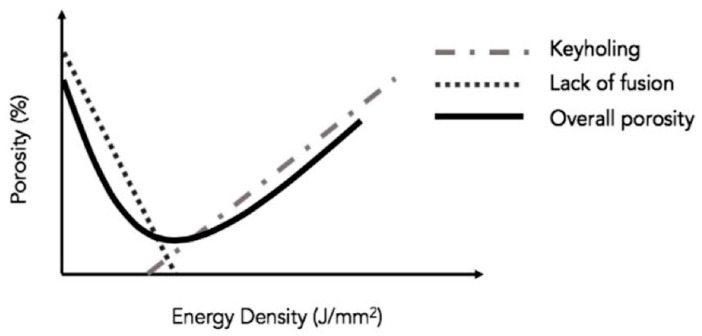
Porosity trend in relation to the *ED* and scan speed variations (Adapted from reference [97]).

Tang et al. [48] illustrated the typical trend of sample porosity in relation to the *ED* and the variation of the scan speed maintaining the laser power constant (Figure 6). In detail, the LOF pores are formed with low *ED* and high scan speed, while high *ED* and low scan speed induce a keyhole pore formation.

Darvish et al. [98], however, showed that the LOF formations are also caused by the imperfect overlap of the laser scan tracks (Figure 5c) or by the presence of spatter on the layer surface (Figure 5d).

On the other hand, the keyhole and LOF formation mechanisms are very difficult to determine due to the interaction of different physical phenomena (e.g., Maragoni effect, vaporization, recoil pressure, laser reflection) that take place within micron seconds during the rapid solidification of the MP [73]. In this scenario, Bayat et al. [73] added capillarity pressure as a physical phenomenon to determine the MP instability and the consequent keyhole formation. Thus, the presence of different cold zones characterized by high surface tension and negligible pressure recoil causes the pore’s formation. The same results were obtained by [99].

Finally, the LOF and keyhole are grouped under the name melting-related defects due to their irregular shape (Figure 5a,b), while gas pores are considered separately due to their spherical morphology (<100 μm) [51,94]. The main causes of gas pore formation (Table 2) are the presence of gas wrapped into the gas atomized powder, or the dissolution/entrapment of gas present within the build chamber [51,74,75,76,77,78,79]. Focusing on AlSi10Mg samples, the gas pores can also be caused by the H_2_O reduction during the L-PBF process due to the thermal cycles induced by the printing methodology [100]. De facto, the water follows the subsequent chemical reactions:

2H_2_O → 2H_2_ + O_2_

**Figure 7:**
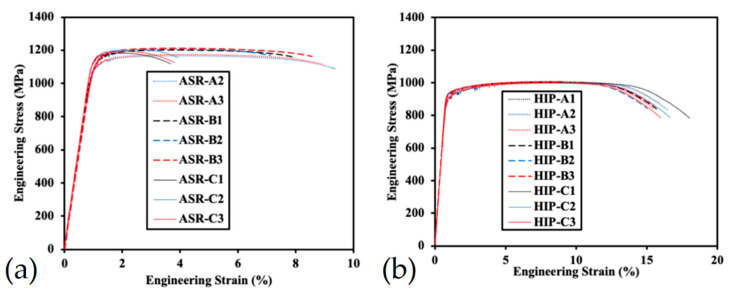
Tensile properties of heat-treated Ti6Al4V samples after stress relief (**a**) and hot isostatic pressing HTs (**b**) (Reprinted from reference [111]).

3H_2_O + 2Al → Al_2_O_3_ + 3H_2_

producing diatomic hydrogen that dissolves into monoatomic hydrogen atoms (H_2_ → 2H_absorbed_). Subsequently, the H atoms diffuse within the liquid aluminium due to their high solubility in this metallic material [101]. Considering that the partition coefficient depends on the solidification rate, the L-PBF is characterized by a greater solubility of hydrogen than the cast process because the AM process is a rapid solidification process [102]. Weingarten et al. [75] summarized that the formation of pores decreases where the hydrogen contamination of the powder is lower (<50% of pores after the drying of the powder at 200 °C) or where optimized management is used. In relation to the mechanical properties, Gong et al. [103] concluded that the tensile strength and fatigue resistance of as-built Ti6Al4V samples are not influenced by 1 vol% of gas pores but are considerably degraded with a volume fraction of 5 vol%. The same conclusions can be rewritten for AlSi10Mg as-built samples [14,104]. In this scenario, the hot isostatic pressing (HIP) HT can be used not only to densify the material but also to reduce the residual stress [105,106]. At the same time, if the fatigue resistance is increased due to the pore’s reduction, the tensile properties of Ti6Al4V samples decrease, as can be observed in Figure 7 [107,108,109,110,111]. In contrast, the ductility increases. More details and microstructural analysis will also be discussed in Section 3, Section 4, Section 6 and Section 7 for AlSi10Mg samples.

**Figure 8:**
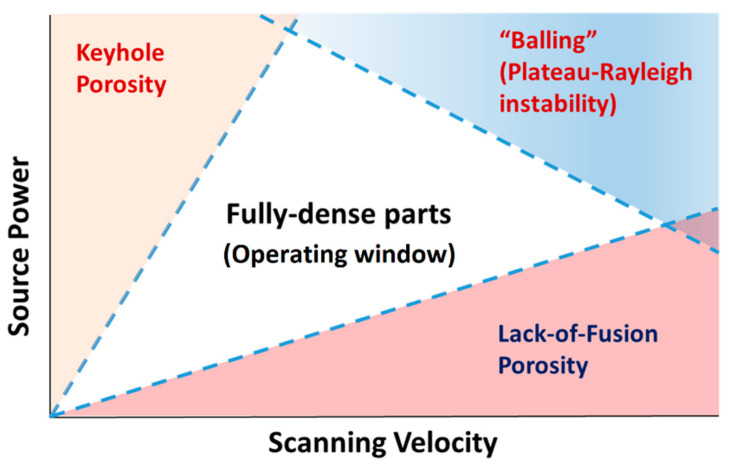
Operating window of the L-PBF process in relation to the laser beam source and scanning speed (Reprinted from reference [88]).

The best mechanical properties of the as-built sample, which are necessary to obtain excellent mechanical properties after the HT, can be reached by printing with optimized process parameters. In other words, working in the operating window shown in Figure 8 must be necessary [88]. On the other hand, this generalization neglects other important phenomena (Table 2, Figure 4) that must be considered during the L-PBF process.

Focusing on the balling phenomena and preferential evaporation defects, the main causes are strictly related to the thermal gradient and the instability of the MP [62,89,90,91,92,93]. Kruth et al. [112] suggested that the balling phenomenon takes place when the material underlying the MP does not wet due to the surface tension. As a matter of fact, the balling phenomenon occurs when the MP surface becomes larger than the surface of a sphere that contains the same volume. This situation can be prevented if the process parameters are contained in the operating window (Figure 8).

On the other hand, if the temperature of the exposed powder exceeds the temperature melting point, the evaporation phenomena occur, inducing a loss in mechanical properties of the as-built samples [82,112]. Juechther et al. [82] affirmed that the evaporation effects decrease linearly with the *ED* transferred to the MP. Masmoudi et al. [113], analyzing the build chamber atmosphere, concluded that the evaporated volume during the L-PBF process can be controlled. Generally, the inert environment (continuous Ar or Ar+ He or N gas flow, [114,115]) is used to avoid metal oxidation during the laser-powder process, particularly for Ti6Al4V and AlSi10Mg alloys that are characterized by high oxygen affinity. The build chamber environment is already high-oxidizing for the spatter particles and MP, considering an oxygen level of 1000 ppm [114,115]. On the other hand, an opportune gas flow stabilizes the depth of the MP and reduces the spatter phenomena and, consequently, oxidation [116,117]. Last but not least, the inert gas environment is an essential factor to obtain a better quality of the samples due to the pores and surface roughness reduction [118,119].

**Figure 9:**
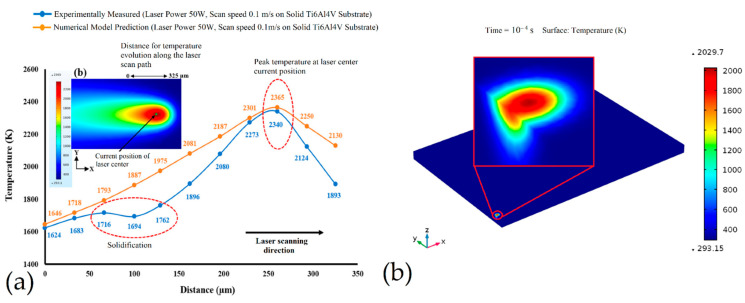
(**a**) temperature trends of a molten pool (MP) measured and calculated along the xy plane; (**b**) distribution of temperature along the xy and xz planes (Adapted from reference [121]).

The principal process parameters (*P*, *h*, *v*, *t*) also influence the MP temperature and dimension, melt lifetime and the build rate [90,120]. De facto, the MP characterized by a typical semi-ellipsoidal shape is described by a thermal gradient decreasing from the center to its boundaries (Figure 9a) [121].

**Figure 10:**
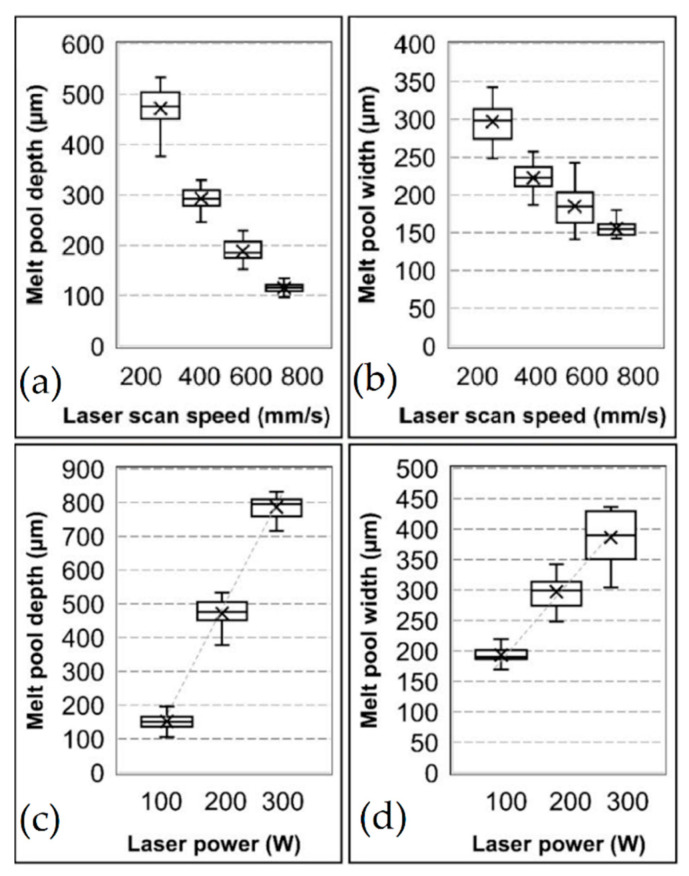
MP depth (**a**,**c**) and width (**b**,**d**) related to the: (**a**,**b**) laser scan speed (mm/s) and (**c**,**d**) laser powder (W) (Adapted from reference [124]).

Due to the conduction heat transfer, the solidified material around the MP is also invested by a thermal gradient distribution (Figure 9b), causing a heat affected zone (HAZ) and consequent microstructural effects in AlSi10Mg and Ti6Al4V samples [14,121,122]. In addition, these thermal gradients and the MP dimensions are affected by the process variation induced by the optimization of sample quality (Figure 10) [123,124].

Lastly, the *v*, *h*, and *t* variation in L-PBF process cause a variation of the build rate (*BR*, (cm^3^/h), [120]) and the consequent productivity because it is defined as follows:

(3) BR=vht,

where *v*, *h* and *t* are the scan speed (mm/s), hatch spacing (mm) and layer thickness (mm), respectively. Thus, being that the *BR* equation is the denominator of Equation (1), the *ED* function is strictly related to industrial productivity. Generally, a build rate of 5–20 cm^3^/h characterizes the L-PBF systems, but new AM machines (e.g., XLine 1000) also reach 100 cm^3^/h [125,126,127]. Table 2 shows some BR values referred to in the literature analyzed and the samples’ density reached. Shi et al. [128] showed an increase of layer thickness up to 200 μm, maintaining a Ti6Al4V sample density δ > 99.73% (fully dense). On the other hand, varying the layer thickness of AlSi10Mg and Ti6Al4V samples manufactured via L-PBF changes the mechanical properties obtained [9,32,103,128]. The use of double or quadruple lasers, which work in parallel on the same layer powder bed, or the use of skin-core scan strategy are other examples concerning the increase in the *BR* [120,129].

## 3. L-PBFed AlSi10Mg: Microstructure

### 3.1. As-Built Microstructure

**Figure 11:**
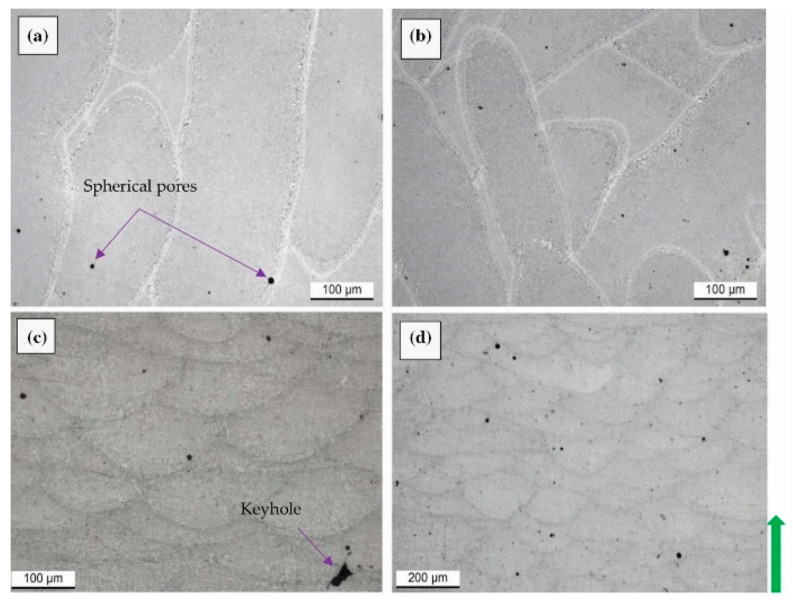
OM micrographs of the as-built AlSi10Mg samples along the xy (**a**,**b**) and xz (**c**,**d**) planes. Panels (**a**,**c**) are related to the single laser machine set-up and (**b**,**d**) to the multi laser. The green arrow indicates the build direction (Reprinted from reference [14]).

**Figure 12:**
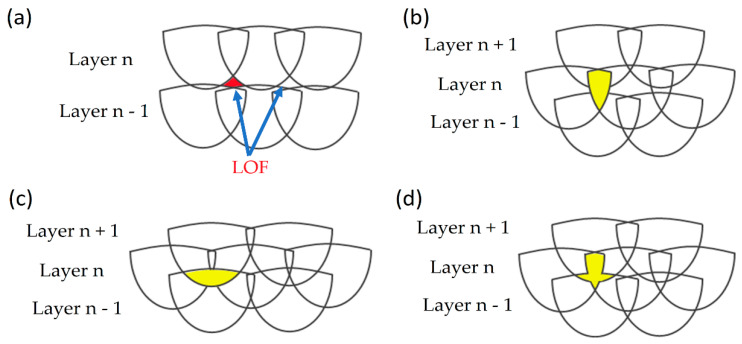
Schematic representation of: (**a**) conventional scanning strategy; (**b**) intra-layer, (**c**) inter-layer, (**d**) mixed overlapping regimes.

The as-built microstructure of the hypoeutectic AlSi10Mg alloy L-PBF-ed is shown in Figure 11, where two different machine setups are compared. Figure 11a,c illustrate the samples manufactured with a single laser, while Figure 11b,d those with multi-laser (4 × 400 W) [14]. Both optical micrographs performed along the xy plane (Figure 11a,b) show the laser scan tracks sections that are placed according to the scan strategy. At the same time, it is possible to highlight the typical semi-ellipsoidal shape of the MP as reported in Section 2. The microstructure along the build direction (Figure 11c,d) presents a typical fish-scale morphology due to the overlapping of the laser scan tracks, as shown in Figure 12 [14,130,131].

The schematic representation of different types of scanning strategies is shown in Figure 12, as also analyzed by Su et al. [131].

The same authors suggested three laser scans overlapping, different from the conventional scanning strategy (Figure 12a), to obtain a continuous track during the L-PBF process. At the same time, the full melting criterion describe in Equation (1) is satisfied. The intra- (Figure 12b), inter- (Figure 12c) and mixed (Figure 12d) laser scanning strategies tend to avoid the presence of the zone with low density (Figure 12a) generating LOF/keyhole pores, as highlighted in Figure 11c. At the same time, Figure 11a shows the spherical gas pores discussed and analyzed in Section 2.

Despite this, comparing the single and multi-laser machine set up, no microstructural differences can be emphasized, as also reported by Zhang et al. [132], who analyzed the isolated and overlapped areas through the EBSD (Electron Backscatter Diffraction) measurements

These EBSD maps show the same grain morphology in addition to the same grain size: in fact, the single laser area shows an average size of 5.72 μm, and the overlap area of 5.62 μm [132]. On the other hand, the same EBSD measurements highlight the presence of columnar grains nucleated and grown during the solidification process.

**Figure 13:**
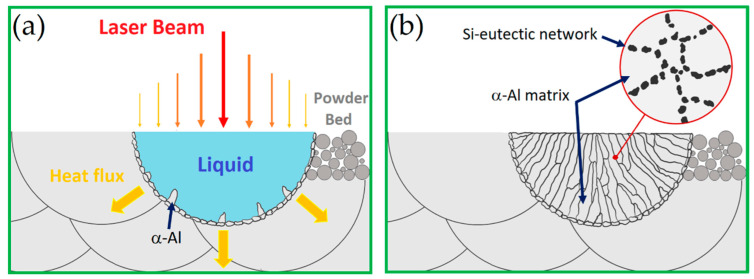
Schematic representation of the MP solidification process: (**a**) interaction between the laser beam during the melt of powder bed and the initiation of the solidification process; (**b**) final phase of the solidification process. The red circle highlights the α-Al matrix surrounded by Si-eutectic network.

De facto, during the cooling of the MP, the primary planar grains nucleate and grow at the interface between the solid and liquid phases (Figure 13a). Subsequently, the dendrites grow and compete following the direction of the heat flux, but in opposite versus along the <100> direction, as shown by other fcc metals [133]. Figure 13b illustrated the last solidification step, where the columnar grains are arranged as previously discussed, and where the Si-eutectic network is formed as highlighted within the circular area [134,135].

Lingda et al. [134], who analyzed the CET (columnar-to-equiaxed transition) into an MP, highlighted an increase in undercooling zones during the MP solidification due to the decrease in MP area during the increase in solidification time. This situation induces a competitive grain growth stage, where the equiaxed grain can also nucleate and grow. De facto, the same authors affirmed that the MP can be formed by only equiaxed grains if it reaches an undercooling of ΔT = 15K. Hadadzadeh et al. [136], correlating the CET to the thermal gradient (*G*, (K/m)) and the solidification rate (*R*, (m/s)) ratio, affirmed that the CET is promoted if the G/R decreases. Paul et al. [137] showed these grain differences within an MP through the EBSD measurements, highlighting the preferential grain growth along the <100> direction. The same authors also showed a reduction in equiaxed grain amount with the increase in the layer thickness (*t*, (mm)) and the hatch spacing (*h*, (mm)). De facto, by decreasing the *t* and *h*, the grain size decreases. Ghio et al. [9] showed, instead, an increase in the amount of the equiaxed grains with the increase in layer thickness and decrease in the hatch spacing.

**Figure 14:**
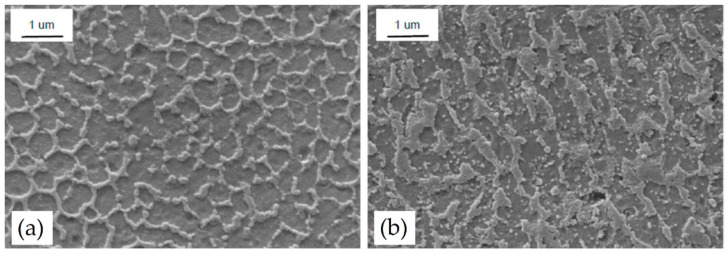
L-PBFed AlSi10Mg samples using: (**a**) a cold BP (35 °C); (**b**) pre-heated BP (200 °C) (Reprinted from reference [138]).

Figure 14 illustrates SEM micrographs of the AlSi10Mg as-built microstructure, showing the Si-eutectic network that surrounds the α-Al matrix, as analyzed by [14,87,128,138] and previously discussed in Figure 13c. The same authors highlight the presence of Si-rich precipitates within the α-Al matrix (Figure 14a) that coarsened (Figure 14b) if the pre-heated BP at 200 °C was used during the L-PBF process. This microstructural configuration is also shown by [14]. Van Cauwenbergh et al. [139] showed the presence of Si-rich precipitates in the α-Al matrix, confirming again that it is a supersaturated solid solution (SSS).

These microsegregation features, related to the SSS, are caused by the chemical composition fluctuation at the liquid/solid interface during the solidification process. It can be predicted through Brady-Fleming’s cellular microsegregation model to determine the effects induced in the AlSi10Mg L-PBFed alloy. This mathematical model describes the profile of the solid-state concentration through the following Equation (4):

(4) $$C_{s}=k_{0}C_{0}\left[\frac{a}{k_{0}-1}+\left(1-\frac{ak_{0}}{k_{0}-1}\right){\left(1-f_{s}\right)}^{k_{0}-1}\right]$$

where $k_{0}$ (-) is the equilibrium partition coefficient, $C_{0}$ (-) is the alloy solute concentration, $f_{s}$ (-) is the solid fraction, a (-) is the cellular microsegregation parameter. In this scenario, the cellular microsegregation coefficient is strictly related to the alloy characteristics (diffusion coefficient into liquid (*D_l_*, (m^2^/s)), and slope liquid (*m_l_*, (K)) and to the process conditions (thermal gradient (*G*, (K/m)) and solidification rate (*R*, (m/s)). In fact, it is defined as follows:

(5) $$a=\frac{G}{R}\left(\frac{D_{l}}{m_{l}C_{0}}\right)$$

**Figure 15:**
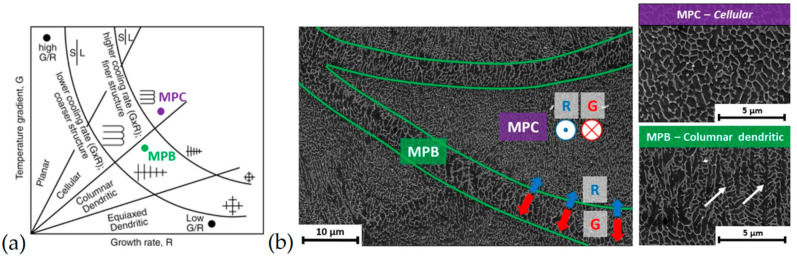
(**a**) Solidification map obtained by the G/R and G × R factors; (**b**) SEM micrographs of the MP center (MPC) and MP boundaries (MPB) (Adapted from reference [139]).

The ratio between the thermal gradient and the solidification rate also determines, firstly, the microstructures’ morphology obtained after the solidification process and, secondly, the grains size [136,139,140,141]. In the first case, the relationships between *G* and *R* allow for obtaining a solidification map as shown in Figure 15a where the lines and hyperbola branches are described by the $\frac{G}{R}$ (that affects the structure morphology) and *G* × *R* (that affects the microstructure scale), respectively.

Through this graphical representation, the cellular fine microstructure and cellular dendritic structure, which characterized the MP center (MPC) and the MP boundaries (MPBs), respectively, can be predicted (Figure 15b) [139].

**Figure 16:**
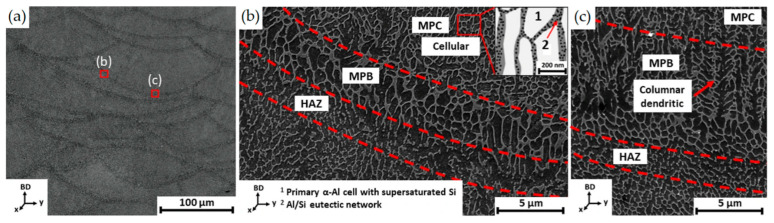
(**a**) SEM micrograph of the AlSi10Mg sample along the xz plane: (**b**,**c**) SEM micrographs at high magnification of the MPBs that highlight the Si-eutectic network destruction and the columnar grains (Reprinted from reference [139]).

In the second case, *G* and *R* are related to *SDAS* (Secondary Dendrite Arm Spacing) as follows:

(6) $$SDAS=k{\left(G\times R\right)}^{-n}$$

where the material constants *k* and *n* are 43.2 m(K/s)*^n^* and 0.324, respectively [140]. Despite this equation describing the cast alloy, different research study validates the same equation to describe the AlSi10Mg alloy manufactured via L-PBF process considering the SDAS as the cellular island of α-Al [136,139]. Moreover, in this case, if the MPC is characterized by a higher cooling rate (~10^5^ ÷ 10^6^ K/s, [9,136,139]) than the MPB, the adjacent solidified material is exposed to annealing temperatures that generated the HAZ as expressed in Section 2. These three different zones are characterized by an increment of the grain size due to the Si diffusion [7,14,136,139,142,143]. De facto, this local high-temperature exposure modifies the cellular microstructure, destroying the Si-eutectic network (Figure 16) [139,142].

Delahaye et al. [142] showed the reduction in Si-rich fraction from the MPC and MPB to the HAZ due to the Ostwald ripening phenomenon. All of this reflects on the HV microhardness, as is widely reported in the literature [9,137,144]. 

**Figure 17:**
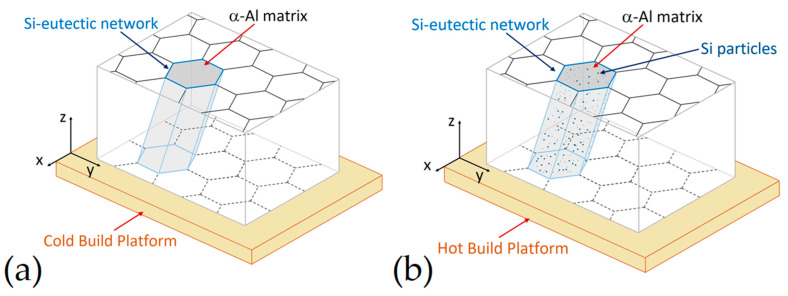
Schematic 3D rendering of the as build L-PBFed AlSi10Mg manufactured on: (**a**) cold BP, (**b**) hot BP.

From a three-dimensional point of view, as-built AlSi10Mg samples are characterized by a tubular structure of the α-Al matrix, which is surrounded by the Si-eutectic network (Figure 17a), due to the thermal gradient developed during the L-PBF [87,145]. In the same scenario, Figure 17b illustrates the same tubular structure containing, however, the Si particles that precipitate thanks to the pre-heated BP as previously discussed.

**Figure 18:**
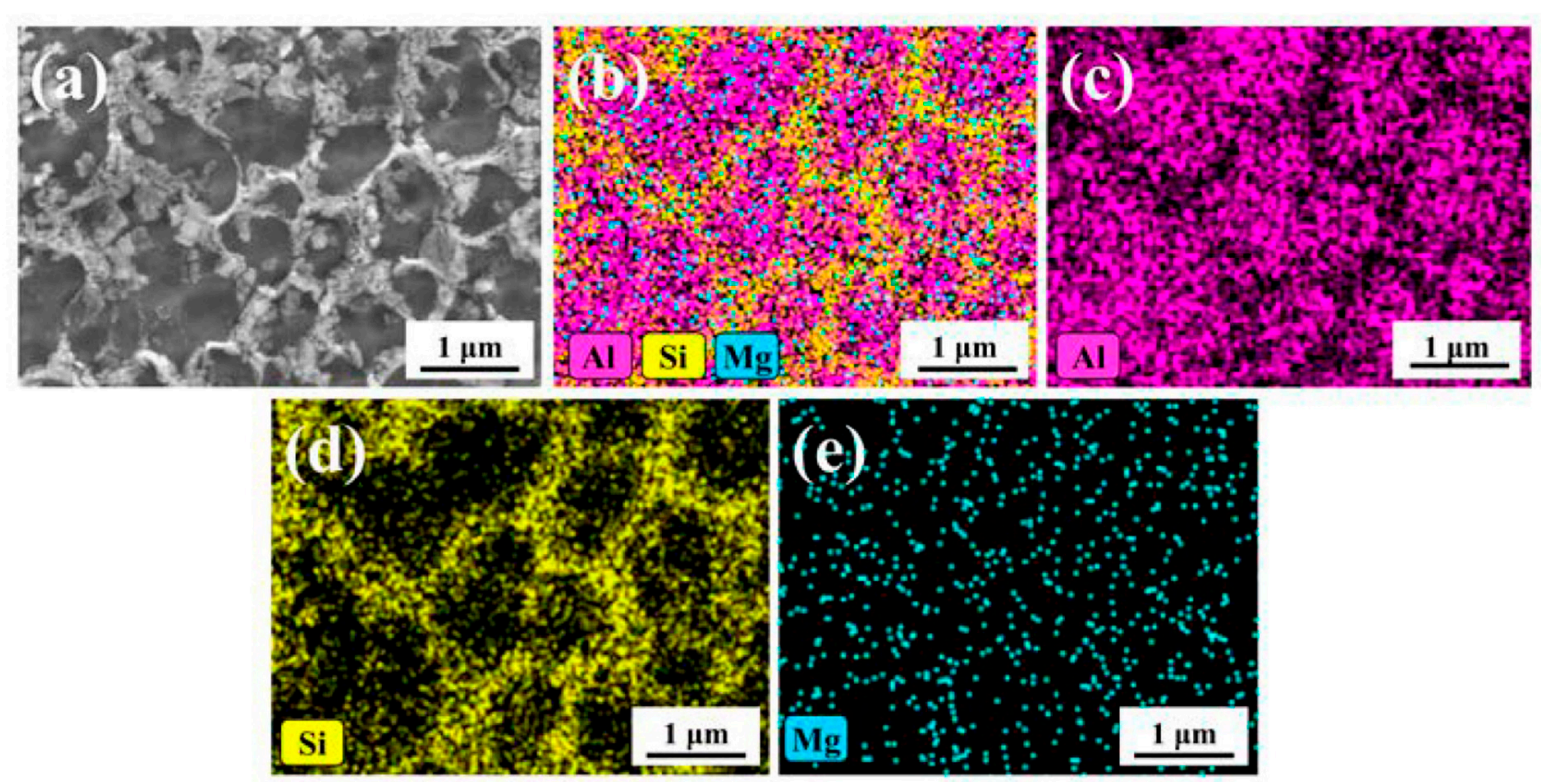
(**a**) Microstructural morphology of the as-built AlSi10Mg sample; (**b**–**e**) element mappings of the Al, Si and Mg (Reprinted from reference [146]).

As regards the Al, Si, Mg and Fe elements present in AlSi10Mg alloys, during the L-PBF process, these are distributed into microstructures (Figure 18a) as illustrated through the Figure 17b–e, as reported by Bai et al. [146]. The higher Si content is present in Si-eutectic particles along the cell boundaries where the Mg content is also segregated and into α-Al matrix (Figure 18b,d,e), as already discussed in Figure 14 and Figure 16. Zhou et al. [147] confirmed this distribution through the TEM measurements that also showed the (200), (111), (311) diffraction spots of Si-particles within the α-Al matrix cell boundaries. In addition, the same authors reported the presence of fine acicular Si precipitates (length of 50 ÷ 300 nm and width of ~10 nm) characterized by the following orientation relationship: ${\left[001\right]}_{\mathrm{Al}}∥{\left[12\bar{2}\right]}_{\mathrm{Si}}$ and ${\left(200\right)}_{\mathrm{Al}}∥{\left(111\right)}_{\mathrm{Si}}$. In conclusion, they did not report if the BP was used at room temperature or at higher temperatures.

**Figure 19:**
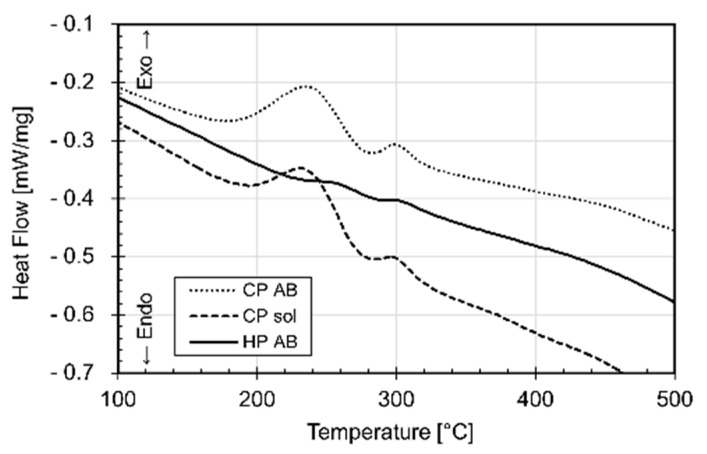
DSC analysis of as built AlSi10Mg samples manufactured using a cold BP at 35 °C (CP AB) and a pre-heated BP at 200 °C (HP AB). CP sol represents the CP AB sample after the solution heat treatment (SHT) (Reprinted from reference [138]).

Finally, Fe content is distributed into Fe-rich intermetallic phases such as π-Al_8_Si_6_Mg_3_Fe [147] or as brittle needle-like β-Al_5_FeSi phase [148,149]. At the same time, the AlSi10Mg is an age-hardening alloy (Figure 1a) that is characterized by the precipitation phenomena of the ε-Mg_2_Si phase [7,150,151]. Some authors show the presence of this ε phase already in as-built samples [14,148], while other studies do not achieve the same results [152,153]. As a matter of fact, Mathe et al. [154] showed an increase of ε phase with a decrease in the *ED* from 133 to 67 J/mm^3^. Casati et al. [138] showed, however, an increase in the precipitation phenomena using the pre-heated BP. De facto, the CP AB (cold platform at 35 °C, as-built) sample presents the sequence of precipitation peaks in the DSC (Differential Scanning Calorimetry) curves shown in Figure 19, unlike the HP AB (hot platform at 200 °C, as-built) sample, proving that the pre-heated BP at 200 °C induces precipitation phenomena of the ε-Mg_2_Si phase.

Cerri et al. [14], who analyzed the effects induced by the pre-heated BP at 150 °C, showed different amounts of ε-Mg_2_Si and Si particles within the α-Al matrix between the bottom and top regions into an AlSi10Mg billet (height of 300 mm). Thus, despite the use of a pre-heated BP, the as-built sample can be characterized by a different distribution of the precipitation phenomena induced by the BP temperature.

### 3.2. Heat-Treated Microstructure

Table 3 reports the HTs analyzed in the present review and the nomenclature used.

The direct aging (DA) HT allows researchers to strengthen the as-built AlSi10Mg through the precipitation phenomenon of the ε-Mg_2_Si phase, which follows the subsequent precipitation sequence: SSS of Al → GP zones formation (aggregation of Si/Mg atoms) → dissolution of Mg → cluster formation → precipitation of ε″ phase → precipitation of ε′ and ε″ phases → precipitation of stable ε-Mg_2_Si phase [7,150,155]. Different studies show the DSC curve performed on the as-built AlSi10Mg samples highlighting the exothermic peaks related to the precipitation phenomena of the ε-Mg_2_Si sequence. Fiocchi et al. [155] showed a single precipitation peak at 256 °C attributable to ε-Mg_2_Si phase performing the DSC between 0 and 500 °C. Van Cauwenbergh et al. [139] showed, instead, the first and second exothermic peaks at 195 and 295 °C, respectively, related to the precipitation phenomenon. Tonelli et al. [156] reported another exothermic peak at 150 °C related to the ε″ precipitate. In this scenario, these studies can confirm the effects induced by the pre-heated BP at temperatures between 100 and 200 °C.

The stress relief (SR) HT was often used to remove the manufactured samples from the BP to avoid their deformation.

Finally, T6 HT allows for obtaining an increase in ductility thanks to the solution heat treatment (SHT) and the alloy strengthening through the precipitation phenomena during the following artificial aging (AA). While HIP HT was used to increase the sample density.

**Figure 20:**
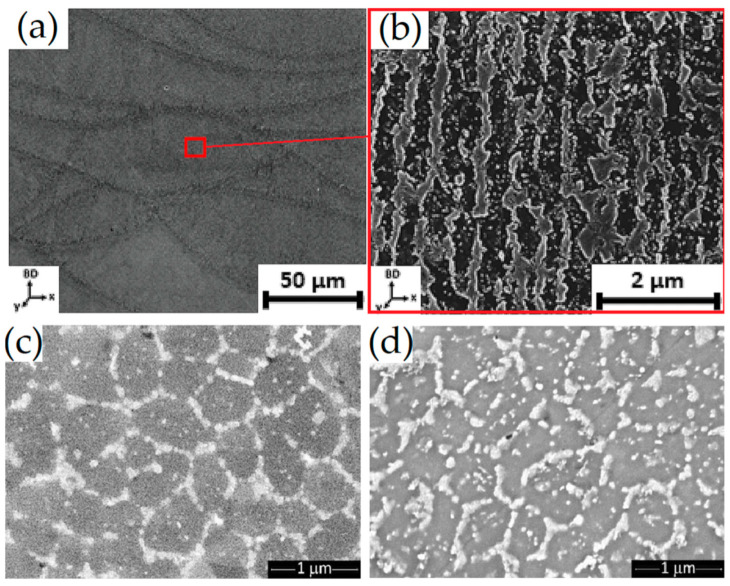
SEM micrographs of the AlSi10Mg samples DA at: (**a**,**b**) 170 °C × 6 h; (**c**) 200 °C × 6 h; (**d**) 225 °C × 6 h (Adapted from references [14,139]).

The microstructures obtained after the DA (180 ÷ 225 °C) are shown in Figure 20, as reported by [14,139].

**Figure 21:**
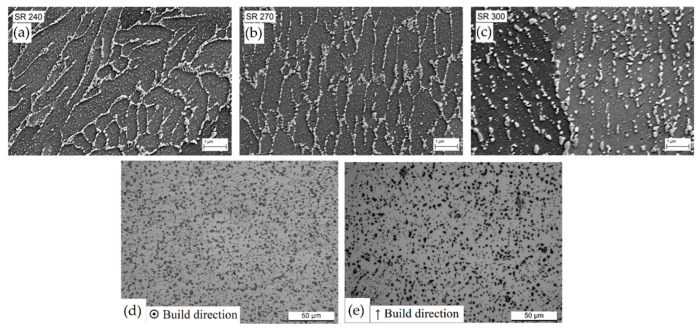
SEM micrographs: (**a**–**c**) of AlSi10Mg samples SR at 240 (**a**), 270 (**b**) and 300 °C (**c**), respectively; OM micrographs of the T6 heat-treated performed along the: (**d**) xy plane, (**e**) xz plane (Adapted from references [9,139]).

At low magnification (Figure 20a), the MPC and MPBs do not show any microstructural variation, as also reported by [9,157,158,169]. On the other hand, finer Si-rich precipitates are visible in the α-Al matrix, as shown in Figure 20b by [139]. It is necessary to underline that the sample analyzed in Figure 20a,b was manufactured on the BP at room temperature (Figure 13). The same authors analyzed the AlSi10Mg alloy in the same condition shown in Figure 13, where the samples were manufactured on the BP at room temperature. Cerri et al. [14] showed an increase in these precipitates, which were already present in as-built α-Al matrix, after the DA at 200 °C for 6 h (Figure 20c) and at 225 °C × 6 h (Figure 20d). At the same temperature, Baek et al. [157] affirmed that the precipitation of these particles can cause a uniform distribution of the dislocation, unlike the HT performed at 225 °C, which induces initial destruction of the Si-eutectic network. The openings within this network become larger as the temperature increases from 240 to 300 °C during the SR HT due to the Ostwald ripening effect (Figure 21a–c) [139,155,159,160,163,164].

Moreover, in this case, Van Cauwenbergh et al. [139] did not detect the presence of the stable ε-Mg_2_Si phase even after the SR HT at 270 °C, confirming the DSC analysis previously discussed.

Despite the loss in mechanical properties (that will be discussed in Section 4), SR heat treatment aims firstly to decrease the residual stress that is generated during the L-PBF process and, secondly, to avoid the consequent deformations derived by the removal of the printed sample from the BP [156,161]. In this scenario, the use of the pre-heated BP can prevent the execution of the SR HT due to the lower amount of residual stress into the as-built sample [138,162]. At the same time, the Si-eutectic network is not destroyed (Figure 13) and the mechanical properties are preserved (which will be discussed in Section 4).

**Figure 22:**
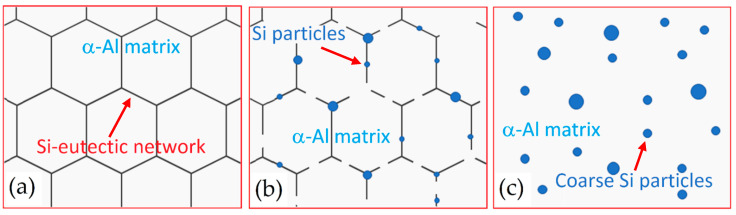
Schematic representation of the Si-eutectic network evolution during the SHT treatment, where the α-Al matrix is the gray background: (**a**) as-built AlSi10Mg with a full cellular structure where the Si-eutectic network is undamaged, (**b**) Si-eutectic network destroyed with the initial Si-particle coarsening, (**c**) Si-particle coarsened.

Figure 21d,e show the AlSi10Mg microstructure after the T6 heat treatment (SHT + AA), where the high anisotropy characterizing the as-built sample is cancelled even if, in Figure 21d, the MPBs can still be observed after the SHT at 505 °C × 4 h [9]. Ji et al. [170] explained this behavior through the Si content variation between the laser scan tracks boundaries and their centre. During the SHT, the microstructural evolution follows the schematic representation shown in Figure 22.

**Figure 23:**
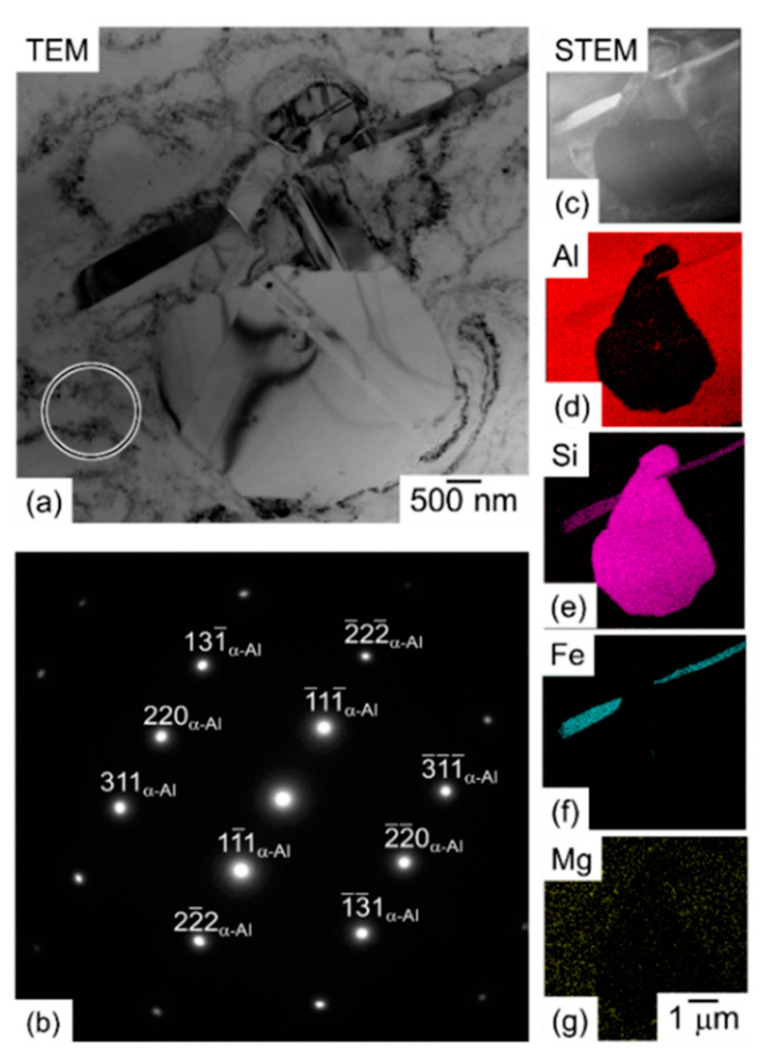
(**a**,**c**–**g**) STEM-HAADF (Scanning Transmission Electron Microscope—High-Angle Annular Dark Field)images of the β-Al_5_FeSi intermetallic phase, Al, Si and Mg elements detected into solution heat treated AlSi10Mg sample at 530 °C × 6 h. (**b**) SAED (Selected Area Electron Diffraction) pattern of the area marked with a circle in (**a**) (Reprinted from reference [173]).

After the SHT + AA, the Si is rejected from the α-Al matrix and forms small Si particles as illustrated in Figure 22a,b. Due to the high SHT temperature/time, the Si particles precipitate along the Al-Si cellular boundaries and grow up as reported in Figure 22c. By increasing the Si particle size, their density in terms of quantity decreases. The increase in size with the SHT temperature is related to the decrease of Si density; the same authors confirm that the as-built α-Al matrix is SSS. De facto, the excess of Si is rejected from the lattice structure [170,171]. Other authors showed an increase in Si particle size also with the holding time at high SHT temperature [148,172]. In this scenario, Li et al. [171] reported the Si solubility study considering the following Vegard’s law:

(7) $$\varpi =-0.0032X_{si}+0.40494$$

where ϖ is the lattice parameter of α-Al and *X_Si_* is the atomic fraction of Si into α-Al. The same authors show a solubility of 8.89% for the as-built AlSi10Mg and 3.25, 2.75 and 2.13% after the SHT at 450, 500 and 550 °C, respectively. The values were reduced by the following AA at 180 °C for 12 h to 2.52, 2.02, and 1.68%, respectively, due to the precipitation phenomena. During the same SHT, Zhou et al. [147] also showed the presence of needle-like Fe-rich phase (β-Al_5_FeSi), in addition to the spherical precipitates rich in Si and Fe within the α-Al. The same authors confirm the precipitation of needle-like ε″ precipitates (length < 10 nm) placed along <100> direction and GP zones after 520 °C × 2 h of SHT and 160 °C/2 h of AA. Liu et al. [173] showed the same results after 530 °C × 6 h, as shown in Figure 23.

They also affirmed the absence of ε′ and ε precipitates, unlike Wei et al. [172], who confirmed the presence of ε-Mg_2_Si precipitate after 540 °C × 2 h.

The same results were obtained by Iturrioz et al. [174], who analyzed the AlSi10Mg samples after SHT at 450 and 550 °C and AA at 180 °C. They supposed that the undetectability of the ε-Mg_2_Si precipitates is because their lower amount than the detection limit. On the other hand, the intensity of Si peaks increases from the as-built to the heat-treated condition confirm the increase of Si content into α-Al due to the Si precipitates as reported by [14,143,147,170,174]. In addition, the T6 HT induces a coarsening of the columnar grains formed during the L-PBF fusion rather than the DA and SR HTs [157]. Increasing the SHT temperature and/or time, the recrystallized equiaxed grains form due to the recrystallization process [148]. This phenomenon balances the reduction in plasticity induced by the Si particles precipitation [143]. Wei et al. [172] showed that the grain size increases slightly between the as-built- and T6 heat-treated at (540 °C × 2 h) + (170 °C × 4 h) samples and that the % of recrystallization grains is the same. De facto, Chen et al. [175] affirmed that the dislocations present within the cellular boundaries of the as-built sample can act as nucleation sites. On the other hand, Si particles can hinder the grain growth because of the recrystallization process during the T6 HT [172,175]. If the higher microstructural variation takes place during only the SHT treatments, Merino et al. [165] showed Si particles coarsening, even after an AA performed at 177 °C × 1000 h.

In conclusion, the HIP HT, which can be used to reduce the internal pores inducing the sample’s densification, confers the same microstructural effects of the T6 HTs [107,108,167]. Merino et al. [165] showed a complete recrystallization process after HIP (515 °C × 3 h × 100 MPa) and HIP + T6 (515 °C × 3 h × 100 MPa) + ((530 °C × 6 h) + (160 °C × 6 h)) HTs. In this scenario, Ertuğrul et al. [107] also combined the HIP HT with the T6 to increase the mechanical properties, but the round Si particles become larger and more spherical, and the microstructure shows needle-like Fe-rich phases. The same results were reported by Schneller et al. [108] and Hafenstein et al. [167] who showed a decrease between 64 and 66% of the internal pores.

## 4. L-PBFed AlSi10Mg: Mechanical Properties

Table 4 shows the mechanical properties of L-PBFed AlSi10Mg samples before and after the heat treatments of which microstructural effects are discussed in Section 3.2. (DA, SR, SHT, and HIP). Due to the influence of the *ED* on both the presence of defects and microstructure, all studies reported in the following table showed *ED* values from 35 to 60 J/mm^3^. Thus, all illustrated values are comparable to each other. For a better understanding, H and V represent the horizontal and vertical directions, respectively.

Generally, the tensile strengths of the as-built samples reach high values due to the microstructure shown in Figure 12, Figure 13 and Figure 20. On the other hand, the ductility values do not meet the standard specification and project requirements very often (Table 4) [9,13,137,139,166,176]. In this scenario, despite the similar *ED* values of all studies analyzed, the process parameters and the build chamber orientation significantly influence the mechanical properties of the as-built sample [137,138,143,176,177]. Firstly, Paul et al. [137] showed a reduction in the strength of ~10–12% with an increase in t from 30 to 60 μm. In this case, the ductility was not affected. The UTS (Ultimate Tensile Strength) and YS (Yield Strength) reach 323 MPa and 190 MPa from 367 MPa and 244 MPa, respectively, with 190 μm of hatch spacing. Both the UTS and YS values increase with decreasing the hatch spacing at 100 μm, as shown in Table 4. Ghio et al. [9] showed, instead, increase in strengths of about 5.5% by increasing the layer thickness (+40 μm) and decreasing the hatch spacing (–100 μm). Secondly, the H-samples show lower UTS and YS than the V-samples, in addition to the ductility’s variation, highlighting the anisotropy that characterizes the as-built samples [138,139,143]. Other authors have not reported differences between the tensile strength values in relation to the build orientation [177,178]. In terms of ductility, Ben et al. [177] explained this variation, firstly, through the load conditions during the tensile test, secondly, through the presence of voids. They affirmed that the crack-like voids (LOF) present along the MPBs are more dangerous than the spherical pores (Section 2) due to their different deformation during the tensile test. The spherical pores show limited deformation compared to the crack-like voids along the load direction.

Another factor that influences the tensile properties is the presence of the pre-heated BP during the L-PBF process, which influences the precipitation phenomena, as reported in Figure 19. Cerri et al. [14] reported a decrease in UTS and YS from 430 ± 8 MPa and 365 ± 7 MPa and from 286 ± 8 MPa to 220 ± 2 MPa, respectively, analyzing the effects of the pre-heated BP at 150 °C (see both Table 4 and Section 3.1). As a matter of fact, the aging phenomenon and Si-particles precipitation occurring in as-built samples (Figure 13) increase the tensile strengths. The elongation values are not significantly influenced. At the same time, the BP effects are equally significant after the DA HT performed at similar temperatures of the platform. De facto, Cerri et al. [14] showed an increase of UTS and YS values on top samples (which are not affected by the hot BP) rather than the bottom samples after the DA at 175 °C × 6 h. Yang et al. [13] highlighted the same effects performing the DA at 160 °C × 8 h on AlSi10Mg samples manufactured on cold BP (35 °C), while Casati et al. [138] showed these effects after the DA at 160 °C × 4 h. Finally, it is necessary to observe that the effects induced by the pre-heated BP are strictly related to its temperature, and to the printing time.

Analyzing the SR HT, no study shows the increase in the tensile strengths due to the microstructure reported in Figure 21. Obviously, the higher decrease in strengths is obtained only after the SHT or HIP HTs when the UTS and YS values are similar to those obtained for the as-cast AlSi10Mg alloy [7,10,49,147]. On the other hand, the elongation values reached with the SR heat treatment can already satisfy the standard specification requirements (A > 10%) [166]; however, those obtained after the SHT and HIP heat treatment reach the maximum obtainable values (23 ÷ 31%). In all cases, the tensile strengths were recovered through opportune DA heat treatments. Li et al. [176] showed the UTS and YS increase of 30 and 50%, respectively, after the DA at 160 °C × 2 h performed on solution heat-treated samples at 500 °C × 2 h. The same results are reported by [143,170]. For each heat treatment, a disproportionate holding time at the aging temperatures induces a decrease in strength due to the microstructural effects, as shown by Merino et al. [165]. The same authors applied different DA heat treatments at 177 °C × 10, 100, 1000 h on HIPed and T6 heat-treated.

**Figure 24:**
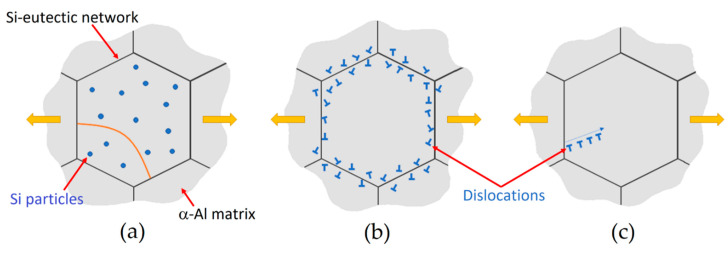
Schematic representation of plastic deformation mechanisms of the full cellular structure: (**a**) dislocation de-pinning from the atoms dispersed into SSS α-Al matrix; (**b**) deformation by cutting dislocation “forests”; (**c**) emission of dislocation from the interface between the Al and Si.

The decreasing trend of UTS and YS values, opposite to the elongation values, are appropriately described by [157,164,165,176]. Starting from the as-built samples and the direct aged samples at low temperature (where no microstructural variation was observed), Li et al. [176] proposed three deformation scenarios (Figure 24) that deviated from the Orowan bowing mechanisms around the Si particles and that could justify the tensile strength behavior. The first one is the dislocation de-pinning from supersaturated atoms in α-Al (Figure 24a); the second one is the deformation by cutting dislocation forest near the interface between the Al/Si interfaces (Figure 24b); the last one is the dislocation emission from the same interfaces (Figure 24c).

The presence of dislocation density around the solute atoms into the aluminium matrix is also reported in Section 3.1.

**Figure 25:**
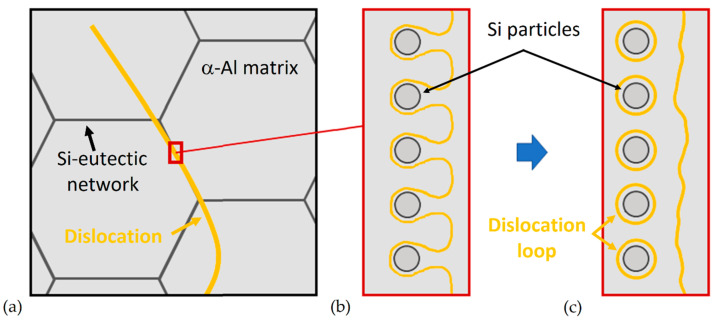
Schematic representation of the dislocation interaction (**a**,**b**) with the cell boundaries via Orowan mechanism and formation of the dislocation loop (**c**).

Thus, the high mechanical properties can be expressed through the following sum of strengthening effects [182,183]:

(8) $$\sigma_{0.2}=\sigma_{f}+\sigma_{ss}+\sigma_{HP}+\sigma_{Or}+\sigma_{P}+\sigma_{pre}$$

where $\sigma_{f}$ (MPa) is the friction stress of the lattice, $\sigma_{ss}$ (MPa) is the solid solution strengthening, $\sigma_{HP}$ (MPa) is the strength obtained by the grain size (Hall–Petch equation), $\sigma_{Or}$ (MPa) is the Orowan strengthening, $\sigma_{p}$ (MPa) is the dislocation hardening and $\sigma_{pre}$ (MPa) is the contributed sum of the dislocation and precipitates. Li et al. [176] showed that the first parameter can be calculated as 5.5 MPa; for this reason, it can be neglected if compared with the other contributions. Yang et al. [13] expressed the solid solution strengthening as follows:

(9) $$\sigma_{ss}=\left[k_{Mg}{\left(C_{\alpha }^{Mg}\right)}^{m}+K_{Si}{\left(C_{\alpha }^{Si}\right)}^{m}\right]$$

where $k_{Mg}$ and $k_{Si}$ are 17 and 11 MPa wt%^−1^, respectively, m is 1 and C is the chemical concentration of Mg and Si into the α-Al matrix, respectively. The same authors suggest that this contribution is negligible after the T6 HT. Hadadzadeh et al. [182] proposed the use of the Hall–Petch equation (considering the cellular structure of the L-PBFed AlSi10Mg):

(10) $$\sigma_{HP}=\frac{K}{\sqrt{d}},$$

where K is a material constant (~0.04 MPa m^1/2^, [184]), and d is the average cell size (m). The Orowan strengthening was expressed by Dieter [185], as follows:

(11) $$\sigma_{Or}=\frac{0.13Gb}{\lambda }\ln\frac{r}{b},$$

where G (GPa) is the Al shear modulus (26.5, [158,183]), b is the Burgers vector (0.286 nm), [158,184], λ is the interparticle spacing (nm) $\left(\lambda =r{\left(\frac{2\pi }{3f}\right)}^{\frac{1}{2}}\right)$ [148], and r is the particle radius (nm). Figure 25a,b show the schematic interaction between a dislocation and the cell boundaries via Orowan looping in a full-cellular AlSi10Mg structure [164]. When an applied load moves a large dislocation (same magnitude of the grain size) into the AlSi10Mg microstructure, it will be pinned by the Si-eutectic network, forming both dislocation loops around the Si-particles and a new dislocation (Figure 24a and Figure 25b). Chen et al. [186] observed a high amount of dislocation and dislocation loop through HRTEM (High-Resolution Transmission Electron Microscope) observations in a deformed AlSi10Mg sample.

Rodriguez [187] expressed the dislocation hardening contribution as follows:

(12) $$\sigma_{p}=\beta MGb\sqrt{\rho_{p}}$$

where β is a material constant (0.16, [181]), M is the Taylor factor (3.06, [158,184]), G (GPa) is the Al shear modulus, b is the Burgers vector and $\rho_{p}$ is the density dislocation. Finally, Starink et al. [188] suggested the following equation to predict the strengthening contribution conferred by the dislocation and precipitates:

(13) $$\sigma_{pre}=C_{4}\frac{Gb}{\sqrt{l_{D}l_{t}}}\left[\sqrt{f}+0.7f\sqrt{\frac{l_{D}}{l_{t}}}+0.12\left(\frac{l_{D}}{l_{t}}\right)\sqrt{f^{3}}\right]$$

where $C_{4}$ (-) is a material constant, $l_{D}$ and $l_{t}$ are the diameter (nm) and thickness of the precipitate (nm), f is the volume fraction (-). Thus, considering all contributions expressed through the Equations (9)–(13), the Equation (8) can be rewritten as follows:

(14) $$\sigma_{0.2}=5.5+\left[k_{Mg}{\left(C_{\alpha }^{Mg}\right)}^{m}+K_{Si}{\left(C_{\alpha }^{Si}\right)}^{m}\right]+\left[\frac{K}{\sqrt{d}}\right]+\left[\frac{0.13G_{m}b}{\lambda }\ln\frac{r}{b}\right]+\left[\beta MGb\sqrt{\rho_{p}}\right]\;+\left[C_{4}\frac{Gb}{\sqrt{l_{D}l_{t}}}\left[\sqrt{f}+0.7f\sqrt{\frac{l_{D}}{l_{t}}}+0.12\left(\frac{l_{D}}{l_{t}}\right)\sqrt{f^{3}}\right]\right]$$

Focusing on the microstructure obtained after the T6 and HIP HTs (Section 3.2), some contributions reported in Equation (13) decrease with respect to the as-built case. Baek et al. [157] showed a decrease of $\sigma_{HP}$ from 89.22 MPa to 12.46 MPa considering the as-built and T6 heat-treated sample, respectively. The same authors highlighted an obvious increment of the Orowan strengthening from 59.05 MPa (as-built condition) to 183.30 MPa considering the direct aged samples (180 °C × 6 h), and a subsequent drastic decrease to 12.56 MPa analyzing the T6 heat-treated samples. Finally, the contribution related to the dislocation amount decreases with the HT temperature and holding time (Section 3.2). Merino et al. [165] observed that the mechanical properties of T6 and HIP heat-treated samples is dominated by the grain size and Si particles in terms of size and distribution. In fact, the tensile strength decrease showed by the same authors is confirmed through the coarsening effects (Section 3.2), and not through the precipitation phenomena, despite the DA HT. The same observations were emphasized by Baek et al. [157]. Wei et al. [172] did not show the same results due to the slight increase in the grain size after the T6 HT. They affirmed that the decrease in hardness is dominated only by the reduction of the dislocation amount within the cells and not by the grain size.

**Figure 26:**
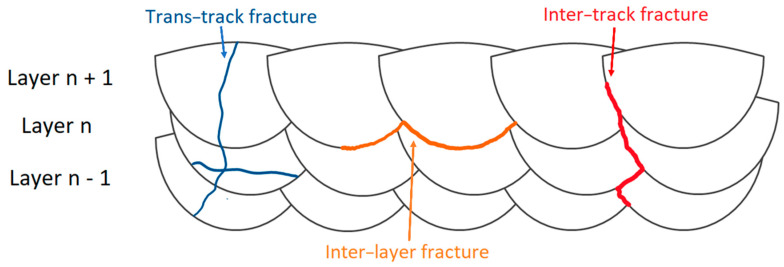
Fracture mechanism of L-PBFed AlSi10Mg samples.

In this scenario, the microstructural configuration reported in Figure 11, Figure 16 and Figure 21 dominate the fracture mechanisms and the elongation of the L-PBFed AlSi10Mg samples in as-built and heat-treated conditions. Delahaye et al. [142], who analyzed the fracture mechanism into as-built AlSi10Mg samples, highlighted that the dislocation can easily move into the HAZ zones rather than into MPC due to their microstructure (Figure 16) and due to the different values of the yield strength. Other important factors characterizing the fracture mechanism are the Si-particle decohesion from the α-Al matrix, and the formation of voids at their interface [142,179]. This crack path characterizes more the V-samples than the H-samples, as reported in different studies [137,177,189,190]. De facto, the load direction changes the mechanical behavior between the H- and V-samples due to the microstructural texture characterizing the as-built samples [13,137]. Yang et al. [13] affirmed that the H-samples show a grain deformation, while the V-samples are characterized by deformation along the MPBs through a detailed description of the strain and stress anisotropy. Thus, the path of the cracks can vary, as schematically shown in Figure 26 and carefully described by [137,189].

The presence of internal pores is another important factor into damage mechanisms because they caused an early break of the sample under a load [13,177,190].

**Figure 27:**
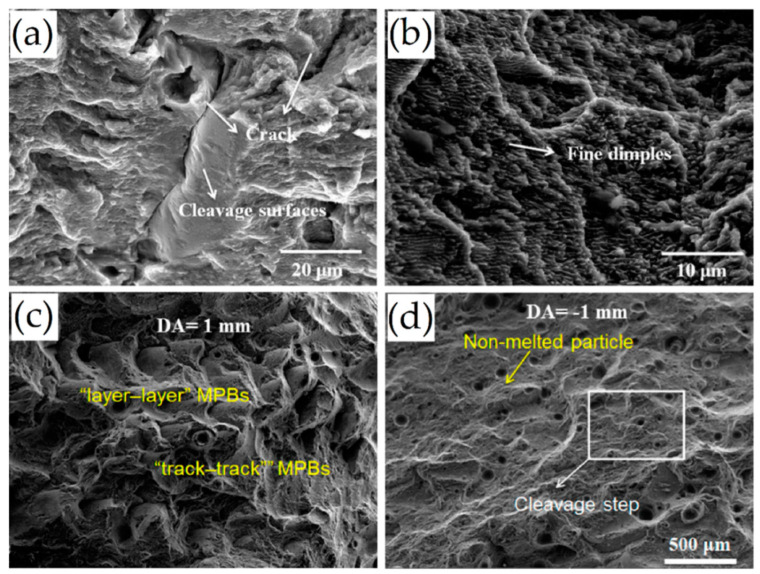
Fracture surfaces of the as-built AlSi10Mg H-samples after tensile test that show: (**a**) crack, (**b**) mixture of small dimples, (**c**) layer-layer MPBs and track–track MPBs crack pahts, (**d**) non-melted powder and cleavage step (Adapted from reference [191]).

**Figure 28:**
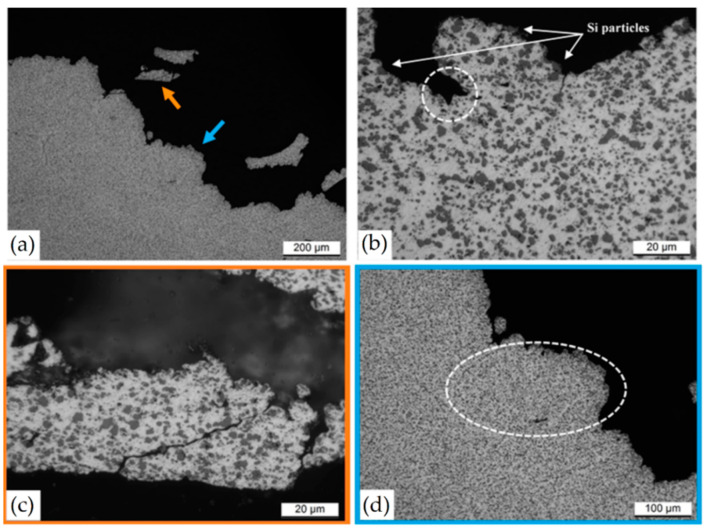
Fracture profiles (**a**,**b**) of the T6 heat-treated AlSi10Mg samples at (505 °C × 4 h) + (175 °C × 4 h) where (**c**,**d**) highlight the zones indicated by the orange and light-blue arrows, respectively (Reprinted from reference [9]).

The fracture surfaces shown in Figure 27, analyzed by Zhou et al. [191], report the presence of dimples, cleavage surface, cracks and the typical geometry of the MPBs, which confirms what was previously discussed. The lamellar features and the segments of the Si-eutectic network present on fracture surfaces are caused by the load-bearing capacity of the Si network and by the load transfer from the same particles to the α-Al matrix [179]. The same authors proposed an interesting scheme of damage mechanisms about the microstructural variation from the full-cellular to coarsened structures. In the former case, the crack propagates along the cell boundaries, while, in the latter one, the crack interconnects the Si particles and voids caused by the Si-particle decohesion from the matrix. Figure 28 shows what was just reported, in addition to the effects induced by the MP boundary that remained in the T6 heat-treated microstructure (Section 3.2) [9]. The same results are shown in other studies conducted by [9,13,107,142,157]. Martin [192] proposed the following correlation to obtain the work W necessary to create a crack between the Si-particle and the α-Al matrix:

(15) $$W\propto \gamma_{Al}+\gamma_{Si}-\gamma_{AlSi}$$

where $\gamma_{Al}$ (J/m^2^) and $\gamma_{Al}$ (J/m^2^) are the matrix and precipitate surface energy, while $\gamma_{AlSi}$ (J/m^2^) is the interface energy. This last term is strictly related to the Si-particle size, and it increases with the size.

The coarsened microstructure obtained after the T6 or HIP HTs increase the value of the interface energy ($\gamma_{AlSi}$) and, consequently, reduces the work necessary to nucleate a crack. In addition, the nucleation and propagation of the crack can be influenced by the presence of brittle β-Al_5_FeSi phases and by the density variation after the HTs [13,49,107]. As a matter of fact, already after the SR at 300 °C × 2 h the sample’s density can decrease from 2.68 g/cm^3^ to 2.58 ÷ 2.61 g/cm^3^ as reported by Mfusi et al. [160]. The same results were also obtained after different T6 heat treatments by [13,48,49]. Yang et al. [13] and Girelli et al. [49] justified the increase in porosity through the matrix deformation caused by the gas pressure during the heat treatment. De facto, the yield strength of the material around the pore decreases due to the high temperature; the increase in gas pressure can deform it.

## 5. L-PBFed AlSi10Mg: The Corrosion Resistance

The corrosion resistance of the as-built and heat-treated AlSi10Mg samples is strictly related not only to their surface finishing but also to their microstructure, which depends on the following aspects: *ED* values used during the L-PBF process, HT time and temperatures, build orientations and presence of defects (Table 5) [193,194,195,196,197,198,199,200].

**Figure 29:**
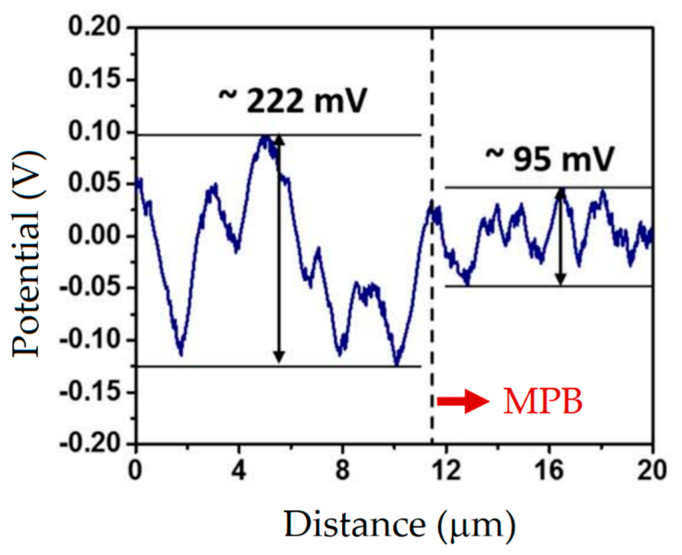
Potential profile along the HAZ and MPB of the as built AlSi10Mg (Adapted from reference [201]).

Fathi et al. [198] showed an apparently better corrosion resistance of the as-built L-PBFed AlSi10Mg samples than the same as-cast samples due to their finer microstructure. Revilla et al. [197] demonstrated that the potential difference between the Si crystals inside the MPBs and the α-Al matrix was higher, at about 127 mV, than the same difference between the Si crystals and the outside zone (95 mV) (see Figure 29a). The same authors showed a presence of crystallographic pitting developing in the α-Al grains within the MPBs.

The same results were also obtained by [172,197,198,202]. Cabrini et al. [202] highlighted that the Si-eutectic network partially shielded the MPC from the corrosion attack because the local acidification formed during the corrosion process prevent the oxide film reformation.

**Figure 30:**
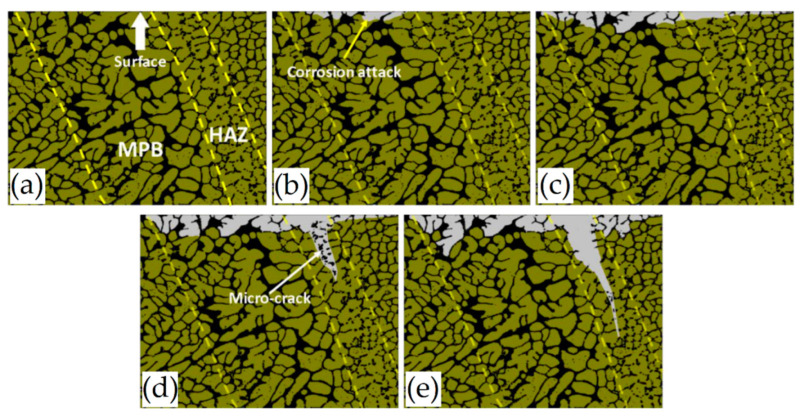
Schematic representation of the corrosion mechanism in the AlSi10Mg as built sample: (**a**) surface without corrosion attack, (**b**,**c**) initiation of the corrosion attack, (**d**) micro-crack formation, (**e**) micro-crack and corrosion attack propagation (Reprinted from reference [201]).

On the other hand, several studies show a large corrosion attack penetration along the MP boundaries [193,194,196,203]. De facto, Figure 30 shows a schematic representation of the corrosion initiation and propagation of the as-built L-PBFed Al-Si alloys as proposed by [204].

Figure 30a–c show the initiation of the corrosion attack that takes place at the MPB due to the higher driving force for galvanic corrosion induced by the higher potential difference. Moreover, crack formation can be due to the Si-eutectic destruction into HAZ correlated to the residual stress characterizing the as-built samples (Section 2 and Section 3.2) and to the α-Al matrix dissolution. Finally, the corrosion path can follow the crack propagation (Figure 30d,e) [203].

Finally, the presence of pores might play a significant role in stress corrosion cracking (SCC) due to their concentration point effects during the mechanical loading (Section 2.1 and Section 3). In this scenario, the residual stress characterizing the L-PBFed sample also influences the SCC [197].

As for the mechanical properties (Section 3.1), the corrosion resistance is also anisotropic along the xy and xz planes of the as-built samples [193,205]. It is, however, necessary to highlight that the greater anisotropy is obtained with the intergranular corrosion test [203] rather than with the potential dynamic polarization experiment in Harrison’s solution in the aerated solution of NaCl [193,204,205,206,207]. If Chen et al. [207] show higher corrosion resistance on the xz plane than on the xy for the as-built Al-12Si alloy, the previous studies show the opposite. This difference behavior can be attributable to the different chemical compositions, even if they belong to the same family.

**Figure 31:**
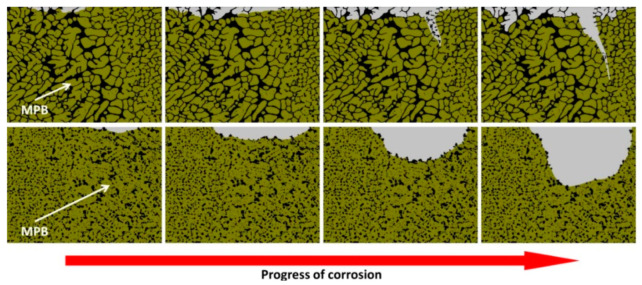
Schematic representation of the corrosion mechanism in the as-built AlSi10Mg (**first row**) and in the SR (**second row**) samples (Reprinted from reference [201]).

Starting from the DA samples, the corrosion attack takes place still along the MPB due to the slight microstructural change (Figure 20). Cabrini et al. [194] showed that the penetration of the corrosion attack along the MPB was higher in the AlSi10Mg after DA at 200 °C rather than in the SR samples at 300 °C. In all cases, the influence of the build orientation remains visible. Rubben et al. [206] showed the corrosion attack along the MPB even after the SR at 300 °C × 2 h. As a matter of fact, the corrosion mechanism changes in relation to the heat treatment, as shown in Figure 31, due to the microstructural variation between the as-built (first row) and heat-treated samples (T > 300 °C, second row). Despite this, in both cases, corrosion propagation occurs along the MPBs.

Increasing the heat treatment temperature, Cabrini et al. [195] showed more general corrosion after the intergranular test performed on the heat-treated samples at 400 °C. De facto, the propagation of the corrosion attack is no longer obstructed by the Si-eutectic network that is gradually destroyed with the temperature (Figure 20 and Figure 21). Reaching the SHT temperatures and focusing on T6 HT, Wei et al. [172] reported an increase in the weight loss from 0 to 400 mg/cm^−3^ with the SHT temperatures and time.

On the corroded surface, the same authors showed the typical pit of pitting corrosion after the T6 HT, in addition to a greater corrosion effect around the Si particles and/or ε-Mg_2_Si precipitates after the corrosion test in 1M HNO_3_ solution. The dissolution of the α-Al matrix occurs for the following reasons after the T6 HT:

- The amount of the Si rejected from the α-Al;
- The formation of the ε-Mg_2_Si precipitates that increase the galvanic couple.

In addition, the corrosion effects accelerate if these precipitates are characterized by big dimensions [192]. Generally, the presence of the Mg alloying element within the chemical composition tends to decrease the corrosion resistance of the aluminium alloys; in fact, Al_12_Si is characterized by higher corrosion resistance than the AlSi10Mg and the AlSi7Mg0.6 [207,208,209,210]. De facto, the ε-Mg_2_Si phase is anodic to the α-Al matrix when the corrosion attack occurs, as demonstrated by Zeng et al. [208]. During the corrosion phenomenon, the Mg content decreases and the consequent shift of the potential to more positive values make the ε-Mg_2_Si cathodic to α-Al matrix. This variation in terms of potential is induced by the increase in the Si effects.

## 6. L-PBFed Ti6Al4V: Microstructure

### 6.1. As-Built Microstructure

**Figure 32:**
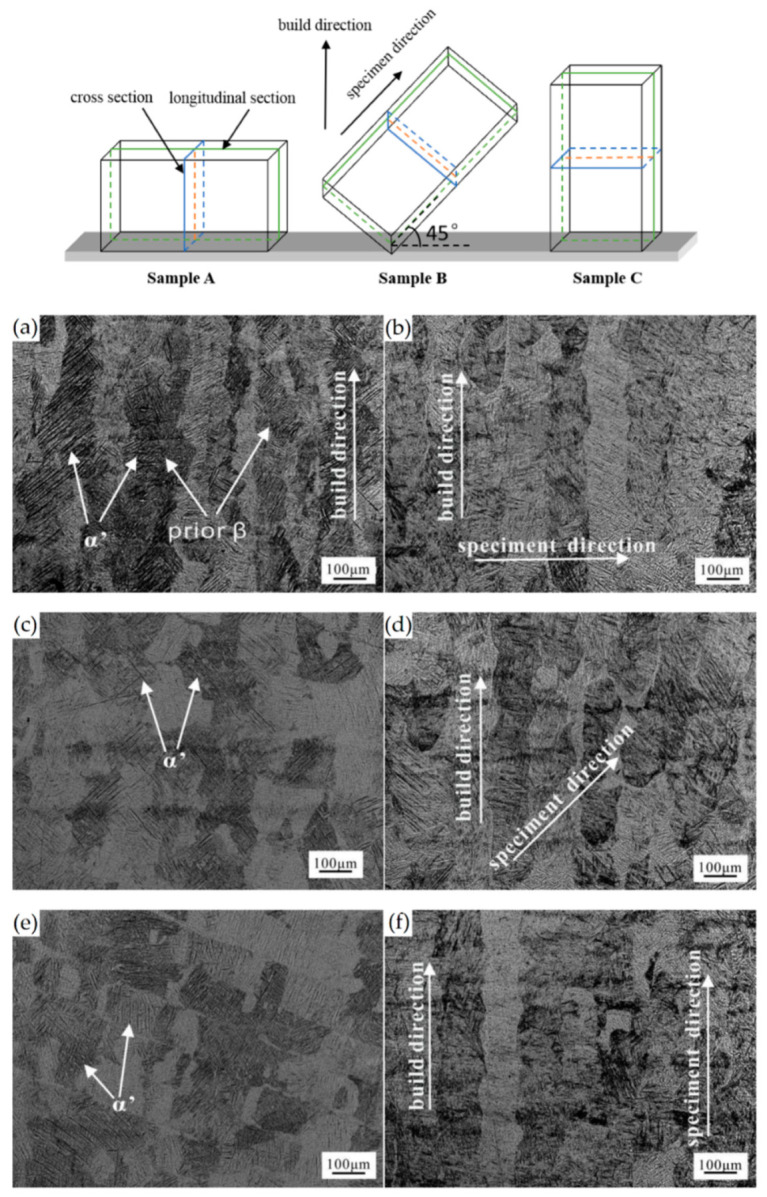
OM (Optical Microscope) micrographs of the as-built Ti6Al4V samples manufactured along different build directions: (**a**,**b**) H-sample, sample A; (**c**,**d**) 45°-sample, sample B; (**e**,**f**) V-sample, sample C (Adapted from reference [211]).

The as-built L-PBF-ed Ti6Al4V alloy shows a fully or majority martensitic (α′) microstructure within prior columnar β-grains that are arranged along the build direction (Figure 32a,b,d,f), as also reported by [32,59,211,212].

The typical square shape of the columnar β-grains is shown in Figure 32e, which represents the cross-section of sample C; they are contained in laser scan tracks. For this reason, their distribution and size depend on the scanning strategy [211,213,214]. De facto, some authors showed that their thickness size is in agreement with the width of the laser scan tracks [59,213], while the α′ martensite thickness is between 0.5 and 3 μm [215,216]. The main causes of this grains size variation are correlated firstly with the selection of different process parameters, which vary the *ED* and the heat transfer, and, secondly, with the temperature of the BP. Cepeda-Jiménez et al. [217] showed a fine and weakly textured microstructure at *ED* < 37 J/mm^3^, which became strongly textured at *ED* higher than 37 J/mm^3^. Other studies illustrate the same results [218,219,220]. Table 6 reports the correlation between the *ED* values and the obtained microstructure in the L-PBFed as-built Ti6Al4V samples, as reported by Xu et al. [221].

**Figure 33:**
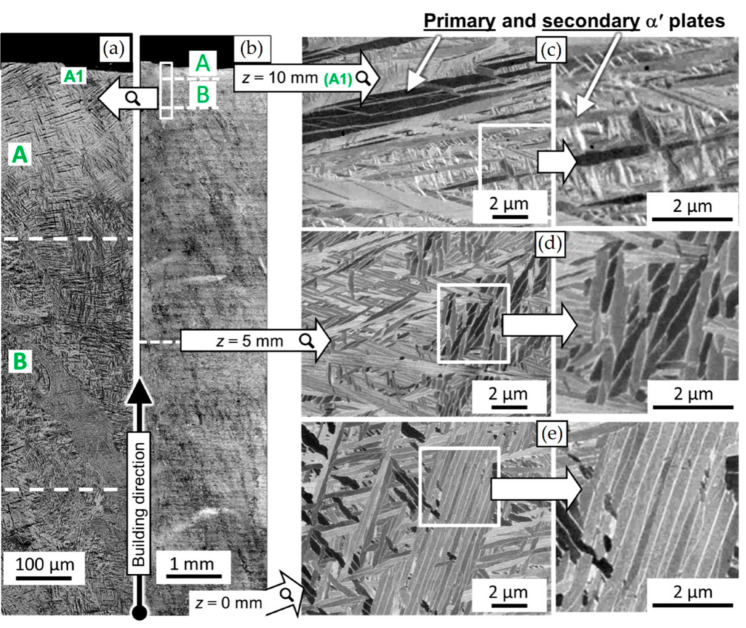
(**a**,**b**) OM micrographs performed along the *z*-axis, between top (A, A1) and bottom (B) regions; (**c**–**e**) SEM micrographs related to: (**c**) z = 10 mm, (**d**) z = 5 mm, (**e**) z = 0 mm (Reprinted from reference [222]).

Reducing the *ED* from 68.47 to 33.74 J/mm^3^ (increasing the layer thickness, hatch spacing and laser power from 30 μm, 120 μm and 175 W to 60 μm, 180 μm and 375 W, respectively), the fully acicular α′-martensite microstructure is gradually replaced by α + β lamellar structure. Barriobero-Vila et al. [222] showed a gradual microstructure transformation between the bottom region characterized by the ultrafine α + β microstructure and the top region showing the acicular α′-martensite (Figure 33). Xu et al. [221] suggested that the control of *ED* is necessary to achieve an ultrafine α + β during the L-PBF rather than after the STA (solution treatment and aging), as is discussed in Section 6.2.

**Figure 34:**
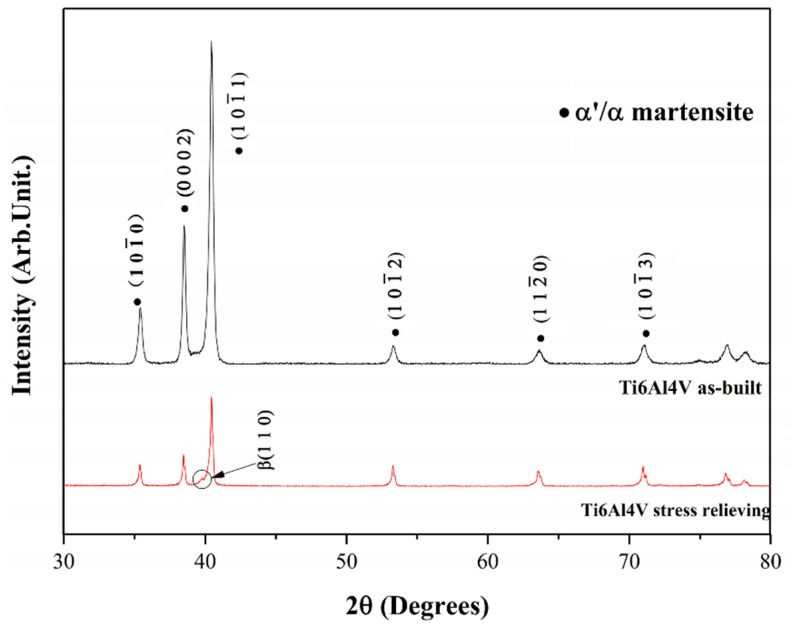
XRD spectra of as-built and stress relieved Ti6Al4V samples. In this context, the last heat-treated condition can be compared to an as-built sample also formed by α + β microstructure (Reprinted from reference [213]).

In this scenario, the as-built sample formed by a fully α′ martensite structure does not show any portion of the β-phase in the XRD spectrum, as shown in Figure 34. The same results are illustrated by [111,223,224,225]. On the other hand, increasing the presence of the α + β phase, the XRD spectrum starts showing small peaks related to the β-phase, as highlighted in the red spectrum related to the Ti6Al4V stress-relieving sample. These can be considered as confirmation of the α′ → α + β decomposition, as highlighted through the orange spectra (Figure 34) after the SR HT. Finally, the fully α + β microstructure increases the intensity of the peaks related to β and α phases, respectively.

**Figure 35:**
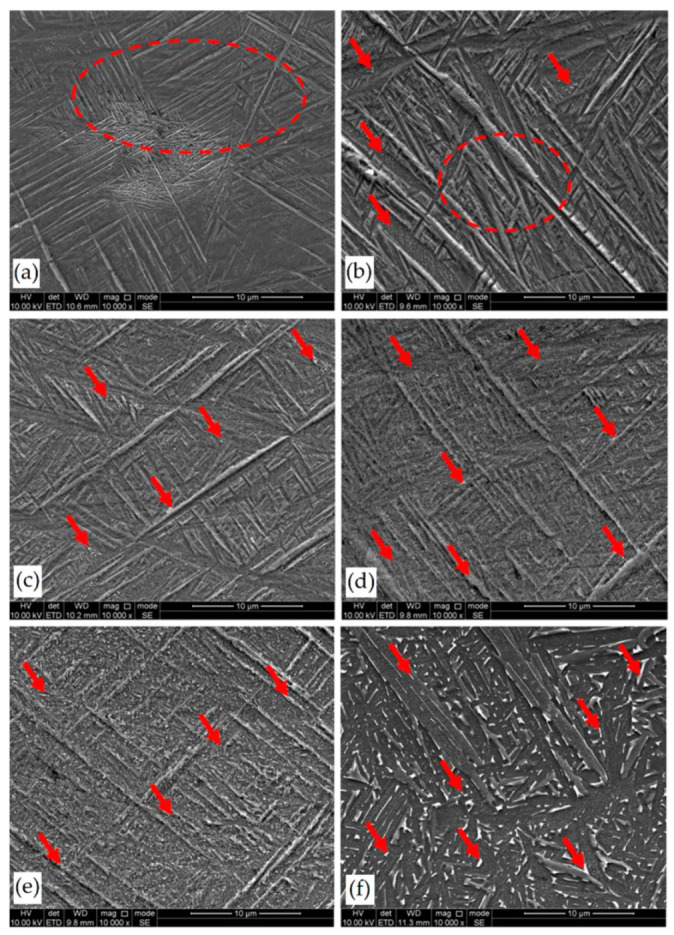
As-built Ti6Al4V microstructures of samples manufactured on BP at different temperatures: (**a**) 100 °C, (**b**) 370 °C, (**c**) 470 °C, (**d**) 570 °C, (**e**) 670 °C and (**f**) 770 °C where red arrows indicate the β-particles precipitate (**b**) and their growth (**c**–**f**) (Adapted from reference [226]).

Secondly, Ali et al. [226] demonstrated the effects induced on microstructures and mechanical properties by the pre-heated BP. At temperatures up to 370 °C, the microstructure remains martensitic but shows an increase in the α′ lath sizes with temperature (Figure 35a,b). At 470 °C, the α′ → α decomposition occurs according to the study on phase transformation of Ti6Al4V conducted by Kaschel et al. [227]. The authors reported TEM results indicating the decomposition temperature at 400 °C, as also shown by Xing et al. [228], and a full decomposition at 700 °C. Sallica-Leva et al. [229] reported, however, an exothermic peak related to the martensite decomposition between 760 and 850 °C. Other authors collocated this decomposition in the range of 600–800 °C [221,230]. Considering, instead, the BP at 570 °C, the α′ → α + β (basketweave) transformation takes place, while the globularization of the α-phase occurs from 670 °C. At the same time, the authors showed the presence of β nanoparticles inside the α laths (Figure 35d) that increase with temperature (Figure 35e,f).

**Figure 36:**
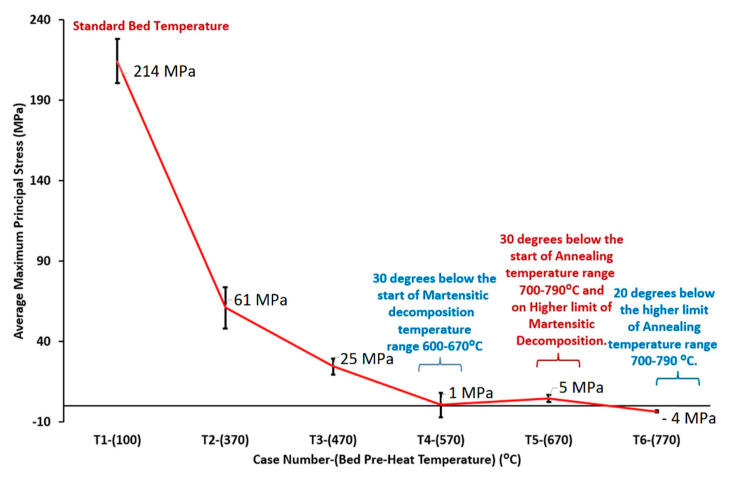
The trend of the residual stress in relation to the BP temperatures (Reprinted from reference [226]).

Simultaneously, the pre-heated BP induces a residual stress reduction, as described by the decreasing trend shown in Figure 36 [226,231]. Malỳ et al. [231] also emphasized a residual stress reduction that increased the *ED* from 65.5 to 83.3 J/mm^3^.

**Figure 37:**
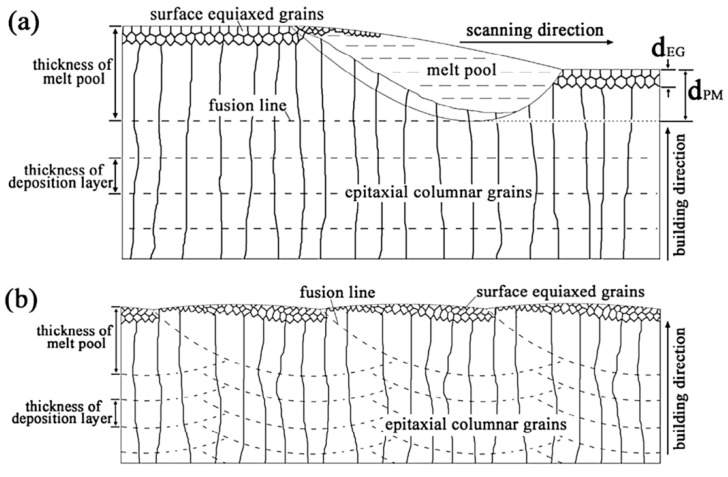
Schematic representation of the columnar β-grains during the AM process in longitudinal (**a**) and transvers (**b**) cross-section (Reprinted from reference [238]).

During the L-PBF process, the β-grains originate from the base and grow up through each deposited layers generating a string texture along the build direction (i.e., <100>, parallel to the heat extraction direction) [32,59,232,233,234,235]. Moreover, in this case, the L-PBF process is characterized by a high cooling rate (up to 10^8^ K/s, [236,237]); focusing on G/R and G × R factors, the L-PBFed Ti6Al4V is composed of columnar or columnar-mixed-equiaxed grains, which are distributed as proposed in Figure 37 by Saboori et al. [238].

De facto, the partial remelting of the previously solidified layer (Figure 4a) and the steeper gradient of temperature make more favorable the formation and growth of the columnar grains [239]. Bontha et al. [240] demonstrated that the process parameters variation induces a morphological grain change, as also discussed in Section 1. The same authors showed that the transformation from columnar grains to mixed equiaxed microstructure is possible by increasing laser power or by decreasing scan speed. The same results are obtained by [214,217]. After the β-grains nucleation and growth, the diffusionless β → α′ transformation occurs due to the concomitance of cooling below the M_s_ (575 ≤ T_Ms_ ≤ 800 °C, [241]) at a rate exceeding 470 K/s [242]. In addition, this transformation is fulfilled according to the following crystallographic dependences [243]:

$$\left[111]\right\|{\left(112\right)}_{\beta }\equiv \left[2\bar{1}\bar{1}3]\right\|{\left(2112\right)}_{\alpha^{\prime }};\;\;\;\;\;\;\;\left[111]\right\|{\left(101\right)}_{\beta }\equiv \left[2\bar{1}\bar{1}3]\right\|{\left(1;001\right)}_{\alpha^{\prime }}$$

**Figure 38:**
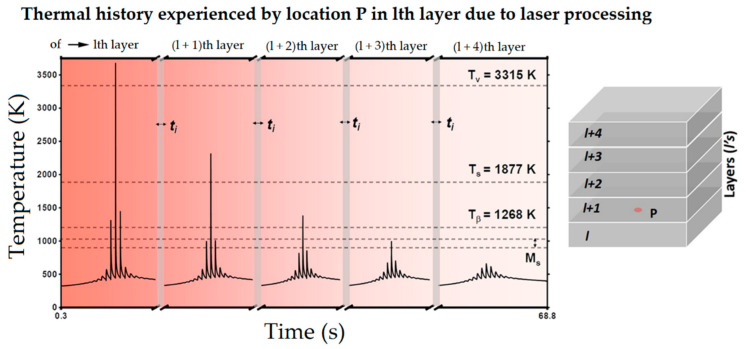
Temperatures reached during the different cycles that characterized the L-PBF process (Reprinted from reference [99]).

Yang et al. [244] reported an interesting point of view about the α′-martensite analyzing their hierarchical structure. As matter of fact, the Ti6Al4V microstructure is formed by columnar β-grains containing primary, secondary, tertiary and quartic α′ due to the thermal cycles generated during the manufacturing process (Figure 38).

**Figure 39:**
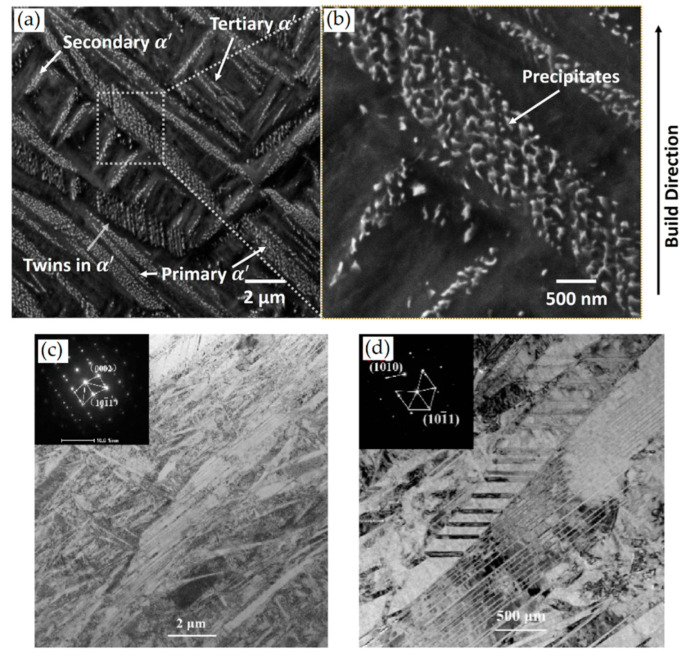
(**a**,**b**) SEM micrographs of as-built Ti6Al4V microstructure showing: (**a**) the primary, secondary, tertiary α′-martensites, (**b**) β precipitates within the α lath; (**c**,**d**) SAED micrographs that highlight the $\left\{10\bar{1}1\right\}$ twinning plane (Adapted from references [99,213]).

Figure 39a–d show the hierarchical structure in addition to the β precipitates and the twin structures. Other authors also highlighted the presence of a high number of dislocations [99,213,244,245,246] that promote the martensite nucleation and the hierarchical structure [244]. Karimi et al. [246] demonstrated that the number of dislocations increases from a single to a triple re-melting during the L-PBF process.

**Figure 40:**
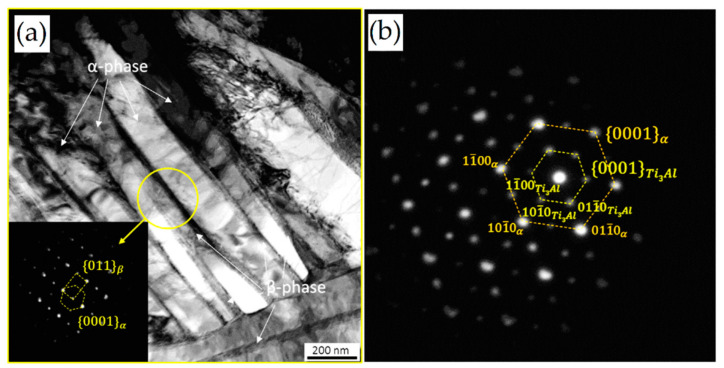
(**a**) BFTEM (Bright-Field Transmission Electron Microscopy) image shows the α + β ultrafine microstructure of the as-built Ti6Al4V sample which highlight the following Burgers orientation relationship ${\left\{011\right\}}_{\beta }∥\;{\left\{0001\right\}}_{\alpha }$, (**b**) SAED patter showing the α_2_-Ti_3_Al precipitate within the α lamella (Reprinted from reference [222]).

Focusing on the cycle related to the (l + 3)th layer, into the same diagram temperature-time shown in Figure 38, the α′-martensite can decompose generating α′ + β + α final microstructure [99,242,244]. In addition, due to the high heat inputs, developed during the L-PBF process, the α_2_-Ti_3_Al can precipitate (Figure 40) due to its precipitation temperature in the range 500 ÷ 650 °C [222,247,248].

This spheroidal α_2_ precipitate formed by 25% of Al, is essential during the AA HT [249,250]. Figure 40a shows the α + β ultrafine microstructure of an as built Ti6Al4V sample containing the α_2_-Ti_3_Al precipitate highlighted by the electron diffraction pattern shown in Figure 40b [222].

Thus, the L-PBF process allows reaching the precipitation temperature range for a sufficient time to form α_2_ precipitates. In fact, some authors showed that α_2_ precipitates in the range of 500–600 °C for several hours [222,241,251]. Dear et al. [251], considering the as-cast Ti-7Al, showed an increase in the intensity of superlattice reflections of α_2_-Ti_3_Al through TEM micrographs after 500 °C × 2 h (AC). The intensity increases significantly after 240 and 2880 h. In this scenario, not only the thermal gradient reached during the L-PBF process but also the Al and O contents promote the α_2_ transformation due to their effects on the α + α_2_ phase-field [252,253].

In relation to the α′-martensite, which is an acicular vanadium supersaturated phase, it crystallizes into hexagonal closed packed (hcp) as well as the equilibrium α-phase (Section 1). Due to the difference in V content, these phases should be characterized by several lattice parameters (a, c) and c/a ratio; however, the values shown in Table 7 do not highlight important differences. De facto, the peaks related to the α′ and α phases are in the same position, and the labels α′, α′/α and α can be achieved after a careful analysis of the presence of β peaks and after scanning and/or transmission microscopy measurements (Figure 34) [221]. The absence of the peaks of the β-phase indicates that its amount is lower than the detection limit, so the microstructure can be considered fully martensitic. On the other hand, the spectra showing the peaks related to the β-phase do not discriminate the presence or absence of the α′-martensite, especially after heat treatments at low temperatures (Section 6.2).

**Figure 41:**
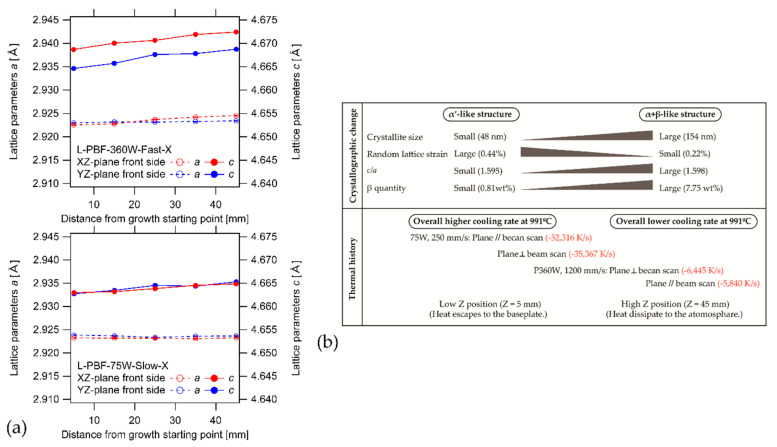
Lattice parameters variation in relation to: (**a**) *z*-axis, (**b**) thermal history (Adapted from reference [257]).

On the other hand, Takase et al. [257] showed a lattice parameters variation in relation to the distance from the pre-heated BP (*z*-axis) at 520 °C (Figure 41a) due to the different cooling rate during the manufacturing process that induces a α′ and α phases transformation. These obtained results are in opposition to what is discussed in Figure 33. The same authors also showed an increase of c/a ratio from 1.595 to 1.598 considering the α′ and α phases, respectively (Figure 41b). All of these reflect on the results obtained by the X-Ray diffraction analysis where the α and α′ phases are considered together due to the peaks overlap [111,227,230,258,259]. In this scenario, Sallica-Leva [229] reported the analysis of the FWHM to discriminate the presence of the α′ and α phases. Focusing on Figure 41a, Takase et al. [257] demonstrated that Ti6Al4V structure varies in relation to the Z position, while the process parameters remain the same due to the different cooling rates obtained. Figure 41b, however, highlighted the influence of the process parameters variation.

The other phase that can be formed during the solidification process is the orthorhombic α″-martensite. Fleißner-Rieger et al. [260] proposed a schematic representation of the β → α″ → α transformation where, due to the heat inputs, the equilibrium structure is not perfectly matched and the intermediate α″-martensite can be nucleated. On the other hand, the β → α′ transformation occurs where the atomic movement is completed, and the hcp structure is formed. The same authors reported the following lattice parameters: a = 2.96 Å, b = 5.05 Å and c = 4.68 Å (c/a = 1.581); according to Brag’s law $\left(2d_{hkl}\sin\theta =n\lambda \right)$, the α″ orthorhombic phase changes the peaks obtained into XRD spectra. Kazanteva et al. [261] and Requena et al. [262] reported the same results. Motyka et al. [243] affirmed that the α″-martensite can be formed with opportune atom volume, electrons’ concentration, and valency (elements with valency > 4 as V).

At the same time, the supersaturated α′ and α″ martensites show a need-like structure. Finally, different studies conducted by [258,259,261,263,264] reported the following crystallographic relationships among all microstructures present in as-built Ti6Al4V samples:

$${\left(011\right)}_{\beta }\|{\left(0001\right)}_{\alpha }$$

$${\left(110\right)}_{\beta }\|{\left(0001\right)}_{\alpha^{\prime }}$$

$${\left\{110\right\}}_{\beta }\|{\left\{001\right\}}_{\alpha^{\prime \prime }}$$

Due to both the previously described Burgers relationship and the self-accommodation of the α′ phase, the martensite phases are inclined by 0, 30, 60 and 90° to each other [59,244,265].

### 6.2. Heat-Treated Microstructure

Table 8 shows the heat treatments, and their nomenclature, analyzed in the present review. Focusing on the Ti6Al4V phase diagram (Figure 1b), these heat treatments must be subdivided in relation to the β-transus, namely, above and below 995 °C. De facto, only the SHT and HIP are subdivided into βSHT, βHIP $\left(T>T_{\beta_{\mathrm{Tr}}}\right)$ and α + βSHT and α + βHIP $\left(T<T_{\beta_{\mathrm{Tr}}}\right)$ in relation to the temperature reached during the heat treatments.

**Figure 42:**
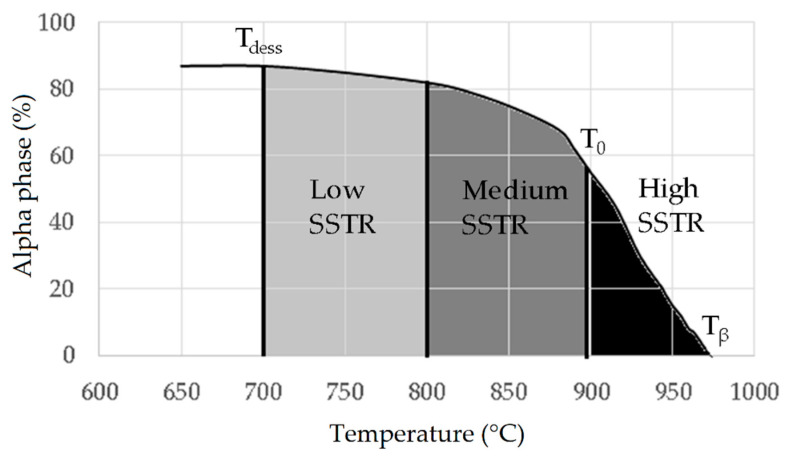
α-phase vol% versus the HT temperatures (Reprinted from reference [245]).

On the other hand, the temperatures reached during the SR, ANN and AA HTs are in the α + β region. The only distinction among these heat treatments is the subdivision proposed by Haar et al. [245], who showed the following categories (Figure 42):

- low-SSTR (Solid Solution Temperature Region): T_dess_ < T ≤ 800 °C
- medium-SSTR: 800 < T ≤ 900 °C (~T_0_)
- high-SSTR: 900 < T ≤ $T_{\beta_{\mathrm{Tr}}}$

T_dess_ is the α-phase dissolution temperature from which the α → β transformation takes place. In fact, the T_dess_ line shown in Figure 42 decreases with increasing temperature up to the β-transus. In addition, T_0_ is defined as a critical temperature that is a temperature range between 872 and 893 °C, from which the α′-martensite can be obtained after a fast cooling (WQ or AC) [245,281,282].

**Figure 43:**
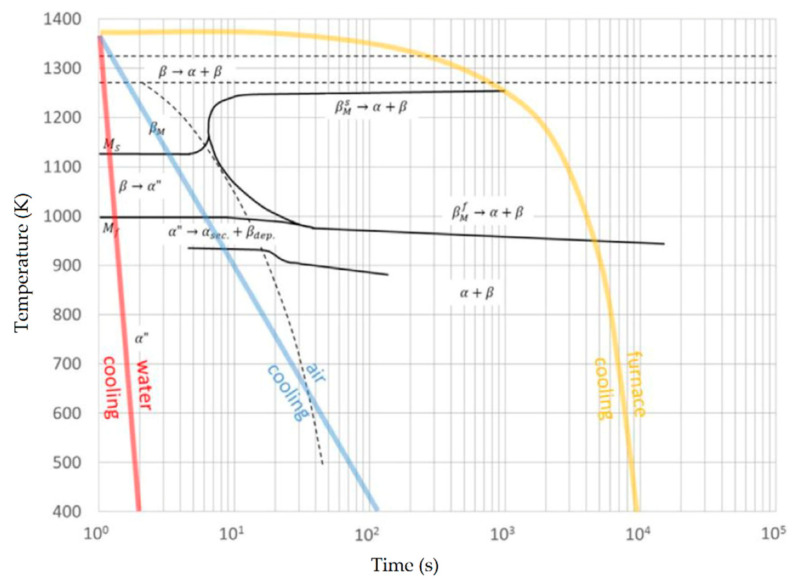
Continuous cooling transformation curve of Ti6Al4V alloy (Reprinted from reference [29]).

In this scenario, not only is the temperature reached during the heat treatment fundamental, but also the following cooling method is observable in the CCT (Continuous Cooling Transformation) curves illustrated in Figure 43.

At the same time, as discussed in Section 6.1, the α′ martensite decomposition already takes place at 400 °C, while the α-phase decomposition occurs around 700–705 °C and continues with an exponential decrease in the β-transus. Table 9 shows the microstructure obtained after different HTs [59,245,269,283].

Starting from the as-built microstructure formed by fully α′ martensite (pre-heated BP at T < 400 °C, [226]), the SR HT does not induce any morphological variation up to 640–650 °C, where Brown et al. [225] showed the presence of β phase through the XRD spectra indicating the α′ → α + β transformation. The same authors reported a complete β transformation at 1008 °C. Lekoadi et al. [212] did not show any observable microstructural changes after 700 °C × 2 h (FC), while Simonelli et al. [59] highlighted the α′ → α + β transformation after 730 °C × 2 h (FC) showing a microstructure formed by α + β structure.

In the same temperature range, Eshawish et al. [268] confirmed the presence of the β phase after the SR at 704 °C × 2 h (FC), while Malinov et al. [284] determined the β phase nucleation at 650 °C confirming the results shown by [225]. Longhitano et al. [271] reported the same results after 650 °C × 3 h (FC). Finally, the morphology of the columnar β-grains is not affected during the SR heat treatments, while the quartic α′ is transformed into β phase and the other (primary, secondary and tertiary) into α phase (Table 9) [59,212,227,233,245,265,268,271,285].

**Figure 44:**
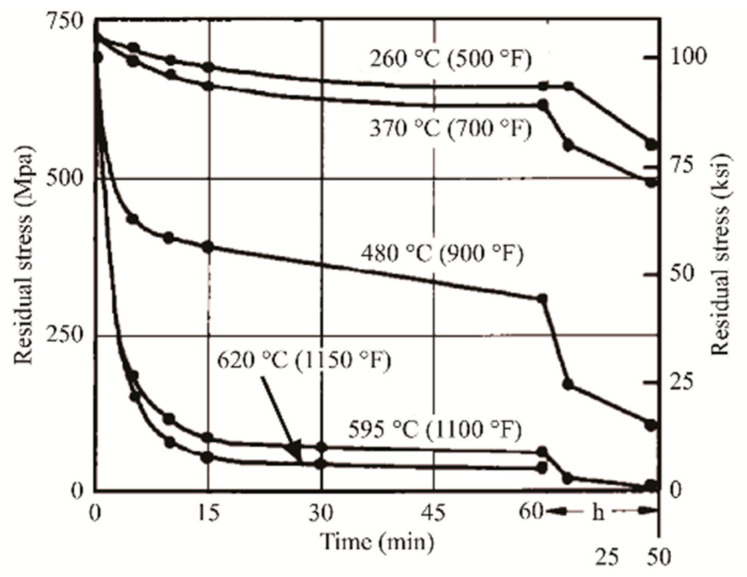
Residual stress trends in relation to SR temperatures and time (Reprinted from reference [289]).

Figure 44 illustrates the focal point of the SR heat treatment, namely, the residual stress reduction in as-built L-PBFed Ti6Al4V samples.

The same graph highlights that the time required to obtain an acceptable stress reduction is strictly related to the SR temperature. In this scenario, an appropriate choice of this temperature should be necessary due to the following reasons: the microstructural variations as previously discussed, and the precipitation phenomena of β nano-particles, α, β phases and of the α_2_-Ti_3_Al [230,247,268,271,285,290]. As concerns the β nano-particles, Etesami et al. [285] showed the presence already in as-built Ti6Al4V samples manufactured on cold BP. The same results were obtained by Ali et al. [226]. The presence of the α_2_-Ti_3_Al particles is caused by its precipitation temperature range and for the fact that the as-built samples are SSS (see Section 6.1). De facto, some studies reported a solute distribution and diffusion during the HTs, confirming the obtained microstructural variations and precipitation phenomena [225,230,265]. Thus, the SR heat treatment parameters must be selected to obtain the excellent reduction in the residual stress and not to incur precipitation phenomena.

The α′-martensite decomposition and the α, β grains growth increase with the temperature increasing, i.e., considering the ANN heat treatments. De facto, the range temperature between 750 ÷ 900 °C fully decomposes the α′-martensite into α platelets and induces the β-phase formation due to the V diffusion [225,230,265]. Clear differentiation between the ANN and SR heat treatments are not reported through the literature; however, Table 8 illustrates this in the scopes section. In terms of microstructure, Huang et al. [265] reported α + β structure with a regular arrangement caused by the self-accommodation of α′ martensite. In addition, they affirmed that the presence of defects induced by the L-PBF process impedes the grain boundary movements restricting the grain growth. On the other hand, already at ANN temperatures, the holding time and the cooling method become significantly more important to the microstructure morphology (Table 9). Haar et al. [245] obtained a greater grain growth (1.5–3.5 μm) of the α grain width in sample heat-treated at 870 °C × 2 h (FC) than in the sample (1.5–2.5 μm) heat-treated at 870 °C × 4 h (AC). Etesami et al. [285] showed an increase in size and volume of α and β with time, in addition to the increase in secondary α phase and the formation of rod-shaped β between the laths of α phases.

The same authors reported the β → α′ martensite transformation after 930 °C × 2 h (WQ), confirming the T_0_ critical temperature, while secondary α phase into primary α phase was obtained after 930 °C × 2 h (AC). The FC cooling, however, induces a complete microstructural change into α + β, where α globularized and β is distributed into α laths.

**Figure 45:**
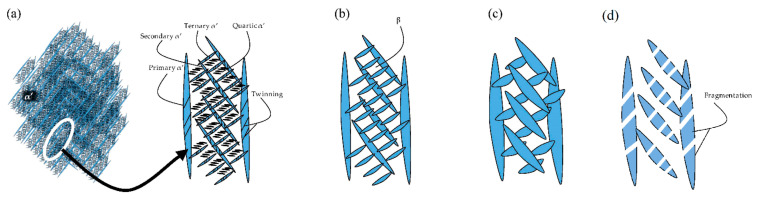
Hierarchical structure of α′-martensite in as-built condition (**a**), and of the α-phase after the ANN heat treatments in low-SSTR (**b**), medium-SSTR (**c**) and high-SSTR (**d**) (Adapted from reference [245]).

A schematic representation of the microstructural variation (Figure 45) is reported by Haar et al. [245], who analyzed different HTs in the low-, medium- and high-SSTR. During the ANN below the T_0_ (T < 900 °C), α′ → β begins from the smallest quartic α′, while the other phases coarsen. Increasing the holding time at high temperatures, the α′ → β transformation also occurs from the larger grains (first the tertiary and then the secondary martensites) (Figure 45a–c). At the same time, the β phase nucleates and grows at the grain boundaries and the complete β-phase transformation from the primary α′-martensite occurs at T > $T_{\beta_{\mathrm{transus}}}$. As also shown in Table 9, the ANN microstructures obtained at high temperatures reach a primary α′ → α transformation. On the other hand, increasing the temperature above the T_0_, the fragmentation of primary and secondary α grains (Figure 45d) takes place due to the β-phase formation into twinning sites. In addition, the grain growth increases after the fragmentation process due to the increase in the surface energy minimization. 

After a high cooling rate, the α′-martensite becomes a matrix where the fragmented primary and secondary α phases are. Thus, a subsequent ANN HT in low-SSTR can be considered to obtain the α′ → α transformation.

The AA HT, which can be directly performed on as-built Ti6Al4V samples, can be used to reduce the residual stress without excessive microstructural changes (Table 9 and Table 10). On the other hand, its focal points are the balance of the tensile strengths and ductility with a bi-modal microstructure (as will be discussed successively in Section 6) and the precipitation of α, β and α_2_-Ti_3_Al [179,224,271,276,285,291,292].

For these reasons, and for the fact that the solvus temperature of the α_2_-Ti_3_Al is around 500 °C, G. Lütjering [292] affirmed that the heat treatments at 600 °C or above will only change the microstructure and reduce the residual stress. Gehlin et al. [249] reported that the crystallographic ordering of Al can occur in the range between 500 and 700 °C, which leads to the precipitation of the α_2_-Ti_3_Al. De facto, long-term aging performed at 500–600 °C produces precipitates between 5 and 10 nm. In addition, the α_2_-Ti_3_Al formation is influenced by the alloying elements as oxygen and the other β-stabilizers [293,294].

**Figure 46:**
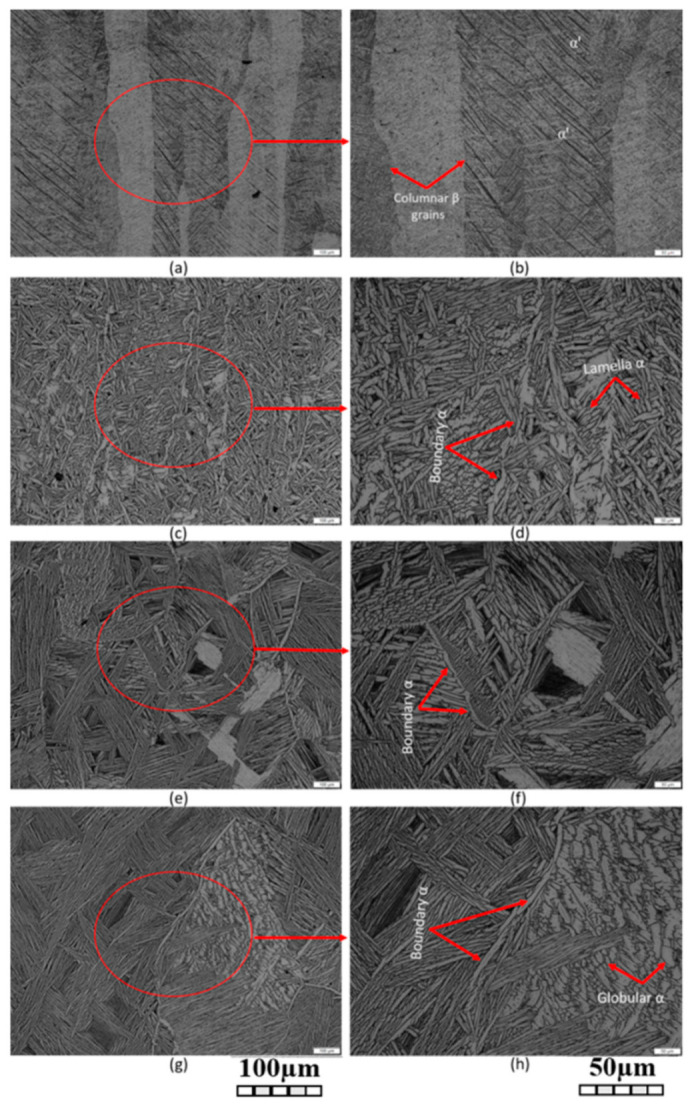
OM micrographs of Ti6Al4V microstructures in as-built condition (**a**,**b**) and after the HTs at the following temperatures: (**c**,**d**) 700 °C, (**e**,**f**) 950 °C and (**g**,**h**) 1000 °C (Reprinted from reference [212]).

From a mechanical point of view, the microstructure obtained after the AA heat treatments is more important than the precipitation phenomena due to the strict relationship between the microstructure and the fracture mechanisms under different load conditions [59,295,296,297,298]. De facto, the temperature reached during the βSHT $\left(T_{\mathrm{SHT}}>T_{\beta_{\mathrm{transus}}}\right)$ or the α + βSHT $\left(T_{\mathrm{SHT}}<T_{\beta_{\mathrm{transus}}}\right)$ and the subsequent cooling method induce different microstructures, such as α′-martensite, α-colonies, plate-like α, acicular α, grain boundary α, basketweave or Widmanstätten structure into equiaxed or columnar β-grains (Table 9) [59,227,230,233,265,271,276,285]. As regards the holding or residence time at high temperatures, the main effect is the grain growth, which is more effective at near β-transus temperature. In fact, considering the heat treatments into low-SSTR, the α and β grains tend to grow, but will hinder each other [32]. Into high-SSTR or, even above the β-transus, the holding time has a greater influence on the microstructural morphology and size. Eshawish et al. [268] also reported the presence on the columnar β-grains after 1015 °C × 15 min (WQ), unlike Wu et al. [233], who instead showed the recrystallization process (columnar → equiaxed β grains) after 1000 °C × 40 min (WQ). In this context, Huang et al. [265] reported the same transformation after 1050 °C × 1 h (AC), suggesting that the equiaxed grains form from the split of the columnar grains. In fact, the same authors showed that the diameter of the equiaxed grains is the same as the columnar width. Lekoadi et al. [212] reported an incomplete β-grains transformation after 1000 °C × 2 h, a fully α′ martensite decomposition into α + β lamellar with α-colonies after 4 h, and a globularization after 8 h (Figure 46).

As previously reported, the other focal point is the cooling method; however, in this case, it also depends on the temperature from which the cooling pathway starts. The effects induced by the cooling rate are moderate if the samples are heat-treated in low-SSTRs, but they become very important when the samples are at temperature > T_0_, as previously discussed [32,212,265,271,276,285]. Focusing on this last temperature range, Etesami et al. [285] reported a microstructure formed by primary α-phase containing β-particles and needle-like α′-martensite after 930 °C × 2 h (WQ). The same authors showed a mixture of primary α and secondary α + β-phase structure after 930 °C × 2 h (AC), which change in total α + β lamellar after the same heat treatment followed by furnace cooling. Lekoadi et al. [212], who analyzed the same cooling methods and holding time at 1000 °C, showed a fully α′ martensite structure contained into columnar β-grains after both the WQ and AC. As reported by Huang et al. [265], it is possible to underline that this α′-martensite is different to that formed during the L-PBF process due to the different responses to the heat treatment. The former decomposes more easily than the latter after 800 °C × 1 h (AC). Finally, only the FC promoted the basketweave structure formed by lamellar α + β. In relation to the cooling pathway, Muhammad et al. [276] demonstrated that the 950 °C × 1 h (FC) rather than the 950 °C × 1 h (AC) allows one to dissolve a large part of the nanoparticles present within the α-phase laths.

The α′-martensite decomposition and the α → α + β transformation are also obtained after the HIP HT due to the temperature to which the treatment was carried out. Moreover, in this case, it can be subdivided into β-HIP $\left(T>T_{\beta_{\mathrm{Tr}}}\right)$ and α + β-HIP $\left(T<T_{\beta_{\mathrm{Tr}}}\right)$ and the following obtained microstructure depends on both the holding time (generally 2 h) and cooling pathway (Table 9). Benzing et al. [299], who analyzed the additive manufactured Ti6Al4V samples, showed the columnar → equiaxed β-grains transformation after (1050 °C × 2 h (WQ) + 100 MPa) + (800 °C × 2 h + 30 MPa) and a greater microstructural homogeneity than after HIP at 920 °C × 2 h + 100 MPa. At the same time, a significant increase in the grain size from the as-built (α + β) to the HIP samples is not highlighted. On the other hand, the greatest coarsening of the α laths starting from α′-martensite was obtained after 1050 °C × 2 h + 100 MPa rather than after 920 °C × 2 h + 100 MPa, as shown in the study of Leuders et al. [110]. The vast majority of the HIP heat treatments reported on different research were performed in the temperature and pressure ranges of 900–950 °C and 100–150 MPa, respectively; the α + β lamellar structure is the microstructure obtained [110,111,223,268,288,300]. In this scenario, not only the $T>T_{\beta_{\mathrm{Tr}}}$ influence the α laths coarsening but also the $T<T_{\beta_{\mathrm{Tr}}}$, as reported by Wycisk et al. [301], who showed a α laths coarsening from ~1 μm to 4 μm. The same results are shown by Mahmud et al. [111], who reported a linear increase with time. To avoid an excessive grain coarsening, Herzog et al. [302] proposed an increase in pressure to 200 MPa and a consequent decrease in temperature up to 820 °C. Other studies, however, proposed different cooling pathways to control the α lamellar dimensions after the HIP heat treatments (from 500 nm of the α′ to 3 ÷ 60 μm of the α phase) [223,287,301,303].

Apart from the microstructural effects, the HIP heat treatment allows for obtaining the sample densification through the reduction in LOF, keyhole and gas pores, cracks, etc., (see Section 2) present into as-built samples [111,223,268,300,302]. Herzog et al. [302] demonstrated that a fully dense (δ > 99.9%) sample can be obtained if its density in as-built conditions is higher than 95%. In fact, they reported an increase from 93.4 to 98.7% and from 97.7 to 99.9%, respectively, after the HIP at 820 °C × 2 h + 200 MPa. The densification effects are also supported in the study conducted by Eshawish et al. [268], where the volume fraction of the spherical pores decreases from 0.31 ± 0.2% to 0.20 ± 0.09% after 900 °C × 2 h + 100 MPa. The same HIP heat treatment induced the decrease in the maximum pore’s diameter from 140 μm to 15–21 μm. Therefore, the HIP heat treatment induces sample densification, allowing an expansion of the process window illustrated in Figure 8 [302]; however, if the as-built samples is manufactured with optimized process parameters, their microstructure can be optimized through other heat treatments.

**Figure 47:**
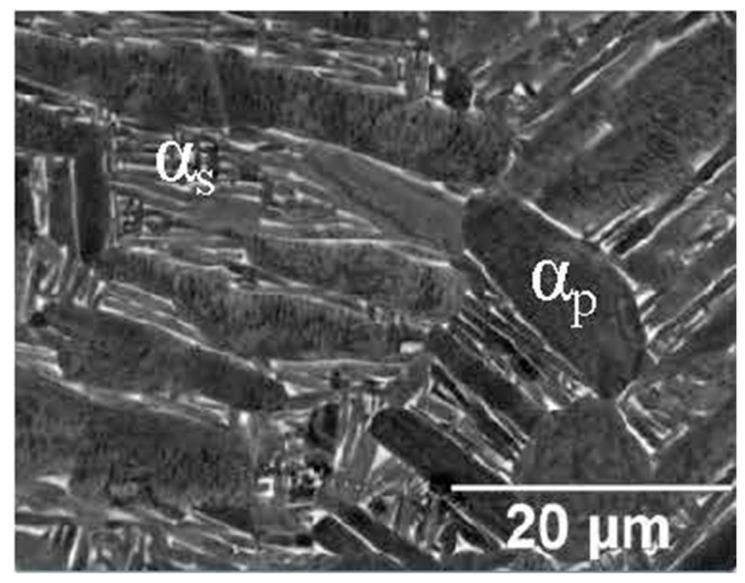
Bi-modal microstructure after [910 °C × 8 h (WQ)] + [750 °C × 4 h (FC)] where α_p_ and α_s_ indicate primary and secondary α-phase, respectively (Reprinted from reference [245]).

From a last microstructural point of view, L-PBFed Ti6Al4V samples can present a bimodal microstructure after heat treatment to obtain a good balance between strength and ductility. Chong et al. [304] reported that the so-called bimodal microstructure is a “dual-phase” composed of the primary α_p_ phase and secondary α_s_ phase transformed by β-phase, as reported in Figure 47.

Due to the first annealing performed in high-SSTR, the primary α_p_ phase tends to assume a globular morphology, while other parts of the microstructure transform into β phase due to the diffusion of the alloying elements (see T_dess_ in Figure 42). Sabban et al. [305] confirmed the diffusion of the alloying element during the first heat treatment through the EMPA element maps. The microstructural zone is characterized by a higher amount of V involved in β → α_s_ transformation (Widmanstätten structure) due to the subsequent heat treatments [245,304,305]. The cooling method, and the related cooling rate, will affect the secondary lamellar α_s_ size. Zhao et al. [306] affirmed that the first globularization process initiates due to the α′-martensite splitting during the first heat treatment.

In addition, the applied HT induces a texture variation highlighting an increase in intensity along with the directions parallel to the build orientation, as studied by Sabban et al. [305].

In this scenario, Haar et al. [245] showed a bimodal microstructure after the duplex annealing HTs (910 °C × 8 h (WQ) + 750 °C × 4 h (FC)).

Bai et al. [224] and Sabban et al. [305] reported, however, a bimodal structure after the same cycling annealing between 975 and 875 °C. The first study shows a sequence of nine heating and cooling steps in 24 h characterized by rates of 2.5 °C/min and 1 °C/min, respectively, while the second illustrates a cycling annealing formed by five steps of heating (3.33 °C/min) and cooling (1.67 °C/min) with a holding time of 30 min at 975 °C for each step. De facto, the obtained microstructure shows a globular α_p_ and a lamellar α + β Widmanstätten structure representing the α_s_ phase.

#### Heat Treatment Effects on α′, α and β Phases

**Figure 48:**
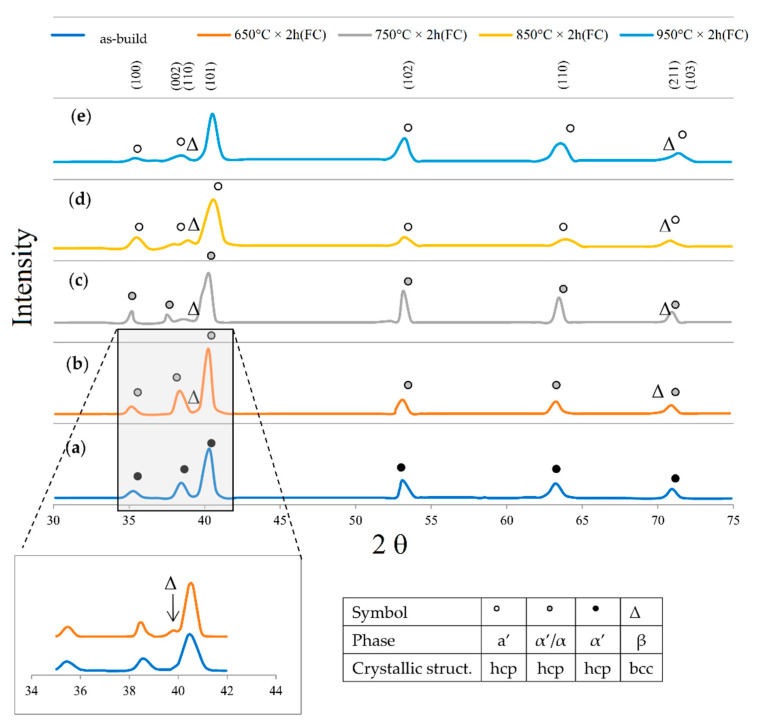
XRD spectra related to the as-built (**a**) and heat-treated (**b**–**e**) Ti6Al4V samples at: (**b**) 650 °C × 2 h (FC), (**c**) 750 °C × 2 h (FC), (**d**) 850 °C × 2 h (FC), (**e**) 950°C × 2 h (FC) (Reprinted from reference [307]).

During the heating of the previously analyzed heat treatments, the coarsening of α′-martensite, its decomposition and transformation into α + β, α coarsening, α globularization and β → α transformation can occur with the temperature increasing. During the subsequent cooling pathway, the microstructure can change in relation to the cooling rate. Starting from a fully martensitic microstructure of a L-PBFed as-built sample, its XRD spectra do not reveal any β-peaks correlated, as discussed in Section 6.1 and shown in Figure 34 and Figure 48 [227,230].

**Figure 49:**
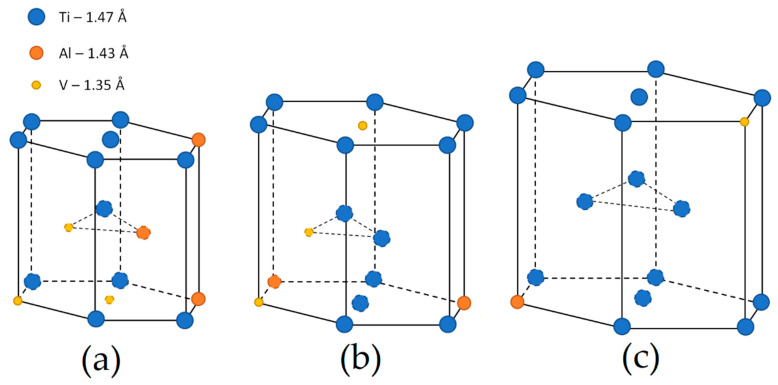
(**a**–**c**) Lattice structure transformation from α′-martensite to equilibrium α-phase at: (**a**) 25 °C, (**b**) 550 °C and (**c**) 700 °C.

Only increasing the temperature to 550 °C, Kaschel et al. [227] revealed a broad shoulder peak between the base of the ${\left(10\bar{1}1\right)}_{\alpha /\alpha^{\prime }}$ and the ${\left(110\right)}_{\beta }$ directions, indicating the α′ → α + β decomposition, in addition to a new ${\left(0002\right)}_{\alpha }$ between 37 and 38.6°. On the other hand, Eshawish et al. [268] did not show any peak related to the β-phase even after 704 °C × 2 h, nor do Cao et al. [230], who illustrated the β {100} peaks only after 800 °C × 2 h. On the other hand, Mahmud et al. [111] showed the same peaks after 670 °C × 5 h and Mierzejewska [307] after 650°C × 2 h (FC) as shown in Figure 48. The development of the XRD spectra is, therefore, caused by the alloying element diffusion, which induces not only the phases transformation but also the stress relaxation. All of this is strictly related to the crystal lattice evolution shown in Figure 49 where the α′-martensite evolves into equilibrium α phase at 700 °C. The first considerable variation of the lattice parameters takes place at 400 °C due to the internal diffusion and self-accommodation of the Al and V alloying elements. As matter of fact, the α′-martensite decomposition begins at 400 °C (see Section 6.2); other changes at temperatures lower than 400 °C can be induced by a thermal expansion of the lattice structure [227,308]. A higher increase in lattice parameters is shown at 550 °C, where the V atoms diffuse out of the lattice structure and the Ti atoms replace them. De facto, the increase in lattice parameters is caused by the Ti radius (1.47 Å), which is higher than the V radius (1.43 Å) [309]. Finally, the α′ martensite is fully decomposed in the equilibrium α phase at 700 °C (Figure 49c).

At the same time, Kaschel et al. [227] showed the c/a ratio variation with temperature, and that the ratio increased significantly only after 500–550 °C. The same authors suggested that the α → β transformation induces a decrease in the c/a ratio after 995 °C. In addition, Tsai et al. [310] showed the variation of the β-phase lattice parameters from 3.188 to 3.244 Å into a temperature range of 550–800 °C.

**Figure 50:**
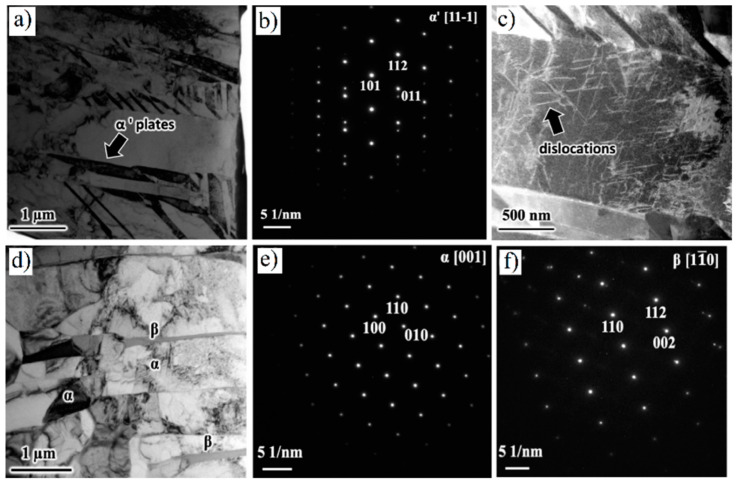
BFTEM images (**a**,**d**), SAED patterns (**b**,**e**,**f**) and HAADF STEM (**c**) images of SR (**a**–**c**) and ANN (**d**–**f**) Ti6Al4V samples (Adapted from reference [111]).

During the same heat treatments, in particular, during SR and ANN, the twin structures (Figure 39) and the number of dislocations present into α′-martensite laths are reduced. In detail, the twin structures are fully dissolved only after 800 °C × 6 h [230]. The same results were obtained by Li et al. [287] and Tsai et al. [310]. On the other hand, the number of dislocations was deeply reduced after the HIP at 920 °C × 12 h with the formation of α + β lamellar microstructure rather than after the SR at 670 °C × 5 h as illustrated in Figure 50 [111].

**Figure 51:**
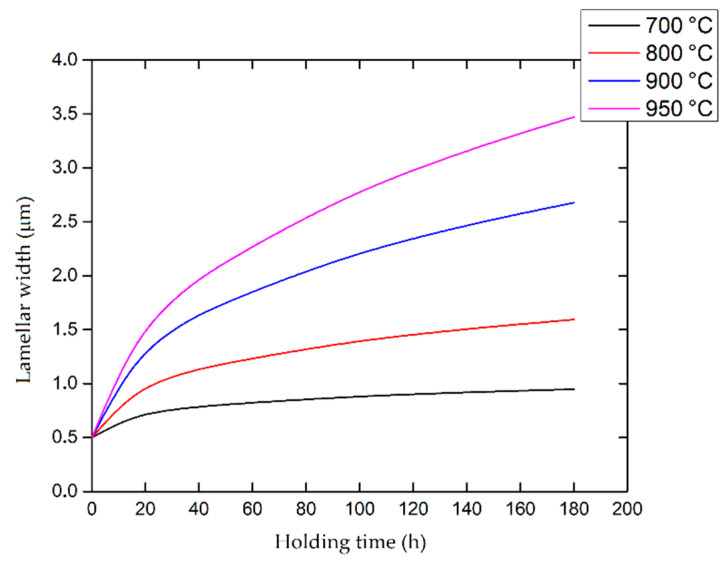
Coarsening effects of lamellar α width in relation to the heat treatment temperatures (700, 800, 900 and 950 °C) and time (0 ÷ 180 h) using Equation (16).

Together with the decrease of the dislocations, twin structures, and α′ → α + β transformation, the already formed α platelets tend to coarse if the temperature of the heat treatment exceeds the low-SSTR (i.e., the temperature of α′ → α + β decomposition). In fact, Figure 51 shows a slight coarsening effect at 700 °C, which increases as fast as the increase in temperature. This trend is described by the following relationship:

(16) $$\delta_{\alpha_{\left(t=t^{*}\right)}}=\delta_{\alpha_{\left(t=0\right)}}t^{\left(\frac{T-850}{1000}\right)}\;$$

where the $\delta_{\alpha_{\left(t=0\right)}}$ is the average lamellar width (μm) of the as-built Ti6Al4V sample, namely with *t* = 0 of HT, *t* and *T* are the time (h) and temperature (K) of the HT [311].

Considering the tangents of all curves shown in this graph and their slope, the coarsening rate increases with temperature but decreases with time.

**Figure 52:**
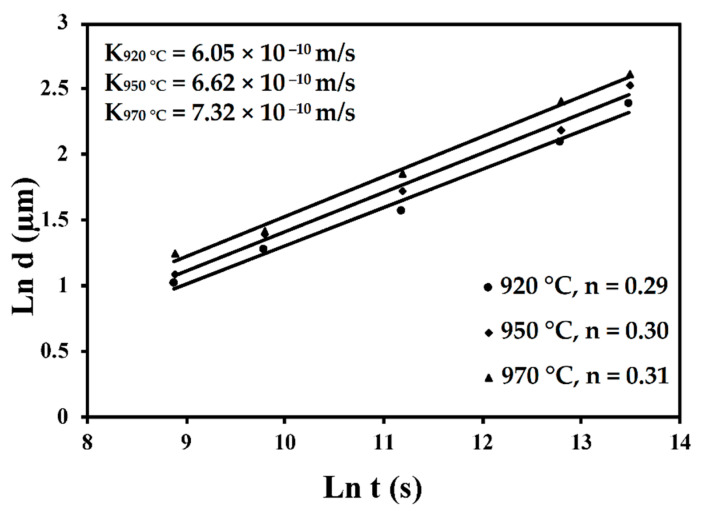
Plots of Equation (18) into ln-ln diagram considering the coarsening effects induced by the heat treatment temperature at 920, 950 and 970 °C (Reprinted from reference [111]).

In this scenario, the lamellar coarsening of the α-phase width follows the coarsening theory (LSW) developed by Lifshitz, Slyozov and Wagner [312,313]:

(17) $$d=\xi t_{HT}^{n}$$

where the *d* is the average lamellar width (μm), ξ is a constant of proportionality, *n* is the coarsening coefficient and *t* is the heat treatment time [s]. This equation can be rewritten into logarithmic form, as follows:

(18) ln(d)=ln(ξ)+nln(t)

assuming a linear trend if it is plotted into an ln-ln diagram (Figure 52, [111]). As is also shown in the same figure, Mahmud et al. [111] reported that n is equal to 0.29, 0.30 and 0.31 for 920, 950 and 970 °C, respectively, while ξ is equal to 6.03∙10^−10^, 6.62∙10^−10^ and 7.32∙10^−10^ m/s, respectively. Moreover, 0.33, 0.33 ÷ 0.40, 0.40 ÷ 0.50 were obtained at 700 ÷ 800 °C, 900 and 950 °C by Cao et al. [314]. The same results were obtained by Liu et al. [311] who confirmed that the coarsening kinetics model of the α-phase follows the LSW theory (Equations (17) and (18)).

**Figure 53:**
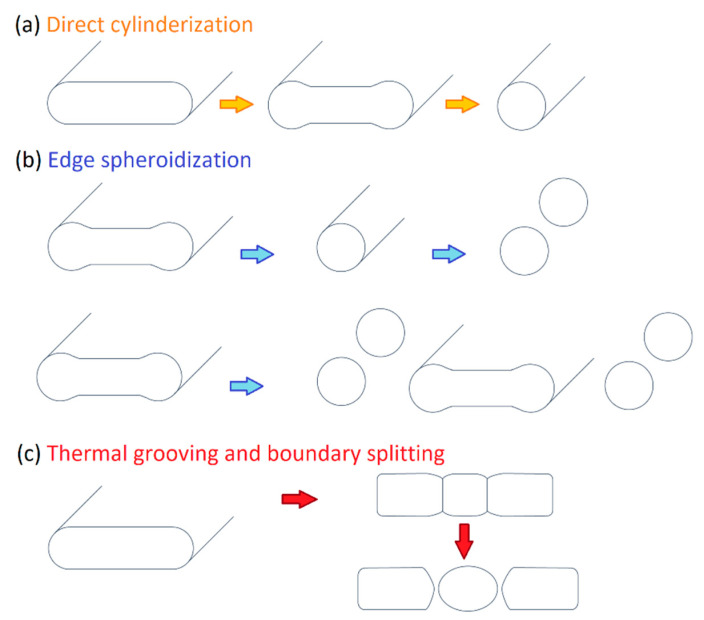
Globularization mechanisms of the α-phase: (**a**) direct cylinderization, (**b**) edge spheroidization, (**c**) thermal grooving and boundary splitting.

Focusing on the HT temperature and on the high holding time, the α-phase also tend to globularize following one of these mechanisms [288,305,315,316,317]:

- direct cylinderization (Figure 53a): initiates at the edge of the α platelet where the different curvature allows for the mass transfer to the flat part of the same platelet. The subsequent formation of these ridges can induce a cylindrical morphology if the ridges join together.
- edge spheroidization (Figure 53b), which differs from the direct cyliderization (Figure 53b) due to eventual perturbations developing along the ridges which separate the lamella into spheroids.
- thermal grooving and boundary splitting (Figure 53c): induce α globularization due to the initial groove’s formation into triple junction generated by sub-grain boundaries into α lamellae with α/β interface, and due to the sequent Al and V diffusion that break the lamella with the β-phase formation.
- termination migration consists of the mass transfer from the curved surface of the lamella to the flat lamella.

## 7. L-PBFed Ti6Al4V: Mechanical Properties

The mechanical properties of the as-built Ti6Al4V samples (Table 10) show high values in terms of ultimate tensile strength and yield strength due to their fully α′ martensitic microstructure. Obviously, high strengths correspond to low ductility (<10%) values that do not satisfy the ASTM F2924-12 standard specification [318]. In this scenario, appropriate heat treatments provide an opportunity to balance the tensile strength and the ductility values (necessarily > 10%). On the other hand, some authors reported good elongation values already after the L-PBF process. Firstly, Xu et al. [221] reported an increase in ductility up to 11.4 ± 0.4%, varying the ED values to obtain an ultrafine α + β microstructure. Secondly, Ali et al. [226] showed 10% of elongation considering the samples manufactured with the pre-heated BP at 570 °C (Figure 35). The same authors have declared that the samples manufactured on pre-heated BP at 670 and 770 °C reached a premature failure due to the different cooling rates observed during the printing process. In this scenario, as well as in the following process related to the heat-treated samples, it is possible to show the influence of the cooling rate on the yield strength and ductility, as proposed by Lütjering [292].

**Table 10:** Mechanical properties of L-PBFed Ti6Al4V samples in as-built conditions.

Process Parameters					*ED* (J/mm^3^)	Directions	Microstructure	E (GPa)	UTS (MPa)	YS (MPa)	A (%)	Ref.
*P* (W)	*v* (mm/s)	*h* (μm)	*t* (μm)	BP (°C)								
--	--	--	--	--	--	--	--	--	895	825	10	[318]
--	--	--	--	--	--	H(xy)	Fully α′ martensite	--	1274 ± 26	1047 ± 23	10 ± 1	[305]
						V		--	1219 ± 32	1043 ± 18	12 ± 1	
200	--	150	150	--	--	--	Fully α′ martensite	--	1191 ± 6	970 ± 6	5.4 ± 1.4	[265]
200	--	80	50	100	--	--	Fully α′ martensite	114 ± 5	1123	1139	6.0	[226]
				370		--	Fully α′ martensite with increased laths ^1^		1234	1159	9.5	
				470		--	α′ → α decomposition		1232	1173	9.7	
				570		--	Colonies α + β		1233	1176	10.0	
200	--	80	50	670		--	Colonies α + β + β nano-particles + α globularization		1201	1174	2.58	[226]
				770		--	Increase the effects obtained at 670 °C	207 ^2^	748 ^2^	--	--	
--	--	--	--	37	--	--	Fully α′ martensite	--	1236 ± 27	1181 ± 34	8.7 ± 0.5	[285]
1000	1400	230	100	70	38.3	H	Fully α′ martensite (< 1μm)	--	1204 ± 27	1052 ± 16	2.1 ± 0.3	[288]
						V		--	1075 ± 80	958 ± 73	1.8 ± 0.2	
800	1925	190	50		43.7	H	Fully α′ martensite (1–2 μm)	--	1176 ± 8	1024 ± 6	2.5 ± 0.4	
						V		--	1086 ± 6	933 ± 7	2.5 ± 0.3	
350	770	180	50		50.5	H		--	1251 ± 114	1067 ± 27	3.4 ± 1.3	
						V		--	1191 ± 19	1065 ± 27	2.4 ± 1.2	
170	1250	100	30	--	45.33	--	Fully α′ martensite	109 ± 4	1218 ± 2	1015 ± 10	5.9 ± 1.0	[271]
375	1029	120	60	--	50.62	V	Ultrafine α + β	--	~1240	1106 ± 6	11.4 ± 0.4	[221] ^3^
280	1200	140	30	100	55.56	--	Fully α′ martensite		1206 ± 23	1041 ± 23	9.6 ± 0.4	[288]
175	710	120	30	--	68.47	V	Fully α′ martensite	--	~1160	--	~9	[221] ^3^
175	710	120	30	--	68.47	H	Fully α′ martensite	--	1321 ± 6	1166 ± 6	2.0 ± 0.7	[319]
250	1600	60	30	--	86.81	--	Fully acicular α′ martensite	--	1267 ± 5	1110 ± 9	7.3 ± 1.1	[32]
157	225	100	50	--	139.56	H(xz)	Fully α′ martensite	115 ± 6	1143 ± 6	978 ± 5	11.8 ± 0.5	[59]
						H(xy)		113 ± 5	1199 ± 49	1075 ± 25	7.6 ± 0.5	
						V		119 ± 7	1117 ± 3	967 ± 10	8.9 ± 0.4	

^1^ possible decomposition into α equilibrium phase. ^2^ Sample reaches the premature failure [226]. ^3^ Samples manufactured with a Focal Offset Distance of 2 mm.

Lütjering [292] showed an increase in yield strength and with the increase of the cooling rate due to the consequent variation in terms of colonies size. The final exponential increment is conferred by the α′-martensite. At the same time, the ductility increases up to a maximum, after which it declines drastically. The author affirmed that, at the maximum point, the fracture mechanism passes from ductile transcrystalline dimple type to intercrystalline dimple type along with the continuous layers of α phase.

**Figure 54:**
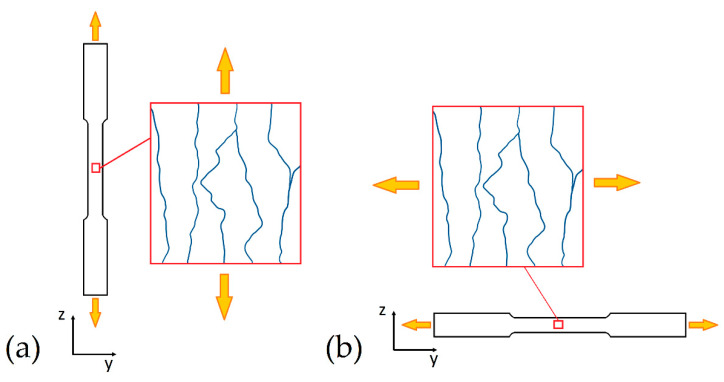
Schematic representation of the load conditions considering: (**a**) V-sample, (**b**) H-sample.

If the *ED* values and pre-heated BP can influence the as-built mechanical properties, another important factor is the build direction of the sample within the build chamber. In this context, it is useful to remember that the columnar β-grains growth occurs through the layers and, therefore, in the perpendicular direction to the BP, regardless of the build orientation (Section 6.1). As reported in Table 10, the samples manufactured vertically for the xy plane show generally lower tensile strengths and ductility than the H-samples due to the relationship between the load direction and the columnar β-grains distribution [59,230,320,321]. Figure 54 illustrates a schematic representation where, if the load is applied along with the major axis of the columnar β-grains (Figure 54a), the β-phase grain boundaries are subjected to Mode I opening tension, while the α-phase grain boundaries are subjected to Mode I opening tension when the load is applied along the short axes of the columnar β-grains (Figure 54b) [320].

**Figure 55:**
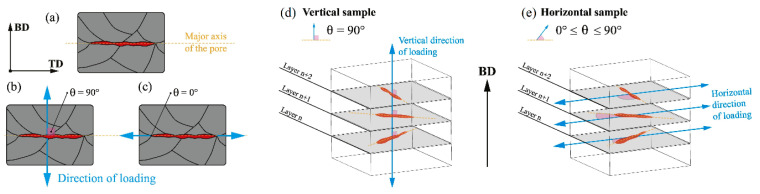
Schematic representation of the load direction and the LOF major axis (**a**) during a tensile test in V-samples (**b**,**d**) and H-sample (**c**,**e**); (**d**,**e**) Schematic representation of the load direction and the different orientation of LOF major axis within the same sample and correlated to the different layers (Adapted from reference [322]).

In the same context, Willson-Heid et al. [321] analyzed and demonstrated that the anisotropic elongation and the β grain aspect ratio (*x*) can be correlated as follows:

(19) $$y=0.00125e^{0.91x}+0.98$$

where *y* is related to elongation. Its anisotropy becomes significant when *x* > 6. Another important factor influencing the anisotropy of the mechanical properties is the presence of the LOF (Figure 55a–c).

If the load direction and the major axis of the LOF are from an angle of 90°, the pores tend to open, inducing a stress concentration on their apexes and possible consequent crack initiation. This configuration can induce a premature failure into as-built sample rather than that shown in Figure 55c, where the angle θ is 0°. In this situation, the LOF pore will be closed; in fact, the void growth and the crack initiation need a greater tensile load [322]. As discussed in Section 2, the presence of LOF pores along the xy plane depend on the process parameters and on the scanning strategy. Thus, the V-samples are more affected by LOF opening during the tensile test than the H-samples (Figure 55d,e), where the angle between load direction and the LOF major axis can vary between 0 and 90° [322].

**Figure 56:**
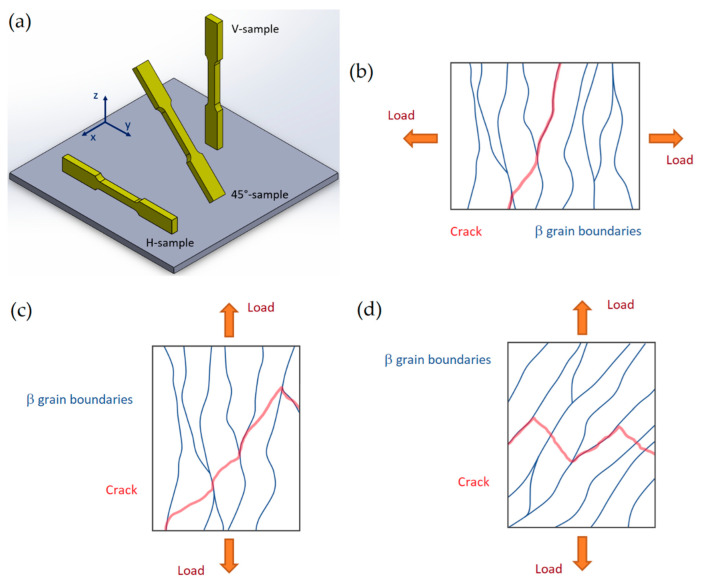
Schematic representation of different Ti6Al4V samples: (**a**) H-, 45°- and V-samples, (**b**–**d**) under the same load conditions but with different orientation between the load axis and the columnar β-grains (blue lines represent the β-grains boundaries).

**Figure 57:**
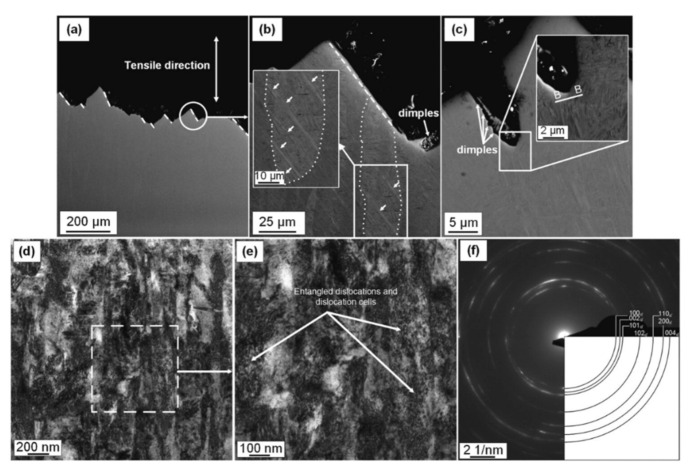
SEM micrographs of as-built Ti6Al4V V-sample after tensile testing (**a**–**c**); (**d**) TEM of the BB section shown in panel (**c**), where the square area is shown in panel (**e**); (**f**) SAEDP image showing the rings related to the α′ martensite (Reprinted from reference [324]).

The different sample’s orientation also affects the fracture mechanisms, as shown in Figure 56, where the grey ellipses represent the columnar β-grains [296]. When the H-sample (Figure 56b) is subjected to a tensile load, the main failure is Mode I with low ductility values obtained. On the other hand, the V-sample (Figure 56c) is characterized by an intergranular fracture much more tortuous than in the H-sample (Figure 56b) [59,295]. Focusing on 45° samples (Figure 56d), the effects of the tensile load must be subdivided into normal and shear stresses and the crack propagation is transgranular [296]. In this scenario, the best mechanical properties depend on a good combination between the build orientation and the presence of defects, which also reduce the layer interconnection and vary with the process parameters, in addition to the dimensions and relative aspect ratio of the β-grains [59,294,321,322]. Simonelli et al. [59] show the typical surface fracture of the as-built Ti6Al4V samples characterized by an almost flat central region and an external portion high inclined at around 45°. Additionally, the same profiles show an intergranular fracture, where crack propagation is strictly related to the crystallographic orientation of the α′ and α phases (see Section 6.1 and Section 6.2) that may arise as single α-phase or as α-colonies. This situation can characterize both the as-built and the SR heat-treated samples where the crack can be deflected due to the microstructural texture remaining, however intergranular [59,110,323]. Figure 57a–c shows V-sample where some β-grains are cut from the crack propagation along the α grain boundaries then others can directly accommodate the crack propagation. Finally, the H-samples are subjected the crack propagation along the β-grains boundaries [59,295]. Zafari et al. [322] also showed deformed α′ plates through TEM analysis, which emphasizes entangled dislocations and dislocation cells (Figure 57d,e).

Finally, the SAEDP map shown in Figure 57f illustrates a randomly orientation of the α′ martensite.

Starting from the mechanical properties of the as-built samples, generally, the elongation increases at the expense of the tensile strengths as reported in Table 11, where the mechanical properties of heat-treated Ti6Al4V samples were illustrated. If the SR heat treatment slightly increases the elongation with a small loss in tensile strength, the ANN heat treatment confers good ductility satisfying the ASTM F2924-12 standard specification [32,59,230,271,318]. Generally, the heat treatments performed above the β-transus show a decreasing trend of the tensile strengths and an increase in ductility (Table 11).

As summarized in the same Table 12, the HTs effects are already significant as the temperatures reach T_0_, and then ~995 °C, where the cooling method and the residence time must be considered.

De facto, Etesami et al. [285] showed a significant increase of tensile strength despite the heat treatment at 930 °C × 2 h due to the followed WQ that induces the diffusionless α′-martensite transformation of the formed β-phase. Increasing the temperature above the β-transus, the recrystallization process (columnar → equiaxed β-grains) takes place only after adequate holding time and cooling method as previously discussed through the study conducted by [268]. Thus, a slow cooling rate increases tendentially the ductility values and decreases the tensile strengths, while a high cooling rate induces higher tensile strengths, but does not significantly improve the ductility (Table 11 and Table 12). Considering the HIP heat treatments, a very important reduction in the vol % of LOF surely increases the obtained tensile strengths and ductility due to the reduction in potential triggers of cracks [111,268,299,300]. On the other hand, considering a dense or fully dense as-built sample, the HIP heat treatment does not significantly improve the tensile properties compared to the effects induced by the same heat treatments without the use of pressure. In this scenario, Mahmud et al. [111] and Kasperovich et al. [303] show higher ductility combining the α + βHIP to the ANN heat treatment. These values were not even reached by the βHIP at 1050 °C × 2 h +100 MPa as proposed and analyzed by Leuders et al. [110]. The same authors showed that the tensile strength obtained after α + βHIP 920 °C × 2 h + 100 MPa are slightly higher than those obtained after βHIP 1050 °C × 2 h + 100 MPa despite an equal elongation (Table 11). A good balance between the strengths and ductility can be reached by performing a mixed heat treatment that induces a bi-modal microstructure (Section 6.2). De facto, Table 12 shows an increment of the tensile strengths, maintaining excellent elongations [112,224,265,305]. A good balance between the tensile strengths and the ductility was obtained in the research conducted by Sabban et al. [305]. The authors showed UTS > 1 GPa, YS > 850 MPa and the elongation higher than 16% after the cycling annealing (Table 11). Bai et al. [224], performing the same heat treatment with a holding time of 30′ for each step at 975 °C, showed higher UTS and YS values (1196 ± 10 MPa and 1054 ± 10 MPa, respectively) but an elongation of 9.8 ± 1.8%.

**Figure 58:**
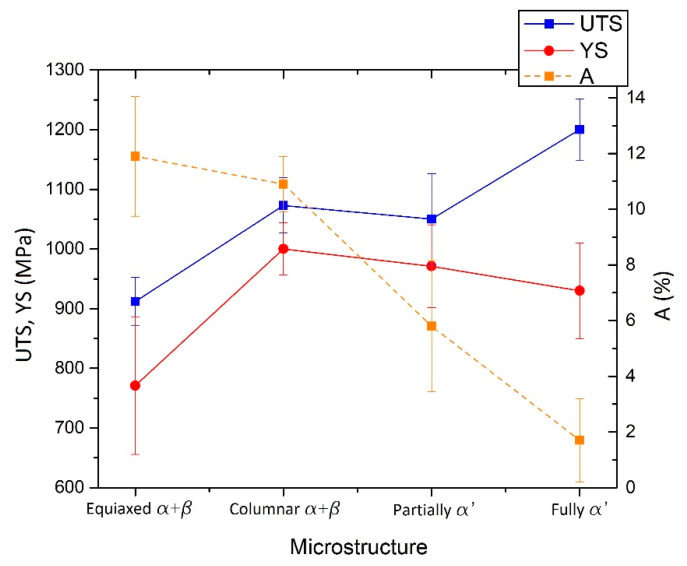
Trend of the UTS and YS values (blue and red lines) and ductility (orange dotted line) in relation to the Ti6Al4V microstructure obtained by the re-plotting of the results analyzed by Gallaraga et al. [30].

Gallaraga et al. [30] showed that mechanical properties variation resides in the microstructural changes after the different HTs (Table 10 and Table 11). De facto, the increase in strengths and the decrease in ductility (Figure 58) are related to the following microstructure: in relation to the different microstructure: equiaxed β-grains with α + β microstructure rather than columnar β-grains with α + β, partially or fully α′ martensite.

In this scenario, considering different samples having the same microstructure morphology, the tensile strength variation can be caused by the coarsening effects (see Section 6.2). Moreover, in this case, the Hall–Petch relationship (Equation (10)) can be considered to evaluate the strengthening mechanisms of the Ti6Al4V alloy in as-built and heat-treated conditions, respectively. Unlike the AlSi10Mg, the term d is the average of the α lamellae width in the lamellar microstructure, or the average grain size of the α-phase in the equiaxed microstructure [326]. Despite this, the yield strength decreases with the increasing width/grain size [30,327,328,329].

**Figure 59:**
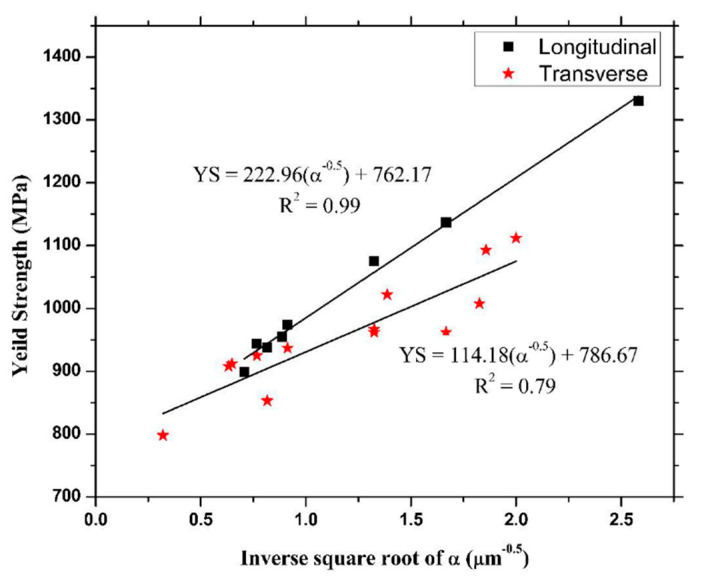
YS values versus the inverse square root of the α-lamella width for the H- and V-samples (Reprinted from reference [330]).

Considering the as-built case, Akram et al. [330] demonstrated the validity of the Hall–Petch equation also highlighting the mechanical properties anisotropy between the H- and V-samples. The same authors confirmed that H-samples are characterized by higher tensile strengths than the V-samples, highlighting a faster increase in the yield strength with the inverse square root of the α width (Figure 59).

The same graph shown in Figure 59 highlights R^2^ of 0.79 for the transverse samples (V-samples) indicating a greater values’ dispersion due to the microstructural texture and presence of defects previously discussed [330].

However, focusing on the heat-treated Ti6Al4V samples, which are characterized by a bi-modal microstructure, Galindo-Fernàndez et al. [327] proposed the variation of the Hall–Petch equation, as follows:

(20) $$\sigma_{HP}=\sigma_{0}+K\left(\frac{V_{\alpha_{s}}}{\sqrt{d_{\alpha }}}+\frac{1-V_{\alpha_{s}}}{\sqrt{d_{\alpha_{s}}}}\right)$$

where $V_{\alpha_{s}}$ (-) is the volume fraction of the secondary α_s_ phase, $d_{\alpha }$ and $d_{\alpha_{s}}$ (m) are the average grain size of the primary α_p_ and the secondary α_s_ phases, respectively. The same authors reported that the friction stress $\left(\sigma_{0},\;\left[\mathrm{MPa}\right]\right)$ must be considered for the α and the β phases unlike for the as-built Ti6Al4V samples, as follows:

(21) $$\sigma_{0}=\sigma_{0}^{\alpha }V_{\alpha }+\sigma_{0}^{\beta }\left(1-V_{\alpha }\right)$$

where $\sigma_{0}^{\alpha }$ and $\sigma_{0}^{\beta }$ (MPa) are the friction stress related to α and β phases, respectively. In addition, the Equation (21) can be rewritten as follows:

(22) $$\sigma_{0}=\left(\sigma_{prism}^{\alpha }+\sigma_{ss}^{\alpha }\right)V_{\alpha }+\sigma_{0}^{\beta }\left(1-V_{\alpha }\right)$$

where $\sigma_{prism}^{\alpha }$ is the stress required to activate the prismatic slip (~90 MPa), while $\sigma_{ss}^{\alpha }$ (MPa) is the solid solution strengthening stress defined as follows:

(23) $$\sigma_{ss}^{\alpha }=\sqrt[{3}]{{\left(\sum_{i}B_{i}^{3/2}x_{i}\right)}^{2}}$$

where $x_{i}$ is the concentration of the atoms of the element *i* and $B_{i}$ is its strengthening constant. Galindo-Fernàndez et al. [327] suggested $\sigma_{ss}^{\alpha }$ = 454 MPa and a friction stress of 544 MPa. At the same time, they concluded the following Equation (24) to describe the flow stress of the Ti6Al4V alloy:

(24) $$\sigma =\sigma_{0}\;\cdot \;G\left(T,\dot{\varepsilon }\right)+0.3M\mu b\sqrt{\rho }$$

where $G\left(T,\dot{\varepsilon }\right)$ is the normalized activation energy *G* for the cross-slip dislocation, which depends on the temperature *T* and on the strain rate ($\dot{\varepsilon }$), M is the Taylor factor (0.05 ÷ 3), μ is the shear modulus (μ=54−0.03T) GPa, b is the Burgers vector and ρ is the dislocations’ density. The fully martensitic microstructure leads to an increase in the yield strength of ~400 MPa due to the very small α′-martensite laths (Section 6.1), while the lamellar structure (~3 μm) and the equiaxed grains (~8 ÷ 10 μm) induced a contribution of ~170 and ~100 MPa, respectively.

**Figure 60:**
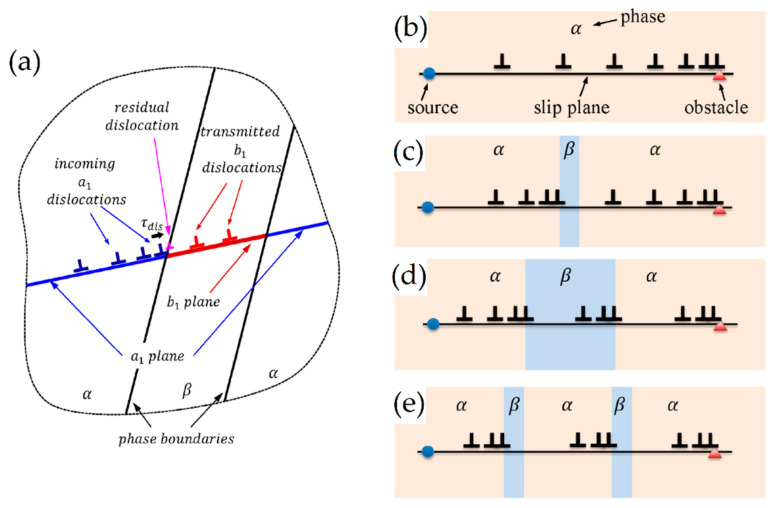
Schematic representation of the interaction between the dislocations and the α/β interface (**a**) during a plastic deformation emphasizing the effect of β laths: (**b**) pure α-phase, (**c**) one thin β-phase, (**d**) one thick β-phase, (**e**) two thin β-phase (Adapted from reference [331]).

In this scenario, the bimodal structure shows higher yield strength than the lamellar and equiaxed structures, respectively, due to its greater impediment to the dislocation movement thanks to the crystallographic misorientation of the primary α_p_ phase, secondary α_s_ phase and β phase [111,327]. The presence of the β-phase after the α′-martensite decomposition during the heat treatments (Section 6.2) induces the decrease in strength and varies the dislocation movement as reported in Figure 60 by Zheng et al. [331]. De facto, the β-phase is present between two adjacent α-lamellae (Figure 60) and can be considered as a barrier of the dislocations’ motion, which forms a pile-up generating a stress concentration [222,326,331]. In this scenario, Zheng et al. [331] and Kohn et al. [332] affirmed that the contribution of the β-phase was ignored by the Hall–Petch mechanism, considering that it was controlled by the platelets and/or laths of the α-phase.

In fact, the deformation initiated into α-grain, and with the subsequent strain hardening, the plastic flow starts into adjacent α-phase after the slip transfer across the β-phase that is an interface [326]. In this scenario, Zheng et al. [331] affirmed that due to the different Burgers vector between the α and β phases, a residual Burgers vector $\left(∆b=N\left(b^{\beta }-b^{\alpha }\right)\right)$ is left at the α/β interface generating a new residual dislocation (Figure 60). On the other hand, the amount of the β-phase and the α + β morphology induce significant effects on the mechanical behavior, as previously reported. Through Figure 60b–e, Zheng et al. [331] proposed a schematical representation of the dislocation pile-up variation induced by the amount and size of the β-phase.

**Figure 61:**
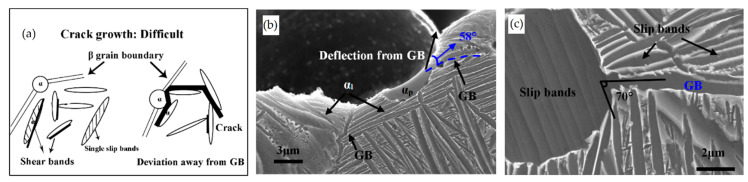
(**a**) Schematic representation of crack initiation and propagation into a trimodal microstructure; (**b**,**c**) SEM micrographs of the Ti6Al4V fracture surface where α_p_ and α_l_ indicate primary and lamellar α-phase, respectively (Adapted from reference [333]).

Finally, Tan et al. [333] reported an interesting point of view regarding the crack initiation and propagation into a bimodal and/or trimodal microstructure (Figure 61). In this scenario, the trimodal microstructure is a structure formed by globular primary α_p_ phase, lamellar α_p_ phase, and secondary α_s_ phase + β which is formed by the β-phase [334,335].

Generally, the crack nucleates at the primary α_p_ laths rather than at the boundaries of the β-grains and propagates tortuously because it is deflected by both the globular α_p_ phase and by the same β-grains boundaries. Tan et al. [333] affirmed that the trimodal microstructure or the bimodal microstructures with an appropriate amount of the α_p_ equiaxed phase may confer higher ductility than the lamellar micro-stretches. Moreover, a greater resistance of crack propagation is obtained if the globular α_p_ phase is at the β-grains boundaries. Figure 61c showed that α_p_ equiaxed phase is relatively soft and reaches yielding first due to the high amount of the slip band contained.

## 8. L-PBFed Ti6Al4V: The Corrosion Resistance

Moreover, in this case, the corrosion resistance is strongly correlated to the microstructural morphology and the vol% of the α and β phases, respectively, in other the aspects highlighted for the AlSi10Mg samples (Section 5) [209,336,337]. As reported in Section 6.1, the as-built Ti6Al4V samples are generally formed by a fully α′ martensitic microstructure, an SSS phase considered as a non-equilibrium phase enough to reduce the corrosion resistance [337]. De facto, the as-built Ti6Al4V samples showed lower corrosion resistance than the as-cast sample showing an α + β structure in the same corrosion environment [337]. In this scenario, Zhao et al. [338] affirmed that the as-built L-PBF samples showed a lower corrosion rate of the electron beam melted samples if the potential is lower than 1.2 V. The opposite results were highlighted with values > 1.5 V. The same authors explained this behavior through the different densities of the grain boundaries amount. The same results are obtained by [337].

As discussed for the mechanical properties, the corrosion resistance also varies in relation to the considered plane. Dai et al. [339] reported a slight variation between the xy and the xz planes, which showed 0.7 and 0.9 mg/cm^2^ of the weight loss, respectively, after 15 min in 1M HCl. On the other hand, the same samples are not corroded if the 3.5wt% of NaCl solution was used [340].

Focusing on the effects induced by the different heat treatments, Dai et al. [339] showed a decrease in the corrosion resistance with an increase in the corrosion current (i_corr_) from 0.9 μA/cm^2^ in as-built condition to values higher than 1.5 μA/cm^2^ after the heat treatment at 1000 °C × 2 h. This atypical correlation between the increase in the β-phases caused by the heat treatment (Section 6.2) and the decrease in the corrosion resistance were explained through the grain refinement. Chandramohan et al. [341] demonstrated that the 3.5 wt% of NaCl solution becomes corrosive on the heat-treated Ti6Al4V sample showing an increase in i_corr_ after the heat treatments both at 900 °C × 1 h and 1000 °C × 1 h. In the first case, the corrosion rate reaches 5.9 × 10^–4^ mm/y; while 3.4 × 10^−4^ mm/y in the second case. Finally, Pazhanivel et al. [342] showed an increase in E_corr_ (potential of corrosion) from −0.30 ± 0.02 to −0.20 ± 0.02 V in as-built and heat-treated condition at 850 °C × 2 h, respectively. The authors affirmed that the oxide film is more protective in heat-treated samples than in the as-built samples after the corrosion test in 3.5 wt% of NaCl due to the microstructural morphology. In fact, the homogeneous ultrafine α + β structure confers a higher number of nucleation sites of the passivation layer and, consequently, higher corrosion resistance than the inhomogeneous α′-martensite [342].

## 9. Conclusions

In the present paper, the effects induced by different heat treatments on as-built AlSi10Mg and Ti6Al4V samples produced via L-PBF were reviewed. From an industrial point of view, the wide range of applications makes heat treatment optimization necessary to obtain excellent mechanical properties through microstructural stabilization. Manufacturing high-quality, fully dense samples is a necessary requirement. Excellent mechanical performance characterizing as-built samples can be maintained even after heat treatment. The effects induced by both the process parameters and BP temperature on the microstructure, mechanical properties and fracture mechanisms of as-built samples have been reviewed. Systematically, the same effects have been analyzed after the following heat treatments: SR, ANNs, SHT + AA (T6 and STA), HIP.

In relation to AlSi10Mg samples manufactured via L-PBF, the main findings are reported as follows:

### 9.1. Microstructure and Corrosion Resistance of AlSi10Mg

1. The as-built microstructure is formed by a Si-eutectic network containing the α-Al matrix with Si particles, β-Al_5_FeSi intermetallics, GP zones and finely dispersed ε″/ε′ precipitates depending on the process parameters and/or BP temperatures.
2. Increasing the BP temperature leads to increased stress relaxation, the number of precipitates and their size. Such effects decrease with increasing distance from the pre-heated BP plate.
3. DA and SR: precipitation phenomena induced on as-built sample manufactured on a cold BP and Si-eutectic network destruction at T > 200 °C with Si particle coarsening.
4. SHT (+AA): Si particles coarsen with total Si-eutectic network destruction and microstructure recrystallization. This induces precipitation phenomena and β-Al_5_FeSi formation. T6 can increase the vol% of pores, unlike the HIP HT.
5. The corrosion resistance, which is characterized by an anisotropic mechanism in full and quasi-cellular structures, decreases from the as-built samples to the SR and T6 heat-treated samples due to both variations in the Si eutectic and precipitation phenomena.

### 9.2. Mechanical Properties of AlSi10Mg

6. Due to the strengthening phenomena conferred by this microstructure, the mechanical behavior is strongly anisotropic, exhibiting high tensile strength (UTS > ~400 MPa, YS > ~240 MPa) and low elongation (A < ~9%).
7. The post-HT tensile strength generally decreases while the ductility increases with increasing heat treatment temperatures. By increasing the DA temperatures above 255 °C, initiation of Si-eutectic network destruction induces a decrease in the tensile strength (UTS = ~340 MPa, YS = ~200 MPa) but also an increase in elongation with values higher than 10%. In this scenario, the anisotropic mechanical performance can be eliminated after the SR at 300 °C × 2 h.
8. The ductility can reach 23–29% after T6 and HIP HTs. Firstly, the subsequent AA to complete the T6 heat treatment recovers the tensile strength due to precipitation of the ε-Mg_2_Si (UTS = 230–330 MPa, YS = 180–280 MPa). Secondly, the tensile strength can be increased with the T6 rather than a DA after the HIP heat treatments.
9. In as-built samples and DA samples at low temperatures, the fracture mechanisms are dominated by both the laser scan tracks and MP boundaries, where the crack generally propagates (inter-layer and inter-track fractures). In other cases, the crack also propagates inside the center of the MP, generating a trans-track fracture. In relation to the SR at 300 °C and, therefore, after the T6 and HIP heat treatments, the fracture mechanisms are dominated by the Si-eutectic particles which generate the voids at their interface with the α-Al matrix. In this case, cracks can be deflected by the laser scan tracks and MP boundaries remaining after the heat treatment at high temperatures.

In relation to Ti6Al4V samples manufactured via L-PBF process, the main conclusions are outlined as follows:

### 9.3. Microstructure and Corrosion Resistance of Ti6Al4V

10. The as-built microstructure is formed by columnar β-grains arranged along the directions of the heat flux. In relation to the BP temperature, process parameters and the distance from the BP plane, columnar grains can contain a fully α′ microstructure (primary, secondary, tertiary and quartic α′-martensite’s), a mixture of α′ and α + β lamellar phase, or a fully ultrafine α + β microstructure.
11. Corrosion resistance is influenced by the β grain boundary density and is characterized by anisotropic behavior, increasing from the as-built state to samples formed by an α + β microstructure due to the higher number of nucleation sites where the passivation layer can form.
12. The heat-treated microstructure is progressively more influenced by heat treatment temperatures as the latter increase. Starting from ~400 °C, the α′ → α decomposition takes place inducing the diffusion of the Al and V, which cause β precipitation. At around 704–705 °C, the α′ → α + β transformation occurs, increasing drastically after the 800 °C up to the β-transus. Above the critical temperature, and into high-SSTR, the tensile strength can be recovered with appropriate cooling methods, remembering that the α-phase progresses towards globularization. These effects become progressively more important above the β-transus, where the recrystallization process from columnar to equiaxed grains also begins, depending on the time that the material is held at this temperature.

### 9.4. Mechanical Properties of Ti6Al4V

13. The UTS, YS (higher than 1.1 GPa and 950 MPa, respectively) and elongation (A < 9%) do not exhibit great differences between as-built samples formed by α′ martensite or ultrafine α + β microstructure.
14. The SR and ANN heat treatments performed in low-SSTR, which are not affected by the different cooling rate (WQ, AC, FC), induce slight coarsening effects that reflect on the tensile strength: UTS > 1 GPa, YS > 900 MPa and A < 10%. Between the medium- and high-SSTRs, coarsening effects increase and the α′ → α + β is completed. In addition, the cooling method becomes significant, reaching the T_0_. The UTS and YS decrease up to ~970 and ~900 MPa, respectively, after the ANN HT at 850 °C × 5 h (FC).
15. The ductility also increases up to 14%, and the tensile strengths decrease at values that can also satisfy the ASTM F2924-12 standard specification after HTs performed into high-SSTR.
16. The balance between the tensile strengths and elongation can be obtained with a bimodal and/or a trimodal microstructure after an appropriate combination of different ANN heat treatments below and/or above the β-transus.
17. The fracture mechanisms of the as-built and unrecrystallized samples are related, firstly, to the load conditions and columnar β-grain directions and, secondly, to the α-phase crystallographic orientations. This damaging behavior, together with the presence of LOF pores in the xy plane, induces significant anisotropic mechanical properties that can be reduced with the HIP heat treatment. In this case, the as-built samples transform their structure into α + β Widmanstätten, where the β-phase between two adjacent α lamellae modifies the fracture mechanism. Focusing on bimodal and/or trimodal microstructures, crack propagation follows the α-phase, and is finally characterized by a tortuous path due to the presence of the β-grains boundaries and globular α_p_ phase. The greatest effects are induced when this globular phase is located at β-grains boundary.

## 10. Future Trends and Prospective

AM processes are revolutionizing the industrial setting due to their important advantages in terms of sample geometry, customization and reduction in weight compared to conventional manufacturing processes. Reductions in production time and cost are also of fundamental importance. L-PBF makes up a large part of metal AM applications, where AlSi10Mg and Ti6Al4V cover the largest portion of demand in the aerospace, automotive and biomedical fields. For this reason, they are the most studied alloys in relation to this manufacturing process. AlSi10Mg and Ti6Al4V must guarantee the high requirements of these different applications, necessitating the manufacturing of excellent mechanical components and physical objects. Consequently, the effects induced by appropriate process parameters and HTs must be controlled to obtain the best results. 

For both lightweight alloys, there are many gaps in the optimization of the heat treatment parameters in relation to the microstructural morphology obtained by different L-PBF process parameters. Very few researchers have studied the effects of the BP temperature on as-built samples, for which the effects of heat treatment on the same samples are also largely unknown. These factors could potentially have important implications on the design of samples and their mechanical properties. Future studies must, therefore, define new heat treatments to optimize the mechanical properties of these alloys in order to preserve the metallurgical advantages conferred by the L-PBF process. Another area of interest is the mechanism involved during post-process heat treatments performed on Ti6Al4V alloy to improve mechanical properties. At the same time, more studies on cyclic heat treatments may be necessary.

## 11. Acronyms

Table 13 illustrates all acronyms and their definitions within the present review.

## Figures and Tables

**Table 1:** Principal process parameters values used to manufacture AlSi10Mg and Ti6Al4V samples via L-PBF process.

Materials	*P*, (W)	*v*, (mm/s)	*h*, (μm)	*t*, (μm)	*ED*, (J/mm^3^)	Fully Dense, Dense, Porous	BR ^1^, (cm^3^/h)	Ref.
AlSi10Mg	350	1050	170	50	39	Fully dense	32	[14]
	788	1099	300	60	40	Fully dense	71	[50]
	463, 625, 788, 950	500, 800, 1099, 1400, 1700, 2000	300, 350, 400		17–75	Dense	38–174	
	300, 463, 788	800, 1400, 1700, 2300	300, 350, 400			Porous		
	100	250, 500, 750, 1000	50, 100	40	25–200	Dense	2–14	[51]
	320, 360, 400	600, 750, 900	70.90–116.40	30	145–200	Dense	25–28	[52]
	370	1300	190	30	50	Dense	27	[53]
	240, 260, 320, 360, 400	1200, 1400, 1600, 1800, 2000	36, 40, 45, 51.4, 60	30	111	Fully dense	8–13	[54]
	400, 440	1350, 1500	105	50	38–56	Fully dense	25–28	[55]
	300	1230	105	50	68	Dense	23	
	150	500, 1500, 2500	45, 75, 105	30	74, 95, 222	Fully dense	2–28	[56]
	250	1500	75	30	74	Dense	12	
	150	2500	105, 150	30, 60	9, 13	Porous	28–81	
Ti6Al4V	250	1600	60	30	87	Fully dense	10	[32]
	170	1250	100	30	45	Dense	13	[57]
	200	200	180	50	111	Dense	6	[58]
	157	225	100	50	14	Dense	4	[59]
	100	700	75	30	6	Dense	6	[60]
Ti6Al4V	260, 280, 300	1000, 1200, 1400	140	30	44–67	Dense	15–21	[61]
	55–95	150–1000	49.5–99	25	148–269	Fully dense	1–11	[62]
	240, 300, 360	800, 1000, 1200	80, 100, 120	40	62.5–94	Dense	9–21	[63]
	200	800	80	30	10	Porous	7	[64]
	240	240	50	30	67	Porous	1	[65]
	100	400	70	50	7	Porous	5	[66]
	90	600	90	30	6	Porous	6	[67]

^1^ BR is the acronymous of the build rate.

**Table 2:** Defects present into L-PBFed samples: cause, remedy and effects.

Type of Defect	Cause	Remedy	Effects	Ref.
Lack-of-fusion (LOF)	Inhomogeneous distribution of the powder bed	Reduction in the layer thickness and increase of energy penetration	Decrease in mechanical properties and fatigue resistance	[51,59,70,71,72]
	Non-optimization of the *ED* function			
	Lack of material or low energy inducing no complete adherence of the melt to the surrounding material			
Keyhole pore	MP instability	Increase in energy depth penetration and laser power	Decrease in mechanical properties and fatigue resistance	[48,73]
	Non-optimized process parameters			
Gas pores	Gas dissolution within the melt material	Reduction in the layer thickness, and the pressure into the chamber Reduction in the O_2_ Re-melting	Loss density, decrease in tensile strength and fatigue resistance. Gas pores are less critical in crack propagation than the LOF	[51,74,75,76,77,78,79]
	Gas wrapped into the gas atomized particles			
	Entrapment of the gas present into the build chamber			
	Gas flow within the build chamber			
Anisotropy	Build orientation	HTs	Tensile properties correlated to the orientations	[59,80,81]
Preferential evaporation	Temperature-dependent vapor pressure	Reduction in linear energy density rather than the hatch spacing	Alloying elements loss and pore’s formation	[82,83,84]
Residual stress and distortion	High thermal gradient during the L-PBF process	Pre-heated BP Post-process HTs Opportune scan strategy and re-melting Sacrificial material and support structures	Sample distortion if residual stress is higher than the YS Alloying elements loss and pore’s formation Loss of tolerance requirement Reduction in fatigue resistance and tensile properties	[85,86,87,88]
Balling	Low viscosity of melt material	Reduction in *ED* value	Porosity	[62,89,90,91,92,93]
	Excess of melt material		Stress Concentration point	
	MP instability: capillarity, Marangoni’s effect		Surface quality and roughness	
	Splashing of MP due to its high surface temperature		Intralayer connection	

**Table 3:** Classification of the HTs analyzed in the present review for the L-PBFed AlSi10Mg samples.

HTs			Temperature Used	Scopes	Ref.
Direct aging	DA		T ≤ 200 °C	Si-eutectic network is not destroyed (T ≤ 200 °C). Alloying strengthening	[7,9,14,138,139,155,156,157,158]
Stress relief	SR		~300 °C	Avoid deformations during sample removal from the BP Residual stress reduction	[139,143,155,159,160,161,162,163]
Solution heat treatment	SHT	T6	400 < T_SHT_ < T_eutectic_^1^ T_SHT_ > 480 °C ^2^	Formation of SSS Melt of Si-eutectic (T > T_eutectic_) Increase of ductility	[7,143,157,162,163,164,165,166]
Artificial aging	AA		T_AA_ ≤ 200 °C 160 < T_AA_ < 180 °C ^2^	Alloying strengthening	
Hot Isostatic Pressing	HIP		500 °C < T < T_eutectic_	Sample’s densification Increase of ductility	[107,109,165,167,168]

^1^ 400 < T < 480 ÷ 500 °C can be considered as an annealing (ANN) HT. ^2^ According to the ASTM F3318-18 [166].

**Table 4:** Mechanical properties of as-built and heat-treated L-PBFed AlSi10Mg samples.

Process Parameters					Directions	HT		UTS (MPa)	YS (MPa)	A (%)	Ref.
*P* (W)	*v* (mm/s)	*h* (μm)	*t* (μm)	BP							
370	1400	70	90	150 °C	H	As-built		441 ± 3	285 ± 6	6.6 ± 0.8	[9]
								411 ± 9 ^1^	237 ± 6	7.0 ± 1.3	
750	1100	--	--	RT	--	As-built		375 ± 18	225 ± 14	6.0 ± 2.5	[13]
350	1150	170	50	150 °C	H	As-built		430 ± 8	286 ± 8	7.0 ± 0.4	[14]
								365 ± 7 ^1^	220 ± 2	7.2 ± 0.3	
--	--	190	60	--	H	As-built		323 ± 2	190 ± 6	6.7 ± 0.2	[137]
			30					367 ± 4	244 ± 1	6.9 ± 1.0	
		100	30					469 ± 4	314 ± 1	5.6 ± 0.6	
		190	60		V			340 ± 1	214 ± 6	3.2 ± 0.1	
			30					380 ± 2	233 ± 2	3.9 ± 0.2	
		100	30					437 ± 4	278 ± 1	3.4 ± 0.1	
--	--	--	60	RT	--	As-built		435	250	7.5 ± 2.5	[139]
				200 °C				310	160	5.0 ± 1.0	
370	1300	190	30	165 °C	H	As-built		429 ± 8	226 ± 7	4.0 ± 0.3	[143]
					V			418 ± 7	269 ± 6	7.8 ± 0.4	
340	1300	200	30	160 °C	H	As-built		386 ± 3	248 ± 2	8.6 ± 1.4	[138]
					V			412 ± 5	228 ± 4	7.0 ± 0.1	
250	1400	130	30	--	--	As-built		448	264	9.8	[158]
300	1000	130	40	--	--	As-built		463 ± 3	237 ± 4	7.6 ± 1.0	[165]
350	1140	170	50	100 °C	--	As-built		434 ± 12	322 ± 8	5.3 ± 0.2	[170]
200	1000	150	30	--	V	As-built		465 ± 8	305 ± 4	8.6 ± 1.4	[176]
--	--	--	--	--	H	As-built		318	216	5.7	[177]
					V			320	221	5.4	
370	1300	190	30	--	H	As-built		409 ± 2	242 ±2	10.9 ± 0.7	[178]
					V			410 ± 2	224 ± 1	6.7 ± 0.3	
390	1300	190	30	RT	H	As-built		525 ± 4	287± 2	--	[179]
350	1150	170	50	150 °C	V	As-built		393 ± 20	273 ± 3	2.5 ± 0.4	[180]
370	1400	70	90	150 °C	H	DA	200 °C × 4 h	374 ± 1	231 ± 2	8.2 ± 1.2	[9]
								363 ± 8 ^1^	219 ± 6	8.5 ± 0.6	
350	1150	170	50	150 °C				371 ± 9	230 ± 8	8.2 ± 0.7	
								348 ± 2 ^1^	209 ± 2	7.6 ± 0.8	
750	1100	--	--	RT	--	DA	160 °C × 8 h	399 ± 13	284 ± 16	4.5 ± 1.9	[13]
350	1150	170	50	150 °C	H	DA	175 °C × 6 h	419 ± 16	258 ± 9	7.6 ± 0.4	[14]
								396 ± 18 ^1^	232 ± 11	7.6 ± 0.6	
						DA	200 °C × 6 h	395 ± 11	235 ± 12	9.0 ± 1.0	
								350 ± 9 ^1^	199 ± 6	9.2 ± 0.5	
						DA	225 °C × 6 h	341 ± 15	199 ± 6	13.6 ± 1.1	
								331 ± 19 ^1^	184 ± 5	15.2 ± 1.0	
--	--	--	60	RT	--	DA	170 °C × 6 h	400	295	5.05 ± 0.5	[139]
340	1300	200	30	RT	H	DA	160 °C × 4 h	471 ± 1	321 ± 2	8.6 ± 0.5	[138]
					V			493 ± 1	292 ± 1	6.0 ± 0.6	
250	1400	130	30	--	--	DA	180 °C × 6 h	452	310	6.2	[158]
300	1000	130	40	--	--	DA1	177 °C × 10 h	418 ± 9	233 ± 12	5.1 ± 0.8	[165]
						DA2	177 °C × 100 h	403 ± 9	229 ± 12	4.2 ± 0.6	
						DA3	177 °C × 1000 h	391 ± 5	231 ± 9	4.6 ± 0.7	
750	1000	--	--	RT	--	SR	300 °C × 2 h	225 ± 7	132 ± 9	11.5 ± 3.5	[13]
370	1300	190	30	RT	--	SR	300 °C × 2 h	302 ± 15	210 ± 16	10.7 ± 1.6	[107]
--	--	--	60	RT	--	SR	270 °C × 2 h	335	210	12 ± 2	[139]
370	1300	190	30	165	H	SR	300 °C × 2 h	257 ± 1	160 ± 1	18.1 ± 0.5	[143]
					V			261 ± 3	170 ± 2	19.1 ± 1.0	
300	1000	130	40	--	--	SR	285 °C × 2 h	249 ± 10	153 ± 8	21.3 ± 1.7	[165]
						SR+DA1	285 °C × 2 h + 177 °C × 10 h	246 ± 9	154 ± 8	21.6 ± 1.8	
						SR+DA2	285 °C × 2 h + 177 °C × 100 h	271 ± 4	174 ± 3	16.5 ± 1.2	
						SR+DA3	285 °C × 2 h + 177 °C × 1000 h	245 ± 5	155 ± 2	14.8 ± 2.0	
						SR	190 °C × 2 h ^2^	443 ± 16	258 ± 4	4.7 ± 1.2	
						SR + DA1	190 °C × 2 h + 177 °C × 10 h	441 ± 9	231 ± 7	5.0 ± 0.8	
						SR + DA2	190 °C × 2 h + 177 °C × 100 h	407 ± 8	229 ± 7	5.0 ± 0.7	
						SR + DA3	190 °C × 2 h + 177 °C × 1000 h	387 ± 4	221 ± 6	5.3 ± 1.1	
200	1000	150	30	--	V	SR	300 °C × 4 min	322 ± 5	220 ± 4	6.3 ± 0.2	[176]
						SR	300 °C × 40 min	282 ± 4	192 ± 6	12.8 ± 0.7	
390	1300	190	30	RT	H	SR	250 °C × 2 h	421 ± 2	249 ± 2	--	[179]
						SR	300 °C × 2 h	341 ± 16	212 ± 12	--	
--	--	--	--	200 °C	H	SR	300 °C × 2 h	327 ± 3	209 ± 1	--	[181]
					V			350 ± 0	209 ± 0	--	
370	1400	70	90	150 °C	H	T6	505 °C × 4 h + 175 °C × 4 h	295 ± 2	239 ± 2	11.3 ± 2.5	[9]
								292 ± 5 ^1^	236 ± 5	9.5 ± 2.2	
750	1100	--	--	RT	--	SR + T6	300 °C × 2 h + 543 °C × 1 h + 180 °C × 1 2h	329 ± 12	278 V 6	6.0 ± 1.4	[13]
							300 °C × 2 h + 543 °C × 3 h + 180 °C × 8 h	332 ± 11	292 ± 12	4.4 ± 0.8	
350	1150	170	50	150 °C	H	T6	505 °C × 4 h + 175 °C × 4 h	274 ± 3	226 ± 2	8.4 ± 0.8	[14]
								290 ± 4 ^1^	238 ± 4	9.7 ± 1.0	
370	1300	190	30	RT	--	T6	540 °C × 2 h	297 ± 10	234 ± 7	5.0 ± 1.5	[107]
--	--	--	--	RT	--	T6	540 °C × 8 h + 160 °C × 6 h	225	180	--	[139]
370	1300	190	30	165 °C	H	SHT	500 °C × 2 h	133 ± 1	78 ± 1	29 ± 1	[143]
340	1300	200	30	RT	H	T6	540 °C × 1 h + 160 °C × 4 h	323 ± 0	243 ± 0	15.3 ± 2.4	[138]
					V			302 ± 2	223 ± 3	16.0 ± 1.4	
300	1000	130	40	--	--	T6	530 °C × 6 h	308 ± 8	240 ± 8	16.2 ± 1.5	[165]
						T6 + DA1	530 °C × 6 h + 160 °C × 6 h + 177 °C × 10 h	283 ± 10	232 ± 7	14.5 ± 1.5	
						T6 + DA2	530 °C × 6 h + 160 °C × 6 h + 177 °C × 100 h	201 ± 2	159 ± 3	16.2 ± 0.6	
						T6 + DA3	530 °C × 6 h + 160 °C × 6 h + 177 °C × 1000 h	144 ± 6	94 ± 4	28.9 ± 2.7	
350	1140	170	50	100 °C	--	SHT	450 °C × 2 h	282 ± 6	197 ± 4	13.4 ± 0.5	[170]
							500 °C × 2 h	214 ± 5	126 ± 2	23.5 ± 0.8	
							550 °C × 2 h	168 ± 2	91 ± 2	23.7 ± 0.8	
200	1000	150	30	--	V	SHT	540 °C × 2 h	185 ± 7	98 ± 2	16.7 ± 0.5	[176]
						T6	540 °C × 2 h + 160 °C × 2 h	254 ± 7	194 ± 5	7.0 ± 0.3	
250	1400	130	30	--	--	T6	520 °C × 2 h + 180 °C × 6 h	242	180	9.6	[180]
--	--	--	--	200 °C	H	SR + T6	300 °C × 2 h + 540 °C × 8 h + 160 °C × 10 h	337 ± 8	280 ± 5	--	[181]
					V			315 ± 15	267 ± 12	--	
370	1300	190	30	RT	--	HIP	500 °C × 75 min + 100 MPa	176 ± 2	108 ± 3	25.0 ± 0.5	[107]
						SR +HIP + T6	300 °C × 2 h + 500 °C × 75 min + 100 MPa + 540 °C × 2 h + 180 °C × 4 h	345 ± 1	308 ± 25	5.8 ± 1.7	
						SR + HIP + T6	300 °C × 2 h + 500 °C × 75 min + 100 MPa + 540 °C × 2 h + 180 °C × 12 h	306 ± 9	254 ± 9	8.7 ± 3.3	
300	1000	130	40	--	--	HIP	515 °C × 3 h + 100 MPa	144 ± 1	88 ± 4	32.2 ± 1.2	[165]
300	1000	130	40	--	--	HIP + DA1	515 °C × 3 h + 100 MPa + 177 °C × 10 h	144 ± 1	93 ± 2	31.8 V 1.0	[165]
						HIP + DA2	515 °C × 3 h + 100 MPa + 177 °C × 100 h	138 ± 1	93 ± 1	30.5 ± 3.1	
						HIP+DA3	515 °C × 3 h + 100 MPa + 177 °C × 1000 h	127 ± 1	80 ± 1	33.3 ± 1.3	

^1^ The UTS, YS and A are related to the top samples (maximum distance from the pre-heated BP) [14]. ^2^ HT defined as SR by the same authors [165].

**Table 5:** Review on the corrosion of the AlSi10Mg samples.

Environment	Corrosion Related to	HT	Corrosion Characteristics		Ref.
Harrison’s solution	Surface finish, build orientation	As built	Anisotropy of corrosion resistance between the xz and xy planes	E_corr_ = −0.6 ÷ −0.7 V	[193]
Harrison’s solution	Surface finish and HTs	As built	The spontaneously passive layer formed in air is more protective than the same forming during the L-PBF process.	E_corr/unpolished_ = −0.561 ÷ −0.649 V E_corr/polished_ = −0.570 ÷ −0.758 V	[194]
		275 °C × 2 h (AC)	SR does not reduce the susceptibility to the corrosion attack penetration.	E_corr/unpolished_ = −0.615 ÷ −0.869 V E_corr/polished_ = −0.476 ÷ −0.624 V	
		275 °C × 2 h (AC) + 525 °C × 1 h (WQ)	SR + SHT increase the local corrosion	E_corr/unpolished_ = −0.600 ÷ −0.608 V E_corr/polished_ = −0.610 ÷ −0.620 V	
01M NaCl	Scans strategy, layer thickness	As-built (BP T = 180 °C)	Pitting into MPBs, crack formation. Relation between the cellular grain size and the Volta potential between Si and α-Al.	E_corr_ = −0.639 ÷ −0.650 V	[197]
3.5 wt% NaCl	SLM/As cast	As built	As built lower mass loss than as cast	E_corr_ = −0.73 V, i_corr_ = 0.54 μA/cm^2^	[199]
3.5 wt% NaCl	Surface finish	300 °C × 2 h	Higher resistance for the polished than unpolished samples	Unpolished 2.13 pit/cm^2^. Polished 0.93 pit/cm^2^	[200]

**Table 6:** As-built microstructure of Ti6Al4V samples manufactured through different *ED* values.

Sample	*ED* (J/mm^3^)	Microstructure	Ref.
S1	68.47	Acicular α′-martensite	[221]
S3	50.62	Lamellar α + β	
S7	33.74	Acicular α′-martensite + minority of α + β	

**Table 7:** Lattice parameters of the α and α′ phases into L-PBFed Ti6Al4V sample.

Phases	a (Å)	c (Å)	c/a	Ref.
α-phase	0.295	0.468	1.5896	[243]
	0.294	0.467	1.588	[254]
	0.293	0.468	1.597	[255]
α′-martensite	0.293	0.468	1.597	[227]
	0.293	0.467	1.594	[256]

**Table 8:** Classification of the heat treatments analyzed in the present review for the L-PBFed Ti6Al4V samples.

HTs			Temperature Used	Scopes	Ref.
Stress Relief	SR		400 < T_SR_ < 800 °C ^1^	Residual stress reduction Mechanical properties improvement Avoid distortions	[54,59,111,265,266,267,268,269,270]
Annealing	ANN		700 < T_ANN_ < 940 °C	Martensite decomposition Mechanical properties improvement (balance between strength and ductility: bi-modal structure)	[226,230,245,259,260,271]
SHT	SHT	STA	940 < T_SHT_ < 970 °C ^2^ T^β^_SHT_ > β-transus	Microstructural variation to improve ductility	[30,271,272,273,274,275,276]
Artificial aging	AA		T_AA_ ≤ 700 °C ^3^	Higher mechanical properties than the ANNed samples. α, β, α_2_-Ti_3_Al precipitation	
Hot Isostatic Pressing	HIP		900 < T_SHT_ < 1050 °C (p ~ 100 MPa) ^4^	Sample’s densification Increase in ductility and fatigue strength Improvement of biocompatibility	[110,111,269,276,277,278,279]

^1^ According to the ASTM F3310-18 [269]. ^2^ According to H-81200C specification [280]. ^3^ Overagin conditions. ^4^ In some cases, the pressure used is 150 ÷ 200 MPa.

**Table 9:** Effects of different HTs on Ti6Al4V microstructure.

HT	Cooling Method	Microstructure	Ref.
500 °C × 10 h	FC	α′-martensite decomposition	[227]
T < 600 °C	WQ	No morphological changes of the as-built microstructure	[233]
		α′-martensite decomposition in α platelet arranged in the same martensite orientation	
640–650 °C	-	Nucleation of β phase	[284]
700 °C × 2 h	FC	Partially α′-martensite decomposition	[212]
		Fine α needle-like phase	
700 °C × 2 h	FC	α′ → α + β transformation	[230]
		Small fraction of β nano-particles	
704 °C × 2 h	FC	α′ → α + β transformation	[268]
730 °C × 2 h	FC	α′ → α + β transformation and grain growth	[59]
		α′ and α phases with the same length and width size	
750 °C × 2 h	WQ	α′ → α decomposition (same directions)	[233]
750 °C × 8 h	AC	α′ → α + β transformation (quartic α′ → β; and primary, secondary and tertiary α′ → α)	[245]
		Minimal grain growth	
750 °C × 10 h	FC	α′ → α + β fully transformed	[227]
800 °C × 2 h	FC	α′ → α + β transformation does not reach the equilibrium Solute distribution into the matrix α′, α and β microstructure	[230]
800 °C × 6 h	FC	Twins disappeared + recovered microstructure	
800 °C × 10/20 min	AC	10 min does not affected the microstructure, while 20 min induces α′ decomposition	[265]
800 °C × 4 h	AC	Complete decomposition of α′ martensite	
		Regular arrangement of α + β structure caused by self-accommodation α′	
850 °C × 1 h	FC	α′ → α decomposition and β formation due to the V content α′, α and β microstructure	[270]
910 °C × 0.5/2/8 h	WQ	Primary α′ → α lamella in β matrix, tertiary and quartic α′ into β 0.5 h is not sufficient to obtain equilibrium phases which are obtained after 2 h	[245]
945 °C × 4 h	WQ	Large amount of grain growth	
960 °C × 0.5/4 h	WQ	0.5 h is similar to the 910 °C × 0.5 h (WQ). Globularisation of the α phase and significant grain growth. The α phase becomes elongated.	
900 °C × 2 h	AC	α′ → α + β transformation with lamellar morphology	[285]
		Heterogeneous distribution of alloying elements + secondary α	
950 °C × 2 h	FC	α′ → α + β lamella more stable and α′ is fully decomposed	[265]
		β grains become equiaxed	
950 °C × 1 h	WQ	Primary α coarsened	[275]
		Precipitate dissolve and a SSS is formed	
950 °C × 1 h	AC	β → α″ transformation	
		Grain growth, but finer than FC Bi-lamella structure with secondary α + β nano-particles	
950 °C × 1 h	FC	Greater diffusion-controlled nucleation	
1000 °C	WQ	Recrystallization (β equiaxed grain) + α′ martensite Colony-tipe α′ martensite → weave-type α′ martensite	[212]
1000 °C × 2 h	FC	α′ → α + β lamellae stable transformation	
1015 °C × 15 min	WQ	α′ martensite into columnar β grains	[268]
1015 °C × 15 min	AC	α′ coarse needle + α coarse phase	
1015 °C × 2 h	FC	α + β lamella	[32]
1050 °C × 1 h	FC	Recrystallization (β equiaxed grain) + α crystalizes in the β grain boundaries α + β structure	[270]
1050 °C × 1 h	AC	Recrystallization (equiaxed + half equiaxed β grains)	[265]
1150 °C × 2 h	AC	Recrystallization (β equiaxed grain) + α crystalizes in the β grain boundaries α + β coarsened lamella structure	[285]
920 °C × 2 h + 100 MPa + 920 °C × 12 h	AC + AC ^1^	α′ → α + β equilibrium lamellar mixture without a significant dislocation density	[275]
704 °C × 2 h + 920 °C × 2 h + 100 MPa	FC + AC	α′ → α + β equilibrium lamellar mixture	[268]
955 °C × 1 h + 600 °C× 8 h	FC+WQ ^2^ + AC	α + β lamellar microstructure into semi-equiaxed grains	[286]
850 °C × 2 h + (975 °C × 30 min → 875 °C → 975 °C × 30 min) ^3^	FC + AC	α + β lamellar microstructure (width of α laths increases with temperature) Large primary + fine secondary α phases	[224]
950 °C × 2 h + (975 °C × 30 min → 875 °C → 975 °C × 30 min) ^3^	FC + AC	α + β lamellar microstructure (width of α laths increases with temperature) Large primary + fine secondary α phases	[224]
1020 °C × 2h + (975 °C × 30 min → 875 °C → 975 °C × 30 min) ^3^	FC + AC		
950 °C × 15 min + 500 °C × 8 h	WQ + AC	Acicular α′ + primary rod-like α phase	[287]
950 °C × 4 h + 150 MPa	--	Coarsened α + β lamellar	[288]
900 °C × 2 h + 100 MPa	FC	α + β lamellar	[223]
920 °C × 2 h + 100 MPa	--	α + β lamellar and recrystallization process	[110]
1050 °C × 2 h + 100 MPa		α + β lamellar more coarsened than 900 °C × 2 h	

^1^ Controlled air cooling with a cooling rate of 0.02 °C/s [111]. ^2^ FC between 955 and 855 °C and WQ at room temperature [275]. ^3^ cyclic annealing (C-ANN) to obtain a bi-modal structure [224].

**Table 11:** Mechanical properties of the heat-treated Ti6Al4V samples manufactured via L-PBF process.

HT Region	HT		Microstructure ^1^	Directions	E (GPa)	UTS (MPa)	YS (MPa)	A (%)	Ref.
α + β	540 °C × 5 h (WQ)	SR	--	V	113 ± 30	1223 ± 52	1118 ± 39	5.4 ± 2.0	[32]
	611 °C × 2 h (AC)	SR	α′ martensite + β precipitate	V	--	1213 ± 20	1171 ± 40	13.3 ± 0.7	[285]
	640 °C × 2 h	ANN ^2^	β(C)			1225 ± 4	1104 ± 8	7.4 ± 1.6	[319]
						1214 ± 24	1140 ± 43	3.2 ± 2.0	
						1256 ± 9	1152 ± 11	3.9 ± 1.2	
	650 °C × 3 h (FC)	SR	α′ martensite + α, β precipitation in β(C)	V	111 ± 1	1101 ± 5	1040 ± 7	7.8 ± 0.7	[271]
	670 °C × 5 h (FC)	SR	Acicular α′ + α + β in β(C)	H(xz)	116 ± 1	1170 ± 5	1112 ± 4	9.2 ± 0.2	[111]
				H(xy)	115 ± 1	1207 ± 5	1147 ± 7	7.9 ± 0.6	
				V	121 ± 1	1193 ± 8	1164 ± 5	3.8 ± 0.1	
	700 °C × 2 h (FC)	SR	α′ + α + β	H	--	1109 ± 18	1013 ± 17	13.5 ± 0.2	[230]
	700 °C × 1 h (10 K/min)	SR	α′ + α (fine needles) + β in β(C)	--	117.45	1115	1051	11.3	[304]
	705 °C × 3 h (AC)	ANN	β(C)	V	115 ± 2	1082 ± 34	1026 ± 35	9.1 ± 2.0	[32]
	730 °C × 2 h (AC)	ANN	α′ + α + β in β(C)	H(xz)	113 ± 9	1057 ± 8	958 ± 6	12.4 ± 0.7	[59]
α + β	730 °C × 2 h (AC)	ANN	α′ + α + β in β(C)	H(xy)	112 ± 6	1065 ± 21	974 ± 7	7.0 ± 0.5	[59]
				V	117 ± 6	1052 ± 11	937 ± 9	9.6 ± 0.9	
	730 °C × 2 h (AC)	ANN		H	101 ± 4	1046 ± 6	965 ± 16	9.5 ± 1	[325]
				V	110 ± 29	1000 ± 53	900± 101	1.9 ±8 0.8	
	800 °C × 2 h (AC)	ANN	α′ + α (0.7μm) + β in β(C)	--	--	1073 ± 9	1010 ± 11	17.0 ± 1.0	[265]
	800 °C × 2 h	SR	β(C)	V	--	1228 ± 32	--	8.0 ± 1.5	[110]
	800 °C × 2 h (FC)	ANN	α + β lamellae	H	--	1024 ± 10	955 ± 9	14.7 ± 0.4	[230]
	800 °C × 6 h (FC)	ANN		H	--	1017 ± 5	928 ± 6	18.9 ± 1.3	
	800 °C × 12 h (FC)	ANN		H	--	1007 ± 4	923 ± 3	18.5 ± 0.6	
	850 °C × 1 h (FC)	ANN	α′ + α + β in β(C)	V	114 ± 1	1003 ± 4	945 ± 6	8.1 ± 0.3	[271]
	850 °C × 2 h (FC)	ANN	α + β with increase of β fraction in β(C)	V	115 ± 4	1004 ± 6	955 ± 6	12.8 ± 1.4	[32]
	850 °C × 5 h (FC)	ANN	α + β with increase of β fraction in β(C)	V	112 ± 3	965 ± 20	909 ± 24	--	
	900 °C × 30′ (AC)	ANN	α + β	V	--	1013 ± 23	981 ± 26	16.1 ± 2.9	[285]
	900 °C × 60′ (AC)	ANN	α + β	V	--	1026 ± 8	974 ± 1	15.5 ± 0.6	
	900 °C × 120′ (AC)	ANN	α + β	V	--	1021 ± 18	975 ± 16	15.0 ± 0.2	
	920 °C × 2 h + 100 MPa	HIP	β(E)	V	--	1086 ± 26	--	13.8 ± 1.3	[110]
	930 °C × 2 h (FC)	ANN	α + β	V	--	968 ± 4	924 ± 8	19.5 ± 3.2	[285]
	930 °C × 2 h (AC)	ANN			--	1031 ± 44	991 ± 47	16.9 ± 1.9	
	930 °C × 2 h (WQ)	ANN			--	1097 ± 12	1048 ± 15	9.2 ± 1.0	
	940 °C × 1 h (AC) + 650 °C × 2 h (AC)	STA	α + β in β(C)	V	116 ± 3	948 ± 27	899 ± 27	13.6 ± 0.3	[32]
	950 °C × 2 h (AC)	ANN	Bi-lamellar structure α (2.4 μm) + β in β(C)	--	--	945 ± 5	893 ± 3	14.1 ± 1.5	[265]
	950 °C × 1 h (FC)	ANN	α′ + α + β coarsen in β(C→E)	V	114 ± 2	926 ± 3	860 ± 5	10.5 ± 0.6	[271]
	950 °C × 1 h (WQ) + 700 °C × 2h(AC)	Mixed	α + β	H	103 ± 11	1036 ± 30	944 ± 8	8.5 ± 1	[325]
				V	98 ± 3	1040 ± 4	924 ± 14	7.5 ± 2	
	900 °C × 2 h + 700 °C × 1 h (10 K/min)	Mixed	α/β phase columnar in β(C)	--	118.8	988	980	9.5	[303]
	910 °C × 8 h (WQ) + 750 °C × 4 h (FC)	Mixed	Bimodal microstructure	--	--	~950	~900	~18	[245]
	900 °C × 2 h + 100 MPa + 700 °C × 1 h (10 K/min)	HIP + ANN	α/β phase columnar in β(C)	--	115.4	973 ± 1	885 ± 3	19.0 ± 0.5	[303]
	920 °C × 2 h(AC) + 100 MPa + 920 °C × 12 h (AC)	HIP + ANN	Basketweave α + β in β(E)	H(xz)	116 ± 1	1007 ± 1	937 ± 1	16.0 ± 0.4	[111]
				H(xy)	117 ± 2	1003 ± 1	936 ± 3	15.4 ± 0.3	
				V	113 ± 1	999 ± 1	911 ± 4	16.9 ± 0.9	
	920 °C × 2 h + 100 MPa		β(E)	--	--	1089 ± 26	--	13.8 ± 1.3	[110]
	See Table 9	C ANN	Bimodal microstructure	H(xy)	--	1017 ± 16	865 ± 19	18 ± 1	[305]
				V	--	1004 ± 23	849 ± 12	16 ± 1	
α + β	940 °C × 1 h + 650 °C × 2 h (AC)	STA	--	V	116 ± 2	948 ± 27	899 ± 27	13.6 ± 0.3	[32]
β	1020 °C × 2 h (FC)	SHT	α + β + α_2_-Ti_3_Al in β(E)	V	--	840 ± 27	760 ± 19	14.1 ± 2.5	[32]
	1050 °C × 1 h (FC)	SHT	α + β + α_p_ along β grain boundaries β(E)	V	114 ± 1	869 ± 3	787 ± 4	11.5 ± 1.0	[241]
	1050 °C × 1 h (WQ) + 820 °C × 2 h (AC)	SHT + ANN	α + β	H	96.7 ± 5	1019 ± 11	913 ± 7	8.9 ± 1.0	[325]
				V	95 ± 4	951 ± 55	869 ± 64	7.9 ± 2.0	
	1050 °C × 2 h	SHT	β(C)	V	--	986 ± 45	--	13.8 ± 0.8	[110]
	1050 °C × 2 h + 100 MPa	HIP	β(E)	V	--	1007 ± 15	--	13.5 ± 0.7	
	1050 °C × 1 h (AC)	SHT	α (0.7μm) +β in β(E, hE)	--	--	988 ± 8	869 ± 4	13.4 ± 0.7	[265]
	1050 °C × 1 h (WQ) + 990 °C × 30′ (AC)	SHT + ANN	Basketweave α + β in β(E, hE)	--	--	962 ± 12	838 ± 6	12.0 ± 0.1	
	1150 °C × 2 h (AC)	SHT	α + β coarsen + α grain boundaries	V	--	1128 ± 8	1107 ± 10	4.9 ±	[285]
β	1200 °C × 1 h (AC)	SHT	α (0.9μm) + β in β(E, hE)	--	--	988 ± 8	878 ± 7	11.2 ± 1.2	[265]
β + α + β	1015 °C × 30′ (AC) + 843 °C × 2 h (FC)	SHT + ANN	β(C)	V	--	874 ± 23	801 ± 20	13.5 ± 1.2	[32]
	1015 °C × 30′ (AC) + 730 °C × 2 h (AC)	STA	β(E)	V	113 ± 3	902 ± 19	822 ± 25	12.7 ± 0.6	

^1^ C, E and hE indicate Columnar, Equiaxed and half-Equiaxed β-grains. ^2^ the authors defined this heat treatment as ANN highlighting however the effects induce by the SR heat treatment.

**Table 12:** Effects induced on microstructure and tensile properties by the different heat treatment temperatures.

HT Region		Microstructural	Strength	Ductility
α + β	T < low-SSTR	α′ → α decomposition Slight coarsening (high residence time) No effects of the cooling rate Ti_3_Al precipitation	~as-built	
	low- < T < medium-SSTR	α′ → α + β decomposition (~705 °C) Slight coarsening No effects of the cooling rate	↓	↑
	medium- < T < high-SSTR	α + β microstructure + α globularization Coarsening effects Effects of cooling tare (T → T_0_)	↓	↑↑
β	T > $T_{\beta_{\mathrm{Tr}}}$	Recrystallization process (C → E) Coarsening effects Effects of cooling method	↓↓	↑↑

**Table 13:** Acronyms used in the present review and their definitions.

Acronyms	Meanings
A	Elongation at break (%)
AA	Artificial Aging
AC	Air Cooling
AM	Additive Manufacturing
ANN	Annealing
BFTEM	Bright-Field Transmission Electron Microscopy
BP	Build Platform
BR	Build Rate
C-ANN	Cycling Annealing
CP	Cold Platform
DA	Direct Aging
DED	Direct Energy Deposition
DSC	Differential Scanning Calorimetry
ED	Energy Density
EBSD	Electron Backscatter Diffraction
FC	Furnace Cooling
H	Horizontal
HAADF	High-Angle Annular Darf-Field
HAZ	Heat Affected Zone
HIP	Hot Isostatic Pressing
HP	Hot Platform
HRTEM	High-Resolution Transmission Electron Microscopy
HT	Heat Treatment(s)
L-PBF(ed)	Laser-Powder Bed Fusion/(Fused)
LSW	Lifshitz, Slyozov, Wagner
LOF	Lack-Of-Fusion
M_s_, M_f_	Martensite start, finish
MP	Melt/Molten pool
MPB	MP Boundary(s)
MPC	MP Center(s)
OM	Optical Microscope
PBF	Powder Bed Fusion
SAED	Selected Area Electron Diffraction
SCC	Stress Corrosion Cracking
SEM	Scanning Electron Microscopy
SDAS	Secondary Dendrite Arm Spacing
SHT	Solution Heat Treatment
SLM	Selective Laser Melting
SSTR	Solid Solution Temperature Region
STA	Solution Treated and Aged
STEM	Scanning Transmission Electron Microscope
TEM	Transmission Electron Microscope
UTS	Ultimate tensile strength
V	Vertical
XRD	X-Ray Diffraction
YS	Yield Strength
WQ	Water Quenching

## Data Availability

All the data is available within the manuscript.
